# Auditory streaming emerges from fast excitation and slow delayed inhibition

**DOI:** 10.1186/s13408-021-00106-2

**Published:** 2021-05-03

**Authors:** Andrea Ferrario, James Rankin

**Affiliations:** Department of Mathematics, College of Engineering, Mathematics & Physical Sciences, University of Exeter, Exeter, UK

**Keywords:** Auditory streaming, Slow delayed inhibition, Fast excitation

## Abstract

In the auditory streaming paradigm, alternating sequences of pure tones can be perceived as a single galloping rhythm (integration) or as two sequences with separated low and high tones (segregation). Although studied for decades, the neural mechanisms underlining this perceptual grouping of sound remains a mystery. With the aim of identifying a plausible minimal neural circuit that captures this phenomenon, we propose a firing rate model with two periodically forced neural populations coupled by fast direct excitation and slow delayed inhibition. By analyzing the model in a non-smooth, slow-fast regime we analytically prove the existence of a rich repertoire of dynamical states and of their parameter dependent transitions. We impose plausible parameter restrictions and link all states with perceptual interpretations. Regions of stimulus parameters occupied by states linked with each percept match those found in behavioural experiments. Our model suggests that slow inhibition masks the perception of subsequent tones during segregation (forward masking), whereas fast excitation enables integration for large pitch differences between the two tones.

**Supplementary Information:**

The online version contains supplementary material available at 10.1186/s13408-021-00106-2.

## Introduction

Understanding how our perceptual system encodes multiple objects simultaneously is an open challenge in sensory neuroscience. In a busy room, we can separate out a voice of interest from other voices and ambient sound (*cocktail party problem*) [1, 2]. Theories of feature discrimination developed with mathematical models are based on evidence that different neurons respond to different stimulus features (e.g. visual orientation [3–6]). Primary auditory cortex (ACx) has a topographic map of sound frequency (tonotopy): a gradient of locations preferentially responding to frequencies from low to high [7, 8]. However, feature separation alone cannot account for the auditory system segregating objects overlapping or interleaved in time (e.g. melodies, voices). Understanding the role of temporal neural mechanisms in perceptual segregation presents an interesting modelling challenge where the same neural populations represent different percepts through temporal encoding.

### Auditory streaming and auditory cortex

**Figure 1:**
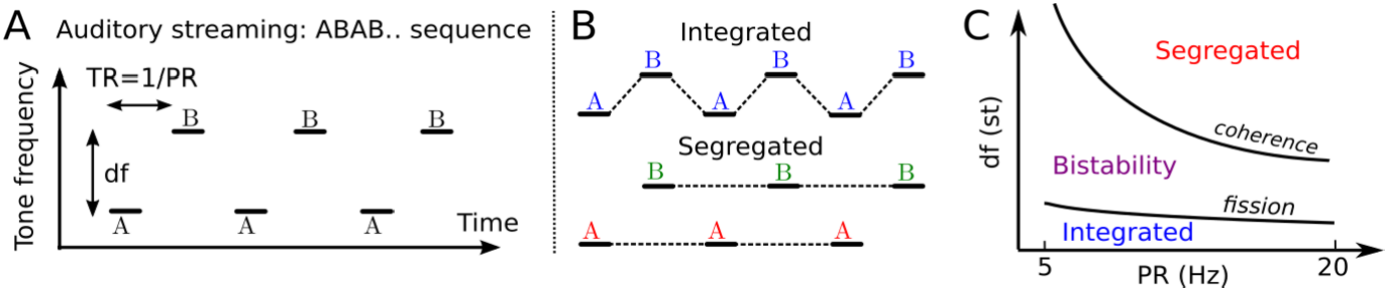
The auditory streaming paradigm. (**A**) Auditory stimuli consist of sequences of interleaved higher pitch A and lower pitch B pure tones with duration *TD*, pitch difference *df* and time difference between tone onsets *TR* (the repetition time; $PR=1/TR$ is the repetition rate). (**B**) The stimulus may be perceived as either an integrated ABAB stream or as two separate streams A-A- and -B-B. (**C**) Sketch of the perceptual regions when varying *PR* and *df* (van Noorden diagram), redrawn after [9]. Bistability corresponds to the perception of temporal switches between integration and segregation. The curves in the $(PR,df)$ space separating integration from bistability and bistability from segregation are called fission and coherence boundaries

In the auditory system, sequences of sounds (streams) that are close in feature space (e.g. frequency) and interleaved in time lead to multiple perceptual interpretations. The so-called auditory streaming paradigm [2, 9] consists of interleaved sequences of tones A and B, separated by a difference in tone frequency (called *df*) and repeating in an ABABAB…pattern (Fig. 1A). This can be perceived as one integrated stream with an alternating rhythm (Integrated in Fig. 1B) or as two segregated streams (Segregated in Fig. 1B). When *df* is small, we hear integrated, and when df is large, we hear segregated, but at an intermediate range, which also depends on presentation rate *PR*, both percepts are possible (Fig. 1C). In this region of $(\text{df},{PR})$, parameter space bistability occurs, where perception switches between integrated and segregated every 2–15 s [10]. The coherence and fission boundaries (Fig. 1C) are plotted for the same range of PRs typically considered in experiments (5–20 Hz, [9]). Below 5 Hz tones become isolated events not tracked as a rhythm, and above 20 Hz isochronal rhythms are perceived as pure tones in the first octave of human hearing (see Sect. 9).

**Figure 2:**
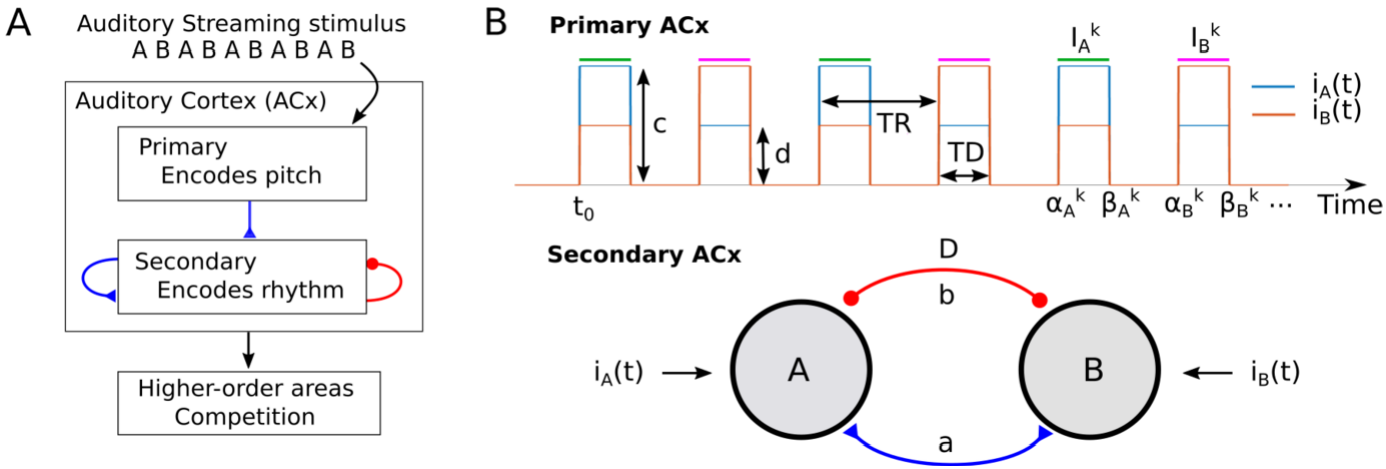
(**A**) Proposed modelling framework of the auditory streaming paradigm. Two-tone streams are processed in primary ACx. Seconday ACs receives inputs from primary areas and has recurrent excitatory and inhibitory connections. Primary and secondary areas encode respectively pitch and rhythm [11], whereas high-order cortical areas encode the perceptual switches via competition (bistability). (**B**) ACx circuit model. Primary ACx tonotopic responses consist of square-wave A and B tone inputs $i_{A}$ and $i_{B}$ with duration *TD* and with the time between tone onsets *TR* (called repetition time – the inverse of the presentation rate (PR)). Parameters *c* and *d* respectively represent the connection strength from $i_{A}$ ($i_{B}$) to the A (B) and B (A) units. Bottom: sketch of the model circuit consisting of two mutually excitatory and inhibitory populations with strengths *a* and *b*, respectively, receiving inputs $i_{A}$ and $i_{B}$. Inhibition is delayed of the amount *D*

Figure 2A shows our proposal for the encoding of auditory streaming. We follow the hypothesis proposed by [11], where primary and secondary ACx encode respectively perception of the pitch and the rhythm. In our proposed framework the processing of auditory stimuli occurs firstly in primary ACx, which encodes stimulus feature content across tonotopy along with onset/offset timing and projects to secondary ACx. We propose that the various rhythms perceived in the auditory streaming paradigm arise via recurrent connections in secondary ACx [12] and via threshold-crossing detection in the resulting activity. The specific rhythm perceived is determined downstream, that is, selected from those represented in secondary ACx, and the process underlying bistability is likely also resolved downstream [13]. These downstream computations are not addressed in the present study, but may involve top-down modulation of primary and secondary auditory cortices.

### Existing models of auditory streaming

Inspired by evidence of feature separation shown in neural recordings in primary auditory cortex (A1) [14], many existing models have sidestepped the issue of the temporal encoding of the perceptual interpretations by focusing on a feature representation (reviews: [13, 15, 16]). Neurons responding primarily to the A or to the B tones are in adjacent locations, spatially separated along A1’s tonotopic axis. The so-called neuromechanistic model [17] proposed the encoding of percepts based on discrete, tonotopically organised units interacting through plausible neural mechanisms. Models proposed in a neural oscillator framework feature significant redundancy in their structure or work only at specific presentation rate (PR) values [18, 19]. Temporal forward masking results in weaker responses to similar sounds that are close in time (at high PR), but this ubiquitous feature of the auditory system [20] has been overlooked in previous models.

### Theoretical framework

The cortical encoding of sensory information involves large neural populations suitably represented by coarse-grained variables like the mean firing rate. The Wilson–Cowan equations [21] considered here describe neural populations with excitatory and delayed inhibitory coupling. Variants of these equations include networks with excitatory and inhibitory coupling, synaptic dynamics that include neural adaptation, nonlinear gain functions [22–24] and symmetries [25, 26]. This framework (and related voltage- or conductance-based formulations) are widely used to study, for example, decision making [27], perceptual competition in the visual [25, 28, 29] and in the auditory system [17].

A range of neural and synaptic activation times often leads to timescale separation [30–32] as considered here. Singular perturbation theory has been instrumental in revealing the dynamic mechanisms behind neural behaviours involving a slow-fast decomposition, for example, the generation of spiking and bursting [31, 33], neural competition [24, 34] and rhythmic behaviours [35, 36]. In this work, we use these techniques to determine the existence conditions of various dynamical states.

We consider the role of delayed inhibition in generating oscillatory activity compatible with auditory percepts. Delayed inhibition produces similar patterns of in- and anti-phase oscillations in spiking neural models [37, 38]. Delays in small neural circuits [39] lead to many interesting phenomena including inhibition-induced oscillations, oscillator death and switching between oscillatory solutions [40, 41]. Two novel features of our study are that the units are not intrinsically oscillating and that periodic forcing drives oscillations. Periodically forced, timescale separated models of perceptual competition [19, 29, 42] typically do not feature delays.

### Outline

With the aim of clarifying a plausible model for the processing of ambiguous sounds we present a biologically inspired neural circuit in ACx with mixed feature and temporal encoding that captures the auditory streaming phenomena. The model consists of two coupled neural populations with fast direct excitation and slow delayed inhibition (Sect. 2). Section 3 describes simulations of model states linked to percepts in the auditory streaming paradigm. Later sections are devoted to derive analytically conditions for the existence of all possible states in a non-smooth, slow-fast regime under plausible parameter constraint. The complete proofs are given in the Supplementary Material 1 for the interested reader. In Sect. 4, we dissect the model into slow and fast subsystems and analyze quasi-equilibria of the fast subsystem. We use this analysis in Sects. 5 and 6 and classify dynamical states using a binary matrix representations (*matrix form*). This tool enables us to determine all periodic states, their existence conditions and rule out which states are impossible. Sections 7 and 8 classify periodic states for long and short inhibitory delays, respectively. Lastly, in Sect. 9, we show numerically how these results extend to a smooth setting with reduced timescale separation. When applied to study the auditory streaming paradigm, these methods suggest how competing perceptual interpretations emerge as a result of mutual excitation and slow delayed inhibition in tonotopically localized units in a non-primary part of auditory cortex.

## The mathematical model

We present a model for the encoding of different perceptual interpretations of the auditory streaming paradigm. Following our proposal of rhythm and pitch perception (Fig. 2A), we consider a periodically driven competition network of two localised Wilson–Cowan units (Fig. 2B) with lumped excitation and inhibition generalised to include dynamics via inhibitory synaptic variables. The units A and B are driven by a stereotyped input signals $i_{A}$ and $i_{B}$ representative of neural responses in primary auditory cortex [14] at tonotopic locations that preferentially respond to A and to B tones, respectively (Fig. 2B). The model is described by the following system of DDEs:

1 $$ \begin{gathered} \tau \dot{u}_{A}(t) = -u_{A}(t) + H\bigl(a u_{B}(t)- b s_{B}(t-D)+ i_{A}(t)\bigr), \\ \tau \dot{u}_{B}(t) = -u_{B}(t) + H\bigl(a u_{A}(t)- b s_{A}(t-D)+ i_{B}(t)\bigr), \\ \dot{s}_{A}(t) = H\bigl(u_{A}(t)\bigr) \bigl(1-s_{A}(t) \bigr)/\tau -s_{A}(t)/\tau _{i}, \\ \dot{s}_{B}(t) = H\bigl(u_{B}(t)\bigr) \bigl(1-s_{B}(t) \bigr)/\tau -s_{B}(t)/\tau _{i}, \end{gathered} $$

where units $u_{A}$ and $u_{B}$ represent the average firing rate of two neural populations encoding sequences of tone (sound) inputs with timescale *τ*. The Heaviside gain function with activity threshold $\theta \in (0,1)$: $\{H(x)=1\text{ if }x \geq \theta \text{ and 0 otherwise} \}$ is widely used in firing rate and neuronal field models [24, 43] (we later relax this assumption to consider a smooth gain function). Mutual coupling through direct fast excitation has strength $a \geq 0$. The delayed, slowly decaying inhibition has timescale $\tau _{i}$, strength $b \geq 0$ and delay *D* (Fig. 2A). The synaptic variables $s_{A}$ and $s_{B}$ describe the time-evolution of the inhibitory dynamics. Typically we will assume $\tau _{i}$ to be large and *τ* to be small. This slow-fast regime and the choice of a Heaviside gain function allows for the derivation of analytical conditions for the existence of biologically relevant network states.

### Model inputs

Psychoacoustic experiments typically consider pure tone frequencies above 0.5 kHz (where primary ACx responses reflect onsets and offsets of tones without following the sinusoidal tone modulation). Each frequency (tone) in the ACx is encoded by the neural activity at a specific *best frequency* spatial location. This spatial organization is ordered so that pairs of tones with similar frequencies are encoded by the neural activity of neighbouring sites (so-called “tonotopy”). Auditory streams consisting of interleaved A and B tones evoke periodic onset-platau primary ACx responses at A and B best frequency locations [14, 44, 45]. These responses broadly look like the periodic square wave input functions $i_{A}(t)$ and $i_{B}(t)$ considered in our study, which represent the averaged excitatory synaptic currents from primary ACx at A and B locations (Fig. 2B, top). We note that these functions characterize responses to tones in primary ACx (from experiments [14]) rather than the sound waveform of the tone sequences (motivated in Sect. 3) and are defined by

2 $$ \begin{gathered} i_{A}(t) = c\sum _{k=0}^{\infty } \chi _{I_{A}^{k}} (t) + d \sum _{k=0}^{ \infty } \chi _{I_{B}^{k}} (t), \\ i_{B}(t) = d \sum_{k=0}^{\infty } \chi _{I_{A}^{k}} (t) + c \sum_{k=0}^{ \infty } \chi _{I_{B}^{k}} (t), \end{gathered} $$

where $c\geq 0$ and $d\geq 0$ represent the input strengths from A (B) tonotopic location respectively to the A (B) unit and to the B (A) unit; $\chi _{I}$ is the standard indicator function over the set *I*, defined as $\chi _{I}(t)=1$ for $t \in I$ and 0 otherwise. We impose the condition $c\geq d$, which guarantees stronger A (B) tones responses at A (B) unit and weaker responses to the B (A) unit, following the tonotopy hypothesis. The intervals when A and B tones are on (active tone intervals) are respectively $I_{A}^{k}=[\alpha _{k}^{A},\beta _{k}^{A}]$ and $I_{B}^{k}=[\alpha _{k}^{B},\beta _{k}^{B}]$ (see Fig. 2B, top) and given by

$$ \alpha _{A}^{k}=2kTR, \qquad \beta _{A}^{k}=2kTR+TD, \qquad \alpha _{B}^{k}=(2k+1)TR, \qquad \beta _{B}^{k}= (2k +1)TR+TD, $$

where the parameter *TD* represents the duration of each tone’s presentation (see Discussion for another interpretation of *TD*), and *TR* is the time between tone onsets (called repetition time; $PR=1/TR$ is the presentation rate). We selected a value of *TD* so that the square wave ON time captures the width of the onset response from [14]. Let us denote the set of active tone intervals *R* and its union *I* by

$$ \Phi =\bigl\{ R \subset \mathbb{R} : R=I_{k}^{A} \text{ or } R=I_{k}^{B}, \forall k \in \mathbb{N} \bigr\} \quad \text{and} \quad I=\bigcup_{R \in \Phi }R. $$

As shown in Fig. 1, the parameters *TD* and *PR* play an important influence on auditory streaming [14]. We consider $PR \in [1,40]\text{ Hz}$, $TR\geq TD$ and $TR\geq D$, where *D* is the inhibitory delay. These restrictions are typical conditions tested in psychoacoustic experiments. In particular, $TR\geq TD$ guarantees no overlaps between tone inputs, that is, $I_{A}^{i} \cap I_{B}^{j}=\emptyset $ for $i,j \in \mathbb{N}$.

#### Remark 2.1

(Constraining model parameters)

Throughout this work, we assume the following conditions:

($U _{1}$)
$a-b < \theta $,
($U _{2}$)
$c \geq \theta $.

Condition ($U _{1}$) guarantees that the point $P=(0,0,0,0)$ is the only equilibrium of system (1) with no inputs ($i_{A}=i_{B} =0$), thus avoiding trivial saturating dynamics. Indeed, assuming *τ* sufficiently small and a Heaviside gain function *H*, this system has two equilibrium points, a quiescent state $P=(0,0,0,0)$ and an active state $Q=(1,1,1,1)$. If the difference between excitatory and inhibitory strengths $a-b\geq \theta $, then *P* and *Q* coexist, and any trajectory of the non-autonomous system is trivially determined by the input strength *c*:

- If $c<\theta $, then any trajectory starting from the basin of attraction of *P* (or *Q*) quickly converges to *P* (*Q*) and remains at this equilibrium.
- If $c\geq \theta $, then any trajectory converges to *Q* and remains at this equilibrium. Indeed, if an orbit is in the basin of *P*, then the synaptic variables monotonically decrease until one unit turns ON. This turns ON the other unit (since $a-b\geq \theta $), and both units remain ON.

Condition ($U _{2}$) guarantees non-trivial dynamics during the active tone intervals. Indeed, as we will show in Lemma 3, both units are OFF at the start time *t* each active tone interval. The total input to unit A is $c-bs_{B}(t-D)\leq c$, and the one to unit B is $c-bs_{B}(t-D)\leq d\leq c$. Therefore, if $c<\theta $, then no unit can turn ON at this or any other time in any active tone interval.

## A motivating example

We now present examples of the type of responses studied throughout this work using a smooth version of model (1) and by proposing a link between these responses and the different percepts in the auditory streaming experiments. We use a sigmoid gain function $S(x)=[1+\exp (-\lambda x)]^{-1}$ with fixed slope $\lambda =30$. Inputs in equation (2) are made continuous using function *S* by redefining them as

3 $$\begin{aligned} \begin{aligned} I_{A}(t) = c \cdot p(t)p(TD-t) + d \cdot q(t)q(TD-t), \\ I_{B}(t) = d \cdot p(t)p(TD-t) + c \cdot q(t)q(TD-t), \end{aligned} \end{aligned}$$

where $p(t)=S(\sin (\pi PR\cdot t))$ and $q(t)=S(-\sin (\pi PR\cdot t))$, so that the component $p(t)p(TD-t)$ ($q(t)q(TD-t)$) represents the responses to A (B) tone inputs with duration *TD*. These inputs are similar to the discontinuous input shown in Fig. 2B but with smooth ramps at the discontinuous jump up and jump down points.

Psychoacoustic experiments analysed the changes in perceptual outcomes when varying input parameters *PR* and *df* (Fig. 1C). The parameter *PR* is encoded in the model inputs’ repetition rates. To model the parameter *df*, we take into account the experimental recordings of the average spiking activity from the primary ACx of various animals (macaque [14, 44], guinea pigs [46]). These show that the activity at A tonotopic locations decreases nonlinearly with *df* during B tone presentations. We thus assume that the input strength *d* can be scaled by *df* according to $d=c \cdot (1-df^{1/m})$, where *m* is a positive integer, and *df* is a unitless parameter in $[0,1]$, which may be converted to semitone units using the formula $12\log (1+df)$.

**Figure 3:**
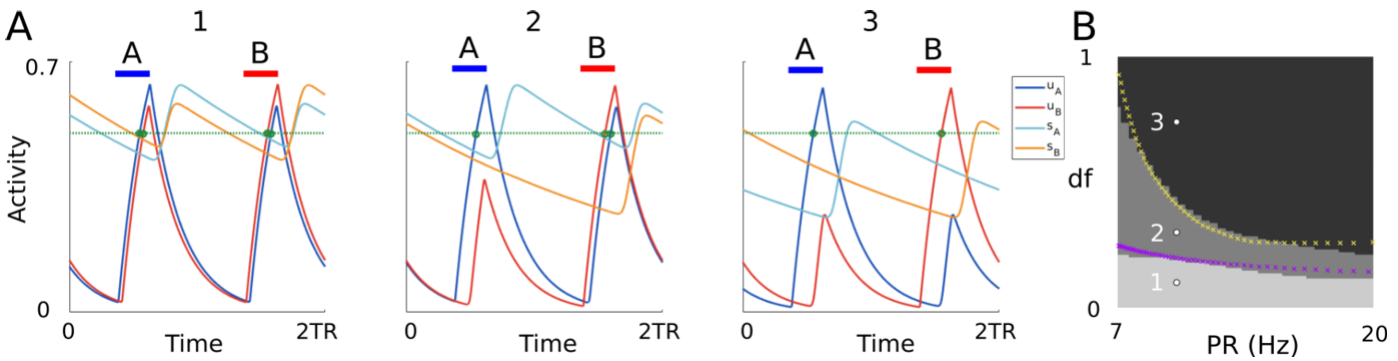
(**A**) Time histories of the $2TR$-periodic states in system (1). Active tone A and B intervals are shown by blue and red bars, respectively. Units’ threshold crossings are shown by green dots. (**B**) The total number of threshold crossings for both units is shown in greyscale for simulated trajectories at varying *PR* and *df* (black =2, lightest gray =4 crossings). Parameters *PR* and *df* in panel (**A**) are shown by white dots in panel (**B**). Yellow and purple crosses are the experimentally detected coherence and fission boundaries, respectively (data replotted from Fig. 2 in [47], digitalized using the software WebPlotDigitizer [48]). The remaining parameters are $a=2$, $b=2.8$, $c=5.5$, $D=0.015$, $\theta =0.5$, $TD=0.022$, $\tau _{i}=0.25$, $\tau =0.025$, $\theta =0.5$ and $m=6$. Simulations are performed using dde23 in Matlab with absolute and relative tolerances set to 10^−7^. Initial conditions on the interval $[-D,0]$ are specified as a constant vector function equal to $[1,0,1,0]$

Figure 3A shows simulated time histories of all the $2TR$-periodic states for different values of parameters $(PR,df)$, where all the other parameters are fixed. Blue and red bars indicate the A and B active tone intervals $[0,TD]$ and $[TR,TR+TD]$, respectively, to show when the inputs are on. The system exhibits one of three possible behaviours/states: (1) both units cross threshold (total of 4 crossings), (2) the A unit crosses threshold twice and the B unit once (total of 3 crossings), and (3) both units cross threshold once (total of 2 crossings). We then summarize the effect of parameters $(PR,df)$ on the convergence to the different attractors by running massive simulations at varying parameters $(PR,df)$ and counting the number of threshold crossings (Fig. 3B). States (1)–(3) belong to one of the grey regions in Fig. 3B. We note that state (2) coexists with its complex conjugate state, for which the B unit crosses threshold twice and the A unit once (not shown).

We propose a link between these states and the different percepts emerging in auditory streaming (integration, segregation and bistability), where rhythms are tracked by responding (threshold crossing) in the A and B units’ activities of $2TR$-periodic states. More precisely:

- Integration corresponds to state (1): both units respond to both tones.
- Bistability corresponds to state (2): one unit responds to both tones, and the other unit responds to only one tone.
- Segregation corresponds to state (3): no unit responds to both tones.

Following this proposal, the states (1)–(3) match the regions of existence of their equivalent percepts. The transition boundaries between these states fit with the fission and coherence boundaries found experimentally (Fig. 3B). In the next sections, we take an analytical approach to study the model’s states and their existence conditions. This approach allows us to derive expressions for the fission and coherence boundaries (equations (20) in Sect. 8.3) in a mathematically tractable version of the model (2). Quantitative comparisons between the analytical and computational approaches are discussed in Sect. 9.

## Fast dynamics

In this and the next sections (until Sect. 9), we present analytical results of the fast subsystem (4) with Heaviside gain. System (1) can be decoupled into slow and fast subsystems. The fast subsystem is given by

4 $$ \begin{gathered} u_{A}(r)' = -u_{A}(r) + H\bigl(a u_{B}(r)- b s_{B}(r-D)+i_{A}(r) \bigr), \\ u_{B}(r)' = -u_{B}(r) + H\bigl(a u_{A}(r)- b s_{A}(r-D)+i_{B}(r)\bigr), \\ s_{A}(r)' = H\bigl(u_{A}(r)\bigr) \bigl(1-s_{A}(r)\bigr), \\ s_{B}(r)' = H\bigl(u_{B}(r)\bigr) \bigl(1-s_{B}(r)\bigr), \end{gathered} $$

where $'=d/dr$ is the derivative with respect to the fast scale $r=t/\tau $. Activities $u_{A}$ and $u_{B}$ take a value of 0 or 1, or move rapidly (on the fast time scale) between these two values. We call A(B) ON if $u_{A} \sim 1$ and OFF if $u_{A} \sim 0$. The activity of the A (B) unit is determined by the sign of quantities $a u_{B}(t)- b s_{B}(t-D)+i_{A}(t)$ ($a u_{A}(t)- b s_{A}(t-D) +i_{B}(t)$). Positive sign changes make $u_{A}$ ($u_{B}$) jump up from 0 to 1 (turn ON), whereas negative sign changes make $u_{A}$ ($u_{B}$) jump down from 1 to 0 (turn OFF). The synaptic variables can act on either the fast or the slow time scales. If A (B) is ON, then the variable $s_{A}$ ($s_{B}$) jumps to 1 on the fast time scale. If A (B) is OFF, then the dynamics of $s=s_{A}$ (or $s=s_{B}$) slowly decay according to

5 $$ \dot{s}=-s/\tau _{i}. $$

### Remark 4.1

The previous considerations demonstrate that $s_{A}(t)$ ($s_{B}(t)$) is a monotonically decreasing in time, except for when the A (B) unit makes an OFF to ON transition.

We proceed by analyzing system (4) for $t \in I$, that is, in one of the active tone intervals. From the definition of *I* we assume that $t \in I_{k}^{A}$, a generic A tone interval. The analysis below can easily be extended for B tone intervals $I_{k}^{B}$ by swapping the parameters *c* and *d*. On the fast time scale the A and B unit satisfy the subsystem

6 $$ \begin{gathered} u_{A}' = -u_{A} + H(au_{B}-b \tilde{s}_{B}+c), \\ u_{B}' = -u_{B} + H(au_{A}-b \tilde{s}_{A}+d), \end{gathered} $$

where $\tilde{s}_{A}=s_{A}(t-D)$ and $\tilde{s}_{B}=s_{B}(t-D)$. System (6) has four equilibrium points: (0,0), (1,0), (0,1) and (1,1), and their existence conditions are reported in Table 1.

**Table 1:** Equilibria and existence conditions for the fast subsystem (6)

Equilibrium	(0,0)	(1,0)	(0,1)	(1,1)
Conditions	$\begin{array}{l} c< b \tilde{s}_{B}+\theta \\ d< b \tilde{s}_{A}+\theta \end{array} $	$\begin{array}{l} c \geq b \tilde{s}_{B}+\theta \\ a+d < b \tilde{s}_{A}+\theta \end{array} $	$\begin{array}{l} a+c < b \tilde{s}_{B}+\theta \\ d \geq b \tilde{s}_{A}+\theta \end{array} $	$\begin{array}{l} a+c \geq b \tilde{s}_{B}+\theta \\ a+d \geq b \tilde{s}_{A}+\theta \end{array} $

The full system (1) may jump between these equilibria due to the slow decay of the synaptic variables or when $s_{A}(t-D)$ and $s_{B}(t-D)$ jumps up to 1.

### Basins of attraction

**Figure 4:**
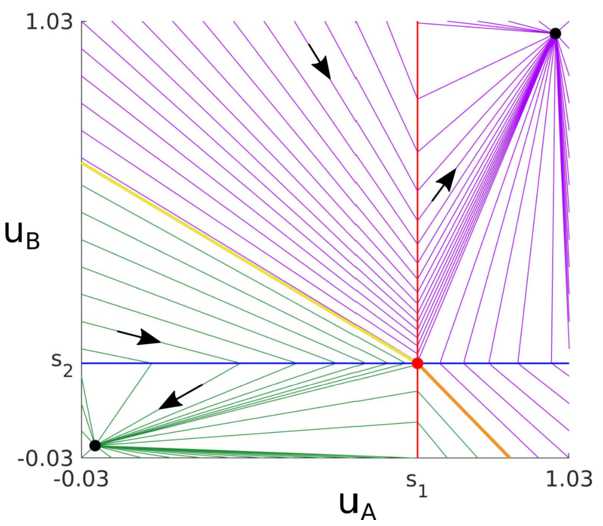
Phase portrait for system (6). Purple and green lines show orbits converging to stable equilibria $(1,1)$ and $(0,0)$, respectively (black circles). Black arrows indicate the direction of convergence. The $u_{A}$- and $u_{B}$-nullclines are shown in blue and red, respectively. Yellow and orange lines show the separatrices of the degenerate saddle $(s_{1},s_{2})$ (red circle), where $s_{1}=(b \tilde{s}_{A}-c+\theta )/a$ and $s_{2}=(b \tilde{s}_{B}-c+\theta )/a$. More details are in the Supplementary Material 1.1

From the inequalities given in Table 1 we note that points $(1,0)$ and $(0,1)$ cannot coexist with any other equilibrium and thus have trivial basins of attraction. However, $(0,0)$ and $(1,1)$ may coexist under specific conditions, with a degenerate saddle separatrix dividing the basin of attraction of these two equilibria (Fig. 4). Similar equilibria, separatrices and basin of attractions occur with continuous (steep) sigmoidal gains. The study of the basin of attraction, equilibria and separatrices of the fast subsystem (6) is in the Supplementary Material 1.1.

### Differential convergence to $(1,1)$

We now study the differential rate of convergence of the variables $u_{A}$ and $u_{B}$ with parameter values where $(1,1)$ is the only stable equilibrium for an orbit starting from $(0,0)$. We will use the results below to classify of states of system (1). For simplicity, we consider the case $t \in I_{A}^{k}$, as in system (6). Similar considerations hold in the case $t \in I_{B}^{k}$. Obviously, $(0,0)$ cannot be an equilibrium, and thus at least one of the two conditions in Table 1 must not be met. There are three cases to consider:

1. If $c-b\tilde{s}_{B}\geq \theta $ and $d-b\tilde{s}_{A}\geq \theta $, then both units turn ON simultaneously, each following the same dynamics $u'=1-u$. An orbit starting from $(0,0)$ must therefore reach $(1,1)$ under the same exponential rate of convergence.
2. If $c-b\tilde{s}_{B}\geq \theta $, $d-b\tilde{s}_{B}<\theta $ and $a+d-b\tilde{s}_{A}\geq \theta $, then unit B turns ON after A by some small delay *δ* (∼*τ*). Indeed, from $d-b\tilde{s}_{B}<\theta $ and $a+d-b\tilde{s}_{A}\geq \theta $ it follows that there is $u^{*} \in (0,1]$ such that $au_{*}+d-b\tilde{s}_{A}=\theta $. Since $c-b\tilde{s}_{B}\geq \theta $, the fast subsystem reduces to
  $$ \begin{gathered} u_{A}' = 1-u_{A}, \\ u_{B}' = -u_{B}+H(au_{A}-b \tilde{s}_{A}+d) \stackrel{\mathrm{def}}{=}-u_{B}+\eta (u_{A}). \end{gathered} $$
  Thus the dynamics of $u_{A}$ is independent of $u_{B}$. Consider an orbit starting from $(0,0)$ at $r=0$. From the first equation $u_{A}(r)$ tends to 1 exponentially as $r \rightarrow \infty $, reaching a point $u^{*}$ at time $r^{*}=\log [(1-u^{*})^{-1}]$. For $r< r^{*}$, we have $u_{A}(r)< u^{*}$, which yields $\eta (u_{A}(r))=0$. Since the orbit starts from $u_{B}=0$, it must remain constant and equal to zero for all $r< r^{*}$. For $r\geq r^{*}$, $\eta (u_{A}(r))=1$ and $u_{A}(r) \rightarrow 1$ following the same dynamics as $u_{A}$ at time $r=0$. On the time scale $t=\tau r$ of system (1), the A unit precedes the B unit in converging to 1 precisely after an infinitesimal delay
  7 $$ \delta =\tau \log \bigl[\bigl(1-u^{*}\bigr)^{-1}\bigr]. $$
3. The case $d-b\tilde{s}_{A}\geq \theta $, $c-b\tilde{s}_{A}<\theta $ and $a+c-b\tilde{s}_{B}\geq \theta $ is analogous to the previous after replacing $u_{A}$ with $u_{B}$. In this case, A turns ON a delay *δ* after B.

### Fast dynamics for $t \in \mathbb{R}-I$

The analysis for times when inputs are OFF ($t \in \mathbb{R}-I$) follows analogously by posing $c=d=0$ in system (6) and counts only two possible equilibria, $(0,0)$ and $(1,1)$. Point $(0,0)$ is an equilibrium for any values of parameters and delayed synaptic quantities $\tilde{s}_{A}$ and $\tilde{s}_{B}$. Instead, $(1,1)$ is an equilibrium when

$$ a-b\tilde{s}_{A} \geq \theta \quad \text{and} \quad a-b \tilde{s}_{B} \geq \theta . $$

## Dynamics in the intervals with no inputs ($\mathbb{R}-I$)

The study of equilibria for the fast subsystem described so far constraints the dynamics of the full system in the intervals with no inputs, that is, in $\mathbb{R}-I$. The first constraint is that the units can either be both ON, both OFF, or both turning OFF at any time in $\mathbb{R}-I$ (Theorem 1).

### Theorem 1

(Dynamics in $\mathbb{R}-I$)

*For any* $t \in \mathbb{R}-I$:

1. *If A or B is OFF at time* *t*, *then both units are OFF in* $(t,t^{*}]$, *where*
  $$ t^{*}=\min_{s \in I} \{s > t \}. $$
2. *If A or B is ON at time* *t*, *then both units are ON in* $[t_{*},t)$, *where*
  $$ t_{*}=\max_{s \in I} \{s < t \}. $$

**Figure 5:**
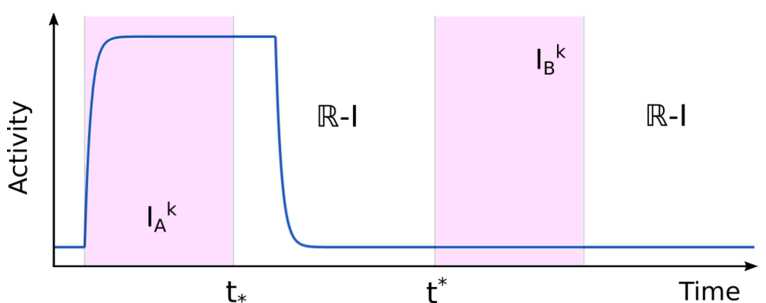
Illustration of Theorem 1 showing one unit’s dynamics (blue) during one $2TR$ period. Active tone intervals $I_{A}^{k}$ and $I_{B}^{k}$ are shown in purple. Note: the unit turns OFF at some time in $[t_{*},t^{*}]$ due to the delayed inhibition from the the other unit, whose activity is omitted

This theorem is proved in the Supplementary Material 1.2 and illustrated with an example in Fig. 5. Due to this theorem, we can classify network states as follows.

### Definition 5.1

(LONG and SHORT states)

We define any state of system (1):

- LONG if there is $t \in \mathbb{R}-I$ when both units are ON,
- SHORT if both units are OFF for all $t \in \mathbb{R}-I$.

The choice of the names LONG and SHORT is derived from the following considerations. Since both units are ON at some time $t \in \mathbb{R}-I$ of a LONG state, Theorem 1 implies they must be ON at the end of the active tone interval preceding *t* and prolong their activation after the active tone interval up to time *t*. SHORT states by definition are OFF between each pair of successive tone intervals.

**Figure 6:**
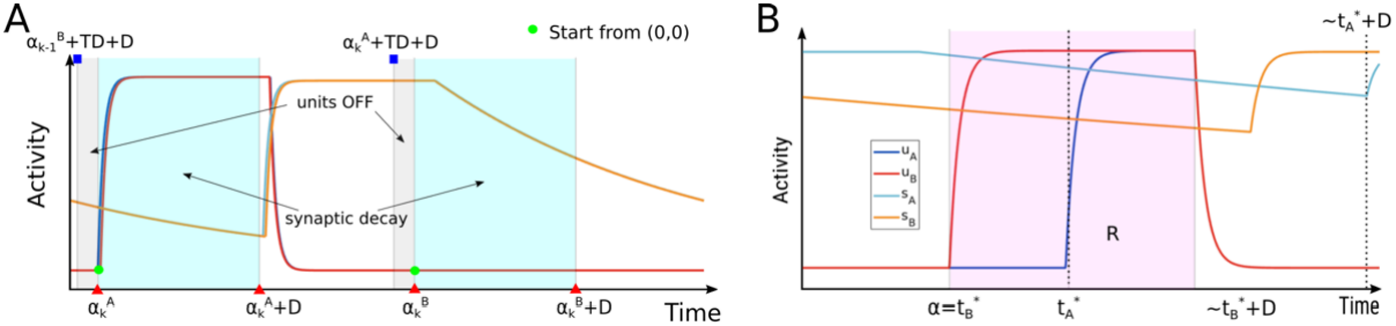
(**A**) Example dynamics of the A and B units in each interval $L \subset \Gamma $ and *J* illustrating Lemmas 2 and 4 during one period $2TR$. (**B**) Dynamics in an active tone interval $R=[\alpha ,\beta ] \in \Phi $ illustrating the quantities in (1)–(3) of Lemma 4, where $t_{A}^{*}$ ($t_{B}^{*}$) is the turning ON time for A (B)

Theorem 1 guarantees either that unit can turn ON only during an active tone interval. This guarantees that the delayed synaptic variables are monotonically decreasing in the intervals $[\alpha _{k}^{A},\alpha _{k}^{A}+D]$ and $[\alpha _{k}^{B},\alpha _{k}^{B}+D]$ if the condition $TD+D< TR$ is guaranteed. The latter theorem is proven in the Supplementary Material 1.3 and is illustrated in Fig. 6A.

### Lemma 2

(Synaptic decay)

*If* $TD+D< TR$, *then the delayed synaptic variables* $s_{A}(t-D)$ *and* $s_{B}(t-D)$ *are monotonically decreasing in* $[\alpha _{k}^{A},\alpha _{k}^{A}+D]$ *or* $[\alpha _{k}^{B},\alpha _{k}^{B}+D]$ *for all* $k \in \mathbb{N}$.

A second important implication of Theorem 1 under $TD+D< TR$ is that both units must turn OFF once between successive tone intervals (see the next lemma). This guarantees that at the start of each active tone interval, any state of the fast subsystem start from point $(0,0)$. The following lemma is proven in the Supplementary Material 1.4 and is illustrated in Fig. 6A.

### Lemma 3

(No saturated states)

*If* $TD+D< TR$, *then both units are OFF in the intervals* $(\alpha _{k}^{A}+TD+D,\alpha _{k}^{B}]$ *and* $(\alpha _{k}^{B}+TD+D,\alpha _{k+1}^{A}]$ *for all* $k \in \mathbb{N}$.

## Dynamics during the active tone intervals

We now study the possible dynamics of the full system during the active tone intervals $R \in \Phi $ under the condition $TD+D< TR$, for which Lemmas 2 and 3 can be applied. We split this analysis by separating the cases $D>TD$ and $D\leq TD$. In this section, we consider the case $D>TD$, and the other condition is considered in Sect. 8. The next lemma shows that the turning ON times of either unit can happen only at most once in *R* and other results, which lead to the existence of only a limited number of states.

### Lemma 4

(Single OFF to ON transition)

*Consider an active tone interval* $R=[\alpha ,\beta ] \in \Phi $, *and let A* (*B*) *be ON at a time* $\bar{t} \in R$. *Then*:

(1) *A* (*B*) *is ON for all* $t \geq \bar{t}$, $t \in R$;

(2) *There is a unique* $t_{A}^{*} \ (t_{B}^{*}) \in R$ *when A* (*B*) *turns ON*;

(3) $s_{A}(t-D)$ ($s_{B}(t-D)$) *is decreasing for* $t \in [\alpha ,t_{A}^{*}+D]$ $(t \in [\alpha ,t_{B}^{*}+D])$.

The previous lemma is illustrated in the cartoon shown in Fig. 6, right. The proof is given in the Supplementary Material 1.5 and implies the following lemma.

### Lemma 5

*Given any active tone interval* $R \in \Phi $, *we have*:

1. *A* (*B*) *turns ON at time* *α*⇔ *A* (*B*) *is ON for all* $t \in (\alpha ,\beta ]$,
2. *A* (*B*) *is OFF at time* *β*⇔ *A* (*B*) *is OFF for all* $t \in R$.

Due to Lemma 4, each unit may turn ON only once during each interval $R \in \Phi $. Thus the dynamics of any state is determined precisely at the jump up points $t_{A}^{*}$ and $t_{B}^{*}$ for the units in *R* (if these exist).

### Definition 6.1

(MAIN and CONNECT states)

A state (solution) of system (1) is:

- MAIN if $\forall R \in \Phi $, if $\exists t^{*} \in R$ turning ON time for A or B, then $t^{*} = \min (R)$;
- CONNECT if $\exists R \in \Phi $ and $\exists t^{*} \in R$, $t^{*}>\min (R)$ turning ON time for A or B.

**Figure 7:**
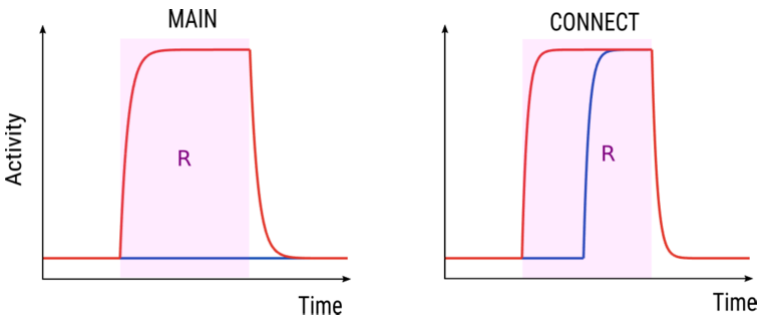
Example dynamics of the $u_{A}$ (red) and $u_{B}$ (blue) variables for MAIN and CONNECT states in an interval $R \in \Phi $. The left panel shows a MAIN state for which the A unit is OFF in *R*, whereas the B unit turns ON at time $t^{*}=\min (R)$. The right panel shows a CONNECT state for which the A unit turns ON at some time $t^{*}>\min (R)$, whereas the B unit turns ON at time $\min (R)$

Example time histories of a MAIN state and a CONNECT state during a generic active tone interval *R* is shown in Fig. 7.

### Remark 6.1

MAIN states are either ON or OFF during any interval $R \in \Phi $, except (possibly) for a negligible interval of length ∼0. Indeed due to differential convergence (Sect. 4.2), one unit may turn ON at time *α* following an infinitesimally small delay $\delta \sim \tau $, where *δ* is given by equation (7).

### Classification of MAIN and CONNECT states – matrix form

The results reported in the previous section the possible dynamics during each active tone interval $R \in \Phi $. In this section, we use these results to propose a classification of MAIN and CONNECT states based on their dynamics during these intervals and define the existence conditions for these states.

Due to Lemmas 3, 4 and 5, the units of any state must be OFF at the start *R* (orbits $(u_{A},u_{B})$ always start from $(0,0)$ at time *α*), a unit may turn ON at most once in *R*, and if this occurs, then it must remain ON until the end of *R*. Thus we have three possibilities: (1) both units are OFF in *R*, (2) only one unit turns ON once in *R*, or (3) both units turn ON once in *R*. These possibilities guarantee that any state in the network can be classified as MAIN or CONNECT. We note that condition ($U _{2}$) guarantees that (1) cannot occur for any $R \in \Phi $. Indeed, if a state’s unit A (B) is OFF for all A (B) in active tone interval *R*, then the delayed synaptic variables slowly converge to 0 starting from their initial value following (5). The total input $c-bs_{A}(t-D)$ of unit A in *R* converges to *c*. This is absurd since $c\geq \theta $. Let us define the inputs to the units for the A and B in active tone intervals as functions of the synaptic quantity *s*:

8 $$ f(s)= \textstyle\begin{cases} c-bs & \text{if } R=I_{A}^{k}, \\ d-bs & \text{if } R=I_{B}^{k}, \end{cases}\displaystyle \qquad g(s)= \textstyle\begin{cases} d-bs & \text{if } R=I_{A}^{k}, \\ c-bs & \text{if } R=I_{B}^{k}. \end{cases} $$

*Classification of MAIN states.* From the considerations given above the units’ dynamics in $R= [\alpha ,\beta ]$ of any MAIN state are completely determined on the fast time scale at times *α* and *β*. Each unit can either turn ON at time *α* or be OFF at time *β*, depending on the system parameters and on the following quantities:

$$ \underline{s}_{A}=s_{A}(\alpha -D), \qquad \bar{s}_{A}= s_{A}(\beta -D), \qquad \underline{s}_{B}=s_{B}( \alpha -D), \qquad \bar{s}_{B}=s_{B}(\beta -D). $$

Following the fixed point analyses, we consider three conditions (Table 2):

- *Both units turn ON at time* *α*. This is equivalent to $(1,1)$ being the only equilibrium for the fast subsystem at time *α*, which may occur under the conditions $M_{1-3}$. In summary, for case $M_{1}$, both units instantaneously turn ON at time *α*. For case $M_{2}$ ($M_{3}$), unit B (A) turns ON after A (B) of an infinitesimal delay $\delta \sim \tau $ (see Sect. 4.2).
- *One unit turns ON at time* *α, and the other unit is OFF at time* *β*. This corresponds to states satisfying one of conditions $M_{4-5}$. For case $M_{4}$ ($M_{5}$), A(B) turns ON at *α*, and B(A) is OFF at *β*. Indeed, $(1,0)$ ($(0,1)$) is the only stable equilibrium of the subsystem at times *α* and *β*, and thus for all $t \in R$ due to Lemma 5.
- *A and B are OFF at time* *β*. This occurs when $(0,0)$ is the only stable equilibrium of the fast subsystem at time *β*, thus satisfying condition $M_{6}$.

**Table 2:** Existence conditions for MAIN states in an interval $R \in \Phi $

$M_{1}$	$M_{2}$	$M_{3}$	$M_{4}$	$M_{5}$	$M_{6}$
$\begin{array}{l} f(\underline{s}_{B}) \geq \theta \\ g(\underline{s}_{A}) \geq \theta \end{array} $	$\begin{array}{l} g(\underline{s}_{A})<\theta \\ f(\underline{s}_{B})\geq \theta \\ a+g(\underline{s}_{A})\geq \theta \end{array} $	$\begin{array}{l} f(\underline{s}_{B})<\theta \\ g(\underline{s}_{A})\geq \theta \\ a+f(\underline{s}_{B})\geq \theta \end{array} $	$\begin{array}{l} f(\underline{s}_{B})\geq \theta \\ a+g(\bar{s}_{A})<\theta \end{array} $	$\begin{array}{l} g(\underline{s}_{A})\geq \theta \\ a+f(\bar{s}_{B})<\theta \end{array} $	$\begin{array}{l} g(\bar{s}_{A})<\theta \\ f(\bar{s}_{B})<\theta \end{array} $

**Figure 8:**
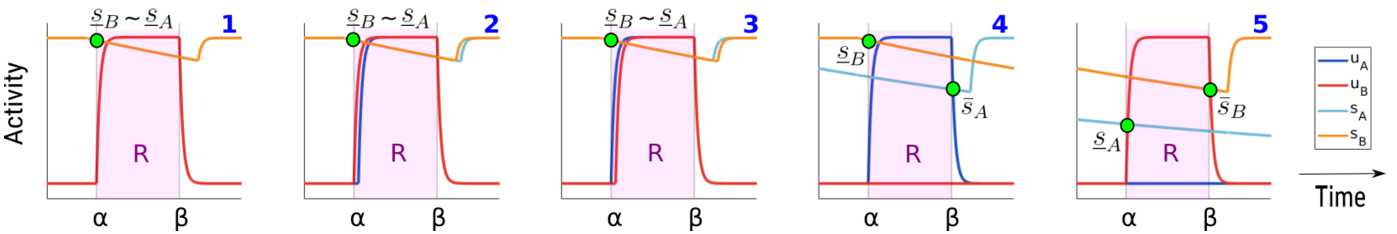
Example dynamics of a MAIN state satisfying conditions $M_{1-5}$ in $R \in \Phi $

Figure 8 shows the time histories of the MAIN states satisfying conditions $M_{1-5}$ in an interval $R \in \Phi $ ($M_{6}$ has been omitted since both units are inactive). This analysis proves that for a fixed interval $R \in \Phi $, any MAIN state of system (1) satisfies only one of conditions $M_{1-6}$, and that any pair of MAIN states satisfying the same condition follows the same dynamics in *R* and leads to the following definition.

#### Definition 6.2

(MAIN classification)

We define the set of MAIN states in $R \in \Phi $ as $M_{R} = \{ s=s(t) \text{ solutions of (1) satisfying one of conditions } M_{1-6} \text{ in } R \} $

An alternative way to visualize the dynamics of each MAIN state is to construct a binary matrix representation (see the next theorem). This tool will enable us to define the existence conditions for $2TR$-periodic states and to rule out impossible ones.

#### Theorem 6

*Let* $R \in \Phi $. *There is an injective map*

$$ \rho ^{R} \colon M_{R} \rightarrow B(2,2) , \qquad s \mapsto V = \begin{bmatrix} x_{A} & y_{A} \\ x_{B} & y_{B} \end{bmatrix} $$

*with entries defined by*

9 $$ \begin{gathered} x_{A}= H\bigl(f(\underline{s}_{B})\bigr), \qquad x_{B}= H\bigl(g(\underline{s}_{A})\bigr), \\ y_{A}= \textstyle\begin{cases} 1 & \textit{if } ax_{B}+f(\underline{s}_{B}) \geq \theta , \\ 0 & \textit{if } ax_{B}+f(\bar{s}_{B}) < \theta , \end{cases}\displaystyle \qquad y_{B}= \textstyle\begin{cases} 1 & \textit{if } ax_{A}+g(\underline{s}_{A}) \geq \theta , \\ 0 & \textit{if } ax_{A}+g(\bar{s}_{A}) < \theta . \end{cases}\displaystyle \end{gathered} $$

*Moreover*,

10 $$ \mathrm{Im}\bigl(\rho ^{R}\bigr) = \Omega \stackrel{\mathrm{def}}{=} \bigl\{ V=\rho ^{R}(s) : x_{A} \leq y_{A}, x_{B} \leq y_{B}, x_{A}=x_{B}=0 \Rightarrow y_{A}=y_{B}=0 \bigr\} . $$

#### Proof

A necessary condition for $\rho ^{R}$ to be well defined is that $y_{A}$ and $y_{B}$ cannot be simultaneously equal to 0 and 1 (i.e. that both inequalities in their definition are not simultaneously satisfied). Due to the decay of the delayed synaptic variables in *R* (Lemma 4), we have $\underline{s}_{B}\geq \bar{s}_{B}$. Moreover, since *f* and *g* are monotonically increasing, we have

11 $$ f(\underline{s}_{B})\leq f(\bar{s}_{B}) \quad \text{and} \quad g( \underline{s}_{B})\leq g(\bar{s}_{B}), $$

which proves that $y_{A}$ is exclusively equal to 0 or 1 (analogously for $y_{B}$).

Next, we notice that any matrix $V=\rho ^{R}(s)$ satisfies the following:

12 $$ x_{A} \leq y_{A}, \qquad x_{B} \leq y_{B}, \qquad x_{A}=x_{B}=0\quad \Rightarrow\quad y_{A}=y_{B}=0. $$

We prove the first inequality $x_{A} \leq y_{A}$ ($x_{B} \leq y_{B}$ is analogous). Without loss of generality, we assume that $x_{A}=1$, and therefore $f(\underline{s}_{B}) \geq \theta $. Since $a\geq 0$ and $x_{B}\geq 0$, we have $ax_{B}+f(\underline{s}_{B}) \geq f(\underline{s}_{B}) \geq \theta $, thus implying $y_{A}=1$. The final part holds because, given $x_{A}=x_{B}=0$, we have $ax_{B}+f(\bar{s}_{B}) \leq f(\underline{s}_{B}) < \theta $ and $ax_{A}+g(\bar{s}_{A}) \leq g(\underline{s}_{A}) < \theta $.

From conditions (11) and (12) it is easily checked that each element $s \in M_{R}$ satisfying condition $M_{i}$ has one of the following images $\rho ^{R}(s)$:

$$ \begin{gathered} (M_{1}) \quad \begin{bmatrix} 1 & 1 \\ 1 & 1 \end{bmatrix} , \qquad (M_{2}) \quad \begin{bmatrix} 1 & 1 \\ 0 & 1 \end{bmatrix} , \qquad (M_{3}) \quad \begin{bmatrix} 0 & 1 \\ 1 & 1 \end{bmatrix} , \\ (M_{4}) \quad \begin{bmatrix} 1 & 1 \\ 0 & 0 \end{bmatrix} , \qquad (M_{5}) \quad \begin{bmatrix} 0 & 0 \\ 1 & 1 \end{bmatrix} , \qquad (M_{6}) \quad \begin{bmatrix} 0 & 0 \\ 0 & 0 \end{bmatrix} . \end{gathered} $$

Since any MAIN state has a distinct image, $\rho ^{R}$ is well defined, injective, and $|Im(\rho ^{R})|=6$. Given that the total number of matrices $V \in B(2,2)$ satisfying conditions (12) are precisely 6 (no other matrix is possible), we have $Im(\rho ^{R})=\Omega $. □

*Classification of CONNECT states.* Our classification and matrix form of CONNECT states follows analogously from that of MAIN states described previously. We recall that in such states, at least one unit turns ON at some time in an active tone interval $R=[\alpha ,\beta ]$. There are three cases to consider:

1. There is $t^{*} \in (\alpha ,\beta ]$ such that unit A (B) turns ON at time *α*, and B (A) turns ON at time $t^{*}$.
2. There is $t^{*} \in (\alpha ,\beta ]$ such that unit A (B) is OFF at time *β*, and B (A) turns ON at time $t^{*}$.
3. There are times $t^{*}, s^{*} \in (\alpha ,\beta ]$ when the A and B units turn ON.

These lead to the conditions in Table 3, which are explained in Supplementary Material 1.6. Case 1 leads to conditions $C_{1-2}$, case 2 leads to conditions $C_{3-4}$, whereas case 3 leads to two possibilities depending on if A turns ON before or after B. For simplicity, we do not distinguish between these possibilities and define ($C_{5}$) as referring to either condition. This leads to the following definition.

**Table 3:** Existence conditions for CONNECT states in an interval $R \in \Phi $

$C_{1}$	$C_{2}$	$C_{3}$	$C_{4}$	$C_{5}^{1}$	$C_{5}^{2}$
$\begin{array}{l} f(\underline{s}_{B})\geq \theta \\ a+g(\underline{s}_{A})<\theta \\ a+g(\bar{s}_{A})\geq \theta \end{array} $	$\begin{array}{l} g(\underline{s}_{A})\geq \theta \\ a+f(\underline{s}_{B})<\theta \\ a+f(\bar{s}_{B})\geq \theta \end{array} $	$\begin{array}{l} g(\underline{s}_{A})<\theta \\ g(\bar{s}_{A})\geq \theta \\ a+f(\bar{s}_{B})<\theta \end{array} $	$\begin{array}{l} f(\underline{s}_{B})<\theta \\ f(\bar{s}_{B})\geq \theta \\ a+g(\bar{s}_{A})<\theta \end{array} $	$\begin{array}{l} t^{*}\leq s^{*} \\ f(\underline{s}_{B})<\theta \\ f(\bar{s}_{B})\geq \theta \\ a+g(\bar{s}_{B})\geq \theta \end{array} $	$\begin{array}{l} t^{*} > s^{*} \\ g(\underline{s}_{A})<\theta \\ g(\bar{s}_{A})\geq \theta \\ a+f(\bar{s}_{A})\geq \theta \end{array} $

#### Definition 6.3

(CONNECT classification)

We define the set of CONNECT states in $R \in \Phi $ as $C_{R} = \{ s=s(t) \text{ solutions of (1) satisfying one of conditions } C_{1-5} \text{ in } R \} $.

Similar to MAIN states, the existence conditions for each CONNECT state in *R* can equivalently be expressed using a binary matrix $W \in B(2,3)$. Indeed, in Supplementary Material 1.7, we prove a version of Theorem 6 valid for $2TR$-periodic CONNECT states. In particular, we prove that for any interval $R \in \Phi $, there exists a well-defined map $\varphi ^{R}\colon C_{R} \rightarrow B(2,3)$ such that each state satisfying one of conditions $C_{1-5}$ has the corresponding image $\varphi ^{R}(s)$ shown below.

$$ \begin{gathered} (C_{1}) \quad \begin{bmatrix} 1 & 1 & 1 \\ 0 & 0 & 1 \end{bmatrix} , \qquad (C_{2}) \quad \begin{bmatrix} 0 & 0 & 1 \\ 1 & 1 & 1 \end{bmatrix} , \qquad (C_{3}) \quad \begin{bmatrix} 0 & 0 & 0 \\ 0 & 0 & 1 \end{bmatrix} , \\ (C_{4}) \quad \begin{bmatrix} 0 & 0 & 1 \\ 0 & 0 & 0 \end{bmatrix} , \qquad (C_{5}) \quad \begin{bmatrix} 0 & 0 & 1 \\ 0 & 0 & 1 \end{bmatrix} . \end{gathered} $$

This analysis naturally leads to the definition of the matrix form of the MAIN and CONNECT states in each interval $R \in \Phi $.

#### Definition 6.4

(Matrix form)

Let $R \in \Phi $ be an active tone interval.

- The matrix form of a MAIN state $s \in M_{R}$ is $V=\rho ^{R}(s)$ defined by (9).
- The matrix form of a CONNECT state $s \in C_{R}$ is $W=\varphi ^{R}(s)$ defined in Supplementary Material 1.7.

#### Remark 6.2

(Visualisation via the matrix form)

The first (second) row of the matrix form of each MAIN state allows us to visualise its A (B) units’ dynamics in *R*. Indeed, given *δ* as defined in Sect. 4.2, we may subdivide *R* into $R=[\alpha ,\alpha +\delta ] \cup [\alpha +\delta ,\beta ] $. The dynamics of unit A at time *α* is given by $x_{A}$. If $x_{A}=1$, then unit A turns ON at time *α* and remains ON in $(\alpha ,\beta ]$. If $x_{A}=0$ and $y_{A}=1$, then unit A is OFF at time *α*, turns ON at time $\alpha +\delta $ and remains ON in $(\alpha +\delta ,\beta ]$. If $y_{A}=0$ (which implies $x_{A}=0$), then unit A is OFF for all $t \in R$. Analogous considerations hold for unit B. A similar approach for visualising the units’ dynamics from the matrix form can be used also for CONNECT states (see Supplementary Material 1.8).

So far we studied the dynamics during an active tone interval *R*. The lemma in Supplementary Material 1.9 proves that a state is LONG if and only if it satisfies two conditions outside this interval. This enables us to provide existence conditions for SHORT and LONG states described in the next section.

## 2TR-periodic states

In this section, we extend the analysis of the previous sections to study $2TR$-periodic states under the conditions $D>TD$ and $TD+D< TR$. We analytically derive parameter conditions leading to the existence of all $2TR$-periodic states in the system and use the matrix form to rule out which states cannot exist.

### Definition 7.1

A state $\psi = \psi (t) = (u_{A}(t),u_{B}(t),s_{A}(t),s_{B}(t))$ is $2TR$-periodic if $\psi (t+2TR) = \psi (t)$ for all $t \in \mathbb{R}$. We call *SM* and *LM* (*SC* and *LC*) the sets of $2TR$-periodic MAIN (CONNECT) states of the SHORT and LONG types, respectively.

Before analysing these states, it is important to first assess the model’s symmetry.

### Remark 7.1

($\mathbb{Z}_{2}$ symmetry)

System (1) is symmetric under the transformation swapping the A and B indexes and by applying the time shift *TR* to the functions $i_{A}$ and $i_{B}$. Indeed, let us rewrite system (1) as a general non-autonomous dynamical system

$$ \dot{v}(t)=z\bigl(v(t),i_{A}(t),i_{B}(t)\bigr), \qquad v=(u_{A},u_{B},s_{A},s_{B}) $$

Now consider the map *κ* whose action swaps the A and B indices of all variables,

$$ \kappa : v=(u_{A},u_{B},s_{A},s_{B},i_{A},i_{B}) \mapsto (u_{B},u_{A},s_{B},s_{A},i_{B},i_{A}). $$

Since $i_{A}(t+TR)=i_{B}(t)$ and $i_{B}(t+TR)=i_{A}(t)$ for all $t \in \mathbb{R}$, we have

$$ \kappa \bigl(z\bigl(v(t),i_{A}(t),i_{B}(t)\bigr)\bigr) = z \bigl(\kappa \bigl(v(t+TR),i_{B}(t +TR),i_{A}(t+TR)\bigr) \bigr), $$

which proves that the model is symmetric under the transformation *κ* time shifted by *TR*. Since no symmetric transformation other than *κ* and the identity exist, the system is $\mathbb{Z}_{2}$-equivariant. Thus, given a periodic solution $v(t)$ with period *T*, its *κ*-conjugate cycle $\kappa (v(t+TR))$ must also be a solution with equal period (asymmetric cycle), except in the case that $v(t)=\kappa (v(t))$ for all $t \in [0,T]$ (symmetric cycle). Asymmetric cycles always exist in pairs, the cycle and its conjugate. We note that in-phase and anti-phase limit cycles with period $2TR$ are both symmetric cycles.

To study *TR*-periodic states, we can replace the set of active tone intervals *I* with

$$ I=I_{1} \cup I_{2}=[0,TD] \cup [TR,TR+TD]. $$

As shown in the previous section, for any state $\psi \in SM$, the activities of both units during each interval $I_{i}$, $i=1,2$, can be represented by a matrix $V_{i}$. This matrix uniquely depends on the values of the delayed synaptic variables at times $\alpha _{i}=(i-1)TR$ and $\beta _{i}=(i-1)TR+TD$. More precisely, in equations (9), we must substitute $\underline{s}_{A}$ with $s^{i-}_{A}$, $\bar{s}_{A}$ with $s^{i+}_{A}$, $\underline{s}_{B}$ with $s^{i-}_{B}$ and $\bar{s}_{B}$ with $s^{i+}_{B}$, where

13 $$ s^{i-}_{A}=s_{A}(\alpha _{i}-D), \qquad s^{i-}_{B}=s_{B}( \alpha _{i}-D), \qquad s^{i+}_{A}=s_{A}(\beta _{i}-D), \qquad s^{i+}_{B}=s_{B}(\beta _{i}-D). $$

### SHORT MAIN states

It turns out (see Theorem 7) that for SHORT MAIN and CONNECT states, these values depend on the following quantities:

14 $$ \begin{gathered} N^{-} = e^{-(TR-TD-D)/\tau _{i}}, \qquad N^{+} = e^{-(TR-D)/ \tau _{i}}, \\ M^{-} = e^{-(2TR-TD-D)/\tau _{i}}, \qquad M^{+} = e^{-(2TR-D)/\tau _{i}}. \end{gathered} $$

Note that $N^{-} \geq N^{+} \geq M^{-} \geq M^{+}$. The dependence of the synaptic variables on these quantities is crucial, because it guarantees that the existence conditions shown in Table 2 depend uniquely on the model parameters.

#### Theorem 7

*There is an injective map*

$$ \rho \colon SM \rightarrow B(2,4) , \qquad \psi \mapsto V = \left [\textstyle\begin{array}{@{}c@{\ \ }|@{\ \ }c@{}} V_{1} & V_{2} \end{array}\displaystyle \right ] = \left [\textstyle\begin{array}{@{}c@{\quad }c@{\ \ }|@{\ \ }c@{\quad }c@{}} x_{A}^{1} & y_{A}^{1} & x_{A}^{2} & y_{A}^{2} \\ x_{B}^{1} & y_{B}^{1} & x_{B}^{2} & y_{B}^{2} \end{array}\displaystyle \right ] , $$

*where* $V_{1}$ ($V_{2}$) *is the matrix form of* *ψ* *in* $I_{1}$ ($I_{2}$) *defined by equations* (9), *and*

15 $$ s_{B}^{i \pm }=N^{\pm }y_{B}^{j}+M^{\pm } \bigl(1-y_{B}^{j}\bigr)y_{B}^{i}, \qquad s_{A}^{i \pm }=N^{\pm }y_{A}^{j}+M^{\pm } \bigl(1-y_{A}^{j}\bigr)y_{A}^{i}, \quad \forall j=1,2, j\neq i. $$

*In addition*,

$$ Im(\rho )=\Omega \stackrel{\mathrm{def}}{=}\left \{V = \left [\textstyle\begin{array}{@{}c@{\ \ }|@{\ \ }c@{}} V_{1} & V_{2} \end{array}\displaystyle \right ] : V_{1} \in Im\bigl(\rho ^{I_{1}}\bigr), V_{2} \in Im\bigl(\rho ^{I_{1}}\bigr) \textit{ satisfying 1--4 below} \right \}, $$

1. $y_{A}^{1}=y_{B}^{2}=1 \Rightarrow x_{A}^{1}=x_{B}^{2}$ *and* $y_{A}^{2}=y_{B}^{1}=1 \Rightarrow x_{A}^{2}=x_{B}^{1}$;
2. $y_{B}^{1}=y_{B}^{2} \Rightarrow x_{A}^{1} \geq x_{A}^{2}$ *and* $y_{A}^{1}=y_{A}^{2} \Rightarrow x_{B}^{2} \geq x_{B}^{1}$;
3. $y_{A}^{2}=1 \Rightarrow x_{B}^{1} \leq r$ *and* $y_{B}^{1}=1 \Rightarrow x_{A}^{2} \leq r$ *for any entry* *r* *in* *V*;
4. $y_{A}^{2}=y_{B}^{2}$, $y_{A}^{1}=y_{B}^{1} \Rightarrow x_{A}^{1} \geq x_{B}^{1}$ *and* $x_{B}^{2} \geq x_{A}^{2}$.

#### Proof

The proofs of equations (15) and conditions 1–4 are given in Supplementary Material 1.10. These conditions imply $Im(\rho ) \subseteq \Omega $. In the next paragraph, we will prove that $Im(\rho )=\Omega $. Assume for now this to be true. The definition of the entries of *V* and identities (15) give multiple necessary and sufficient conditions for determining the dynamics of the corresponding MAIN state $\psi = \rho ^{-1}(V)$ in the intervals $I_{1}$ and $I_{2}$, respectively. Due to the model symmetry (Remark 7.1), *V* is the image of either a symmetrical or an asymmetrical state *ψ*. In the latter case, there exists a matrix $V' \in \Omega $ for a state conjugate to *ψ*. We can easily show that $V'$ is simply defined given *V* by swapping the first (second) row of $V_{1}$ with the second (first) row of $V_{2}$. Notably, both *ψ* and $\psi '$, and thus also *V* and $V'$, exist under the same parameter conditions. The second row of Table 4 shows that all matrices $V \in \Omega $ are images of either a symmetrical state or one of two conjugate states and their names (1st row). Given that *I*, *AP* and *ID* are the only symmetrical cycles (in-phase and anti-phase), by Remark 7.1 all other states have other conjugate cycles that exist under the same conditions.

**Table 4:** Matrix form and existence conditions of all $2TR$-periodic SHORT MAIN states. Names (first row) were chosen following our proposed link between states and percepts in auditory streaming (see Sect. 3). Names starting with S correspond to segregation (no unit responds to both tones), I to integration (one unit responds to both tones, the other is inactive or responding to both tones, too) and AS to bistability (one unit responds to both tones, the other unit to every other tone). The letter D corresponds to states for which one unit turns ON with a small delay after the other unit in at least one active tone interval. The letter B corresponds to states for which both units follow the same dynamics

-	*S*	*SB*	*SD*	*AP*	*AS*	*ASD*	*I*	*ID*	*IB*
Matrix	$\begin{array}{l} 1100 \\ 0000 \end{array} $	$\begin{array}{l} 1100 \\ 1100 \end{array} $	$\begin{array}{l} 1100 \\ 0100 \end{array} $	$\begin{array}{l} 1100 \\ 0011 \end{array} $	$\begin{array}{l} 1111 \\ 0011 \end{array} $	$\begin{array}{l} 1101 \\ 0011 \end{array} $	$\begin{array}{l} 1111 \\ 0000 \end{array} $	$\begin{array}{l} 1101 \\ 0111 \end{array} $	$\begin{array}{l} 1111 \\ 1111 \end{array} $
Conditions	$\begin{array}{l} C_{1} < \theta \\ C_{2}^{+} < \theta \\ C_{3}^{+} < \theta \end{array} $	$\begin{array}{l} C_{3}^{+} < \theta \\ C_{8}^{-} \geq \theta \end{array} $	$\begin{array}{l} C_{4}^{-} \geq \theta \\ C_{2}^{-} \geq \theta \\ C_{3}^{+} < \theta \\ C_{8}^{-} < \theta \end{array} $	$\begin{array}{l} C_{2}^{+} < \theta \\ C_{3}^{-} \geq \theta \end{array} $	$\begin{array}{l} C_{3}^{-} \geq \theta \\ C_{5}^{+} < \theta \\ C_{8}^{-} \geq \theta \end{array}$	$\begin{array}{l} C_{2}^{-} \geq \theta \\ C_{3}^{-} \geq \theta \\ C_{5}^{+} < \theta \\ C_{8}^{-} < \theta \end{array} $	$\begin{array}{l} C_{1} \geq \theta \\ C_{6}^{+} < \theta \end{array} $	$\begin{array}{l} C_{3}^{-} \geq \theta \\ C_{5}^{-} \geq \theta \\ C_{7}^{-} < \theta \end{array} $	$C_{7}^{-} \geq \theta $
Short	−	$C_{9}<\theta $	$C_{9}<\theta $	−	$C_{10}<\theta $	$C_{10}<\theta $	−	$C_{10}<\theta $	$C_{10}<\theta $

In the next part, we define the conditions for the existence of each of the states reported in the third row of Table 4, which are equivalent to the well-definedness conditions of the corresponding matrix form $V \in \Omega $. These conditions depend on:

16 $$ \begin{gathered} C_{1} = d, \qquad C_{2}^{\pm } = a-bM^{\pm }+ d, \qquad C_{3}^{\pm } = c-bN^{\pm }, \qquad C_{4}^{\pm } = c-bM^{\pm }, \\ C_{5}^{\pm } = a-bN^{\pm }+d,\qquad C_{6}^{\pm } = a-bN^{\pm }+c, \qquad C_{7}^{\pm } = d- bN^{\pm }, \\ C_{8}^{\pm } = d-bM^{\pm },\qquad C_{9} = a-bM^{+}, \qquad C_{10} =a-bN^{+}. \end{gathered} $$

We determine conditions for the well-definiteness of each matrix $V \in \Omega $ from the definitions of the entries of $V_{1}$ and $V_{2}$ given in (12) and using formulas (15). Notably, all the existence conditions uniquely depend on the system parameters. When determining these conditions, we notice that many of them are redundant and can be simplified using the following properties: $N^{-} \geq N^{+} \geq M^{-} \geq M^{+}$, $d \leq c$ and $a \geq 0$. In the next paragraph, we give one example (*AS*) and leave the remaining for the reader to prove. The names and the sets of inequalities defining each state is reported in the middle row of Table 4. Note that such inequalities are well posed, meaning that there is a region of parameters where they are all satisfied. This effectively proves that for each matrix $V \in \Omega $, there exists a state $\psi =\rho ^{-1}(V) \in SM$ whose dynamics during intervals $I_{1}$ and $I_{2}$ are defined by the entries of *V*.

We now prove that the existence conditions of *AS* are well-defined, that is,

17 $$ V_{AS}= \left [\textstyle\begin{array}{@{}c@{\quad }c@{\ \ }|@{\ \ }c@{\quad }c@{}} 1 & 1 & 1 & 1 \\ 0 & 0 & 1 & 1 \end{array}\displaystyle \right ] \quad \Leftrightarrow \quad C_{3}^{-} \geq \theta , \qquad C_{5}^{+} < \theta , \qquad C_{8}^{-} \geq \theta . $$

From the theorem condition (1) we have that

$$ \begin{gathered} x_{A}^{1}=1 \quad \Rightarrow \quad y_{A}^{1}=1, \qquad x_{A}^{2}=1 \quad \Rightarrow\quad y_{A}^{2}=1, \\ x_{B}^{2}=1 \quad \Rightarrow\quad y_{B}^{2} =1, \qquad y_{B}^{1}=0 \quad \Rightarrow \quad x_{B}^{1}=0. \end{gathered} $$

This obviously leads to the following equivalence:

$$ V_{AS}= \left [\textstyle\begin{array}{@{}c@{\quad }c@{\ \ }|@{\ \ }c@{\quad }c@{}} 1 & 1 & 1 & 1 \\ 0 & 0 & 1 & 1 \end{array}\displaystyle \right ] \quad \Leftrightarrow \quad x_{A}^{1} = 1, \qquad x_{B}^{2} = 1, \qquad x_{A}^{2} = 1, \qquad y_{B}^{1}=0. $$

Using the definition of the entries defined in (12) and the identities for the synaptic quantities given in equations (15), we observe the following:

1. $y_{A}^{1}=1 ( y_{B}^{2}=1 ) \Rightarrow s_{A}^{2-} =N^{-} ( s_{B}^{1-}=N^{-} ) $, which implies $x_{B}^{2}=x_{A}^{1}=H(c-bN^{-})$.
2. $y_{B}^{1}=0 \text{ and } y_{B}^{2}=1 \Rightarrow s_{B}^{2-}= M^{-}$. From this we have $x_{A}^{2}=H(d-bM^{-})$.
3. $y_{A}^{2}=1 \Rightarrow s_{A}^{1+}=N^{+}$. This and $y_{B}^{1}=0$ give $y_{B}^{1}=H(a+d-bN^{+})$.

Overall, from cases (1)–(3) we obtain

$$ \begin{gathered} x_{A}^{1} = 1, \qquad x_{B}^{2} = 1 \quad \Leftrightarrow \quad C_{3}^{-} \geq \theta , \\ x_{A}^{2} = 1 \quad \Leftrightarrow \quad C_{8}^{-} \geq \theta , \\ y_{B}^{1}=0 \quad \Leftrightarrow\quad C_{5}^{+} < \theta . \end{gathered} $$

This completes the proof for both claim (17) and the theorem. □

#### Remark 7.2

(Conditions $C_{9}$ and $C_{10}$)

The middle row of Table 4 shows the states’ existence conditions in the intervals $I_{1}$ and $I_{2}$. However, they do not guarantee that units A and B are OFF outside these intervals (i.e. being SHORT). Some MAIN SHORT states in Table 4 need additional existence conditions to guarantee them being SHORT. These conditions involve quantities $C_{9}$ and $C_{10}$ and are shown in the bottom row of Table 4. Their proof is in Supplementary Material 1.11.

#### Remark 7.3

(Table 4)

The conditions in the middle and bottom rows of Table 4 complete the existing conditions for all $2TR$-periodic SHORT MAIN states. Indeed these conditions cover all possible matrix forms and corresponding states. The middle row shows conditions determining the dynamics within in the intervals $I_{1}$ and $I_{2}$. The bottom row shows conditions that guarantee units to be OFF in $[0,2TR]-I$.

**Figure 9:**
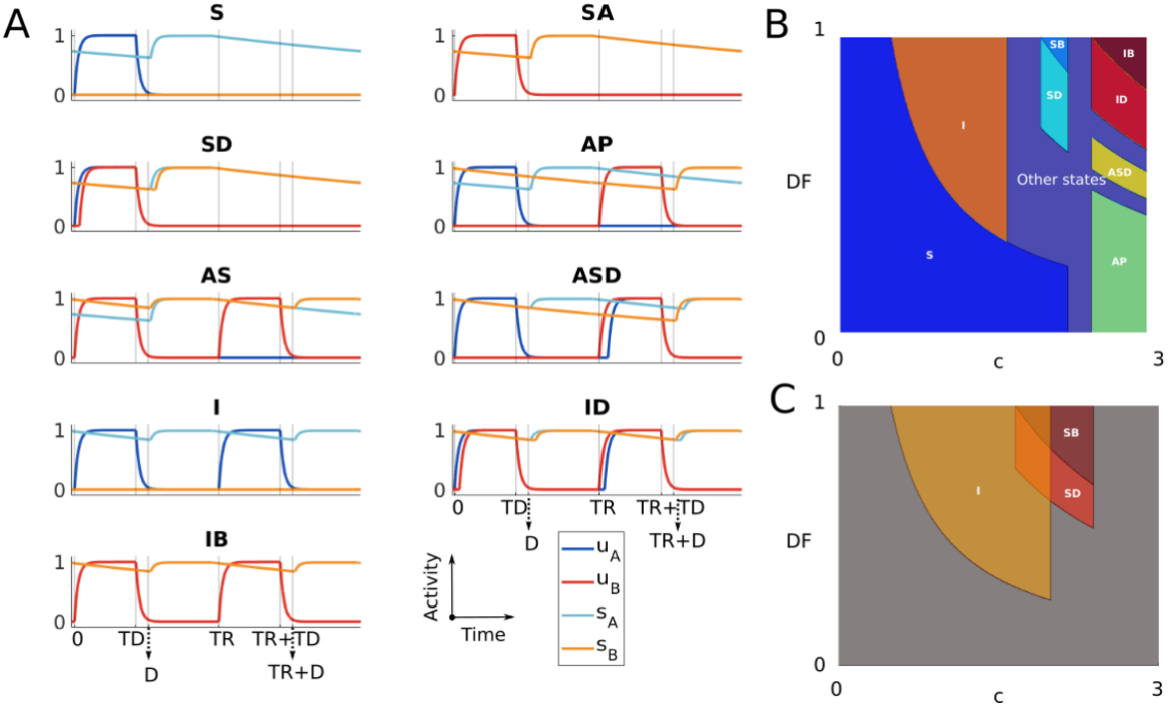
(**A**) Time histories of $2TR$-periodic SHORT MAIN states. (**B**) Existence regions of states in A when varying *DF* and *c*. (**C**) Existence regions for states *I*, *SB* and *SD* when varying *c* and *DF*. Parameters in (**B**) and (**C**) are $\tau _{i}=0.4$, $\theta =0.5$. The remaining parameters in (**B**) are $TD = 0.03$, $D = 0.03$ $PR=17$, $a = 0.6$, $b = 2$, while parameters in (**C**) are $TD = 0.005$, $D = 0.015$, $PR=5$, $a = 0.4$, $b = 3$

Figure 9A shows the time histories for each $2TR$-periodic SHORT MAIN state in Table 4. Note that the conditions given in this table allow us to determine the regions where each of these states exists in the parameter space. To visualize two-dimensional existence regions when varying pairs of parameters, we defined a new parameter $DF \in [0,1]$ and set $d=cDF$ (*DF* is a scaling factor for the inputs from tonotopic locations). Figure 9B shows the two-dimensional region of existence of each of these states at varying *DF* and input strength *c*. Note that we can visualise the existence regions by varying any parameters in the system.

The multistability theorem in Supplementary Material 1.12 uses the conditions in Table 4 to prove that only the pair of $2TR$-periodic SHORT MAIN states $(I,SB)$ and $(I,SD)$ may coexist in the parameter space. Figure 9C shows a parameter regime in which state *I* coexists with *SB* and *SD*.

### Remaining states

As shown in the Sect. 6.1, $2TR$-periodic states can be SHORT MAIN (*SM*), SHORT CONNECT (*SC*), LONG MAIN (*LM*) or LONG CONNECT (*LC*) during each interval $I_{1}$ and $I_{2}$. We denote by $X|Y$ the set of states satisfying condition *X* during $I_{1}$ and *Y* during $I_{2}$, where $X,Y \in \{ SM,SC,LM,LC \}$. In Sect. 7.1, we analysed the existence conditions of $SM|SM$ states. We extended a similar analysis for all remaining combinations of states. We define a matrix form to rule out impossible states and to find the existence conditions for existing states. The study of SHORT CONNECT ($SC|SM$, $SM|SC$ and $SC|SC$) and LONG MAIN ($LM|LM$, $SM|LM$ and $LM|SM$) states are in Supplementary Material 1.13 and 1.14, respectively. All remaining combinations of sets $X|Y$ are analysed in Supplementary Material 1.15, and they conclude the existence conditions for all $2TR$-periodic states. Overall we find 41 different MAIN and CONNECT states (excluding conjugate states). Their existence conditions can be visualized as a 2D parameter projection, similar to Fig. 9B for SHORT MAIN states. Supplementary Material 1.16 shows an example for SHORT CONNECT and LONG MAIN states.

## Biologically relevant case: $2TR$-periodic states for $D\leq TD$

In this section, we study model states and their link to auditory streaming under $D\leq TD$ and $TD+D< TR$. These inequalities are relevant to studying auditory streaming. The first inequality is valid for short delays, which are likely generated by delayed synaptic inhibition. The second inequality is guaranteed for the values of *TD* and *TR* typically tested in these experiments (further motivated in Discussion).

**Figure 10:**
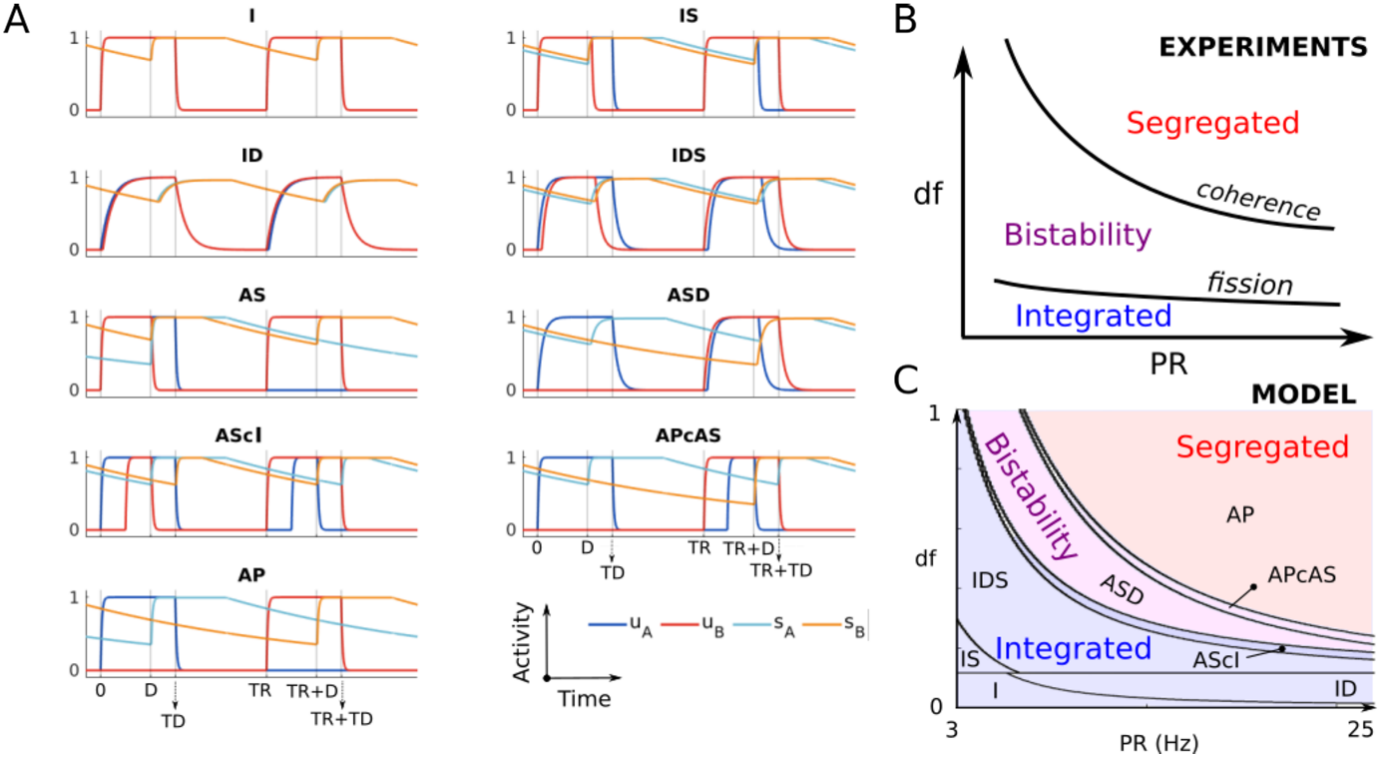
(**A**) Time histories of all $2TR$-periodic states for $D< TD$ and $TD+D< TR$. (**B**) Schematic diagram of the experimentally measured perceptual regions when varying *PR* and *df*. (**C**) Existence regions of the states in (**A**) at varying *PR* and *df*. States corresponding to integration, segregation or bistability are grouped by background colours (see Remark 8.2). Model parameters in (**C**) are $\tau _{i}=0.2$, $\theta =0.5$, $TD=0.03$, $D=0.01$, $c=5$, $a=1$, $b=2$ and $m=6$

Using a similar approach of the previous section, we analytically derive the conditions for the existence of all possible $2TR$-periodic states. Overall, we find 10 possible states. We link these states with the possible perceptual outcomes in the auditory streaming paradigm and find a qualitative agreement between the model and experiments when varying input parameters *df* and *PR* (Figs. 10B and C). We derive the coherence and fission boundaries as functions of *PR* using the states’ existence conditions (equations (20)).

We now proceed to analyze $2TR$-periodic states by considering active tone intervals $I=I_{1} \cup I_{2}$, where $I_{1}=[0,TD]$ and $I_{2}=[TR,TR+TD]$. We assume that tonotopic inputs to the units are stronger than their mutual inhibition, that is,

18 $$ c-b\geq \theta , $$

a condition that allows unit A (B) to turn and remain ON at each A (B) active tone interval $I_{1}$ ($I_{2}$). Indeed, from the model equations (1)–(2), the total input to the A unit is $au_{B}-bs_{B}(t-D)+c\geq c-b$ for all $t\in I_{1}$. Thus on the fast time scale, the A unit turns ON instantaneously at the start of $I_{1}$ and remains ON for all $t \in I_{1}$. For analogous reasons, the B unit is ON throughout $I_{2}$. This has two important consequences:

1. The synaptic variables $s_{A}(t-D)$ and $s_{B}(t-D)$ are constant and equal to 1 in $[D,TD+D]$ and $[TR+D,TR+TD+D]$, respectively. This implies that the total inputs to the B and A units in these intervals are equal to $a-b+d$.
2. Both units are OFF for all $t \in \mathbb{R}-I$ (i.e. no LONG states can exist). Indeed, from point 1 above $s_{A}(t-D)$ ($s_{B}(t -D)$) is equal to 1 at time *TD* ($TR+TD$), and thus the total input to the B (A) unit at this time is $a-b$, which is less than *θ* due to hypothesis ($U _{1}$). Thus the B (A) unit turns OFF instantaneously at time *TD* ($TR+TD$), and it is followed by A (B) due to Sect. 4.1. Since $(0,0)$ is an equilibrium for the fast subsystem with no input (see Sect. 4.3), we conclude that both units are OFF until the next active tone input.

From point 1 the input to the B (A) unit in $[D,TD+D]$ ($[TR +D,TR+TD+D]$) is equal to $P=a-b+d$. This and point 2 imply that B and A can turn ON only in the intervals $L_{1}=[0,D]$ and $L_{2}=[TR,TR+D]$, respectively. We consider two cases.

### Case $P\geq \theta $

This case leads to the two states shown in Table 5. Indeed, since unit B is ON in $I_{2}$, unit A is ON in this interval, because its total input is $a-bs_{A}(t-D)+d\geq P\geq \theta $. This is true also for unit B in $I_{1}$. Moreover, both units turn OFF instantaneously at times *TD* and $TR+TD$ (see point 2 above). Thus units evolve equally on each active tone interval (on the fast time scale). The only difference is that B (A) may turn ON with small delay $\delta \sim \tau $ after A (B) in $I_{1}$ ($I_{B}$). When evaluated at time 0 (*TR*), the delayed variable $s_{A}$ ($s_{B}$) is equal to $N^{-}$. Due to the model symmetry, there are only two possible states, *I* and *ID*. For *I*, both units turn ON at the same time 0 and *TR* for $d-bN^{-}\geq \theta $ ($C_{7}^{-}\geq \theta $). If $d-bN^{-}<\theta $, then we have the state *ID* for which B (A) turns ON with small delay *δ* after A (B) in $I_{1}$ ($I_{2}$).

**Table 5:** MAIN states existence conditions for $D< TD$ and $TD+D< TR$ and $P\geq \theta $

*I*	*ID*
$\begin{array}{l} C_{7}^{-} \geq \theta \\ P \geq \theta \end{array} $	$\begin{array}{l} C_{7}^{-} < \theta \\ P \geq \theta \end{array} $

### Case $P<\theta $

Under this condition, unit B (A) is OFF in $[D,TD]$ ($[TR+D,T D]$) and outside the active tone intervals. The dynamics of units B and A in intervals $L_{1}$ and $L_{2}$, respectively, is yet to be determined. Lemma 2 proves that the delayed synaptic variables are monotonically decaying in these intervals. We can use the classification of MAIN and LONG states presented in Sects. 6.1 by replacing interval *I* with *L*, where $L=L_{1}$ or $L=L_{2}$. We fix $L=L_{1}$ ($L=L_{2}$). Since unit A (B) is ON in *L* due to condition (18), MAIN states in *L* can satisfy only conditions $M_{1}$, $M_{2}$ and $M_{4}$ ($M_{1}$, $M_{3}$ and $M_{5}$). Similarly, CONNECT states in *L* can satisfy only condition $C_{1}$ ($C_{2}$). The matrix form of MAIN states can be extended to a $2 \times 3$ binary matrix (see Supplementary Material 1.13). Moreover, since A (B) is ON in $L_{1}$ ($L_{2}$) due to condition (18), the matrix form of any $2TR$-periodic MAIN and CONNECT state is

$$ \left [\textstyle\begin{array}{@{}c@{\quad }c@{\quad }c@{\ \ }|@{\ \ }c@{\quad }c@{\quad }c@{}} 1 & 1 & 1 & x_{A}^{2} & y_{A}^{2} & z_{A}^{2} \\ x_{B}^{1} & y_{B}^{1} & z_{B}^{1} & 1 & 1 & 1 \end{array}\displaystyle \right ] . $$

The synaptic quantities defining the entries of the matrix form in $L_{1}$ and $L_{2}$ are

19 $$ \begin{gathered} s_{A}^{2 \pm }=s_{B}^{1 \pm }=N^{\pm }, \\ s_{A}^{1 \pm }= \textstyle\begin{cases} R^{\pm } & \text{if } z_{A}^{2}=1, \\ M^{\pm } & \text{otherwise,} \end{cases}\displaystyle \quad \text{and} \quad s_{B}^{2 \pm }= \textstyle\begin{cases} R^{\pm } & \text{if } z_{B}^{1}=1, \\ M^{\pm } & \text{otherwise,} \end{cases}\displaystyle \end{gathered} $$

where $R^{-}=e^{-(TR-2D)/\tau _{i}}$ and $R^{+}=e^{-(TR-D)/\tau _{i}}$. The quantities $M^{\pm }$ and $N^{\pm }$ are defined in equations (13). The proof of these identities is in Supplementary Material 1.17. By applying identities (19) to the definition of the entries of the matrix form of MAIN or CONNECT states we obtain that $z_{A}^{2} = z_{B}^{1} \Rightarrow x_{A}^{2} = x_{B}^{1} \text{ and } y_{A}^{2} = y_{B}^{1} $.

This condition reduces the total number of combinations of binary matrices (and relative MAIN and CONNECT states) to those shown in Table 6. The first five states in this table are MAIN, and the last two are CONNECT and complete the set of all possible states. Using identities (19) on the definition of the entries in each state’s matrix form and applying simplifications imply the existence conditions shown in the bottom row of Table 6, where $R_{6}^{-} = a-bR^{-}+d$ and $R_{7}^{-} = d-bR^{-} $.

**Table 6:** Matrix forms of MAIN/CONNECT states for $D< TD$, $TD+D< TR$ and $P\geq \theta $. Asymmetrical states in *. The names of CONNECT states contain the letter c and the name of the two MAIN states separated by the CONNECT state in the parameter space (see Fig. 10)

*IS*	*IDS*	$AS^{*}$	$ASD^{*}$	*AP*	$APcAS^{*}$	*AScI*
$\begin{array}{l} 111 | 111 \\ 111 | 111 \end{array}$	$\begin{array}{l} 111 | 011 \\ 011 | 111 \end{array}$	$\begin{array}{l} 111 | 000 \\ 111 | 111 \end{array}$	$\begin{array}{l} 111 | 000 \\ 011 | 111 \end{array}$	$\begin{array}{l} 111 | 000 \\ 000 | 111 \end{array}$	$\begin{array}{l} 111 | 000 \\ 001 | 111 \end{array}$	$\begin{array}{l} 111 | 001 \\ 001 | 111 \end{array}$
$\begin{array}{l} R_{7}^{-} \geq \theta \\ P < \theta \end{array} $	$\begin{array}{l} R_{7}^{-} < \theta \\ R_{6}^{-} \geq \theta \\ P < \theta \end{array} $	$\begin{array}{l} C_{5}^{+} < \theta \\ C_{8}^{-} \geq \theta \end{array} $	$\begin{array}{l} C_{5}^{+} < \theta \\ C_{8}^{-} < \theta \\ C_{2}^{-} \geq \theta \end{array} $	$C_{2}^{+} < \theta $	$\begin{array}{l} C_{2}^{-} < \theta \\ C_{2}^{+} \geq \theta \end{array} $	$\begin{array}{l} R_{6}^{-} < \theta \\ C_{5}^{+} \geq \theta \end{array} $

Figure 10A shows the time histories for the states presented in Tables 5 and 6. Since unit A(B) must be ON during the A(B) tone interval for property (18), there are no possible other network states. A proof similar to the multistability theorem in Supplementary Material 1.12 shows that any pair of these states cannot coexist.

#### Remark 8.1

(Extension to the case $TD+D\geq TR$)

The condition $TD+D< TR$ enabled us to obtain a complete classification of network states via the application of Lemma 2. However, these states can exist also if $TD+D\geq TR$ with few adjustments in their existence conditions (see Supplementary Material 1.18). We note that under this condition, other $2TR$-periodic states exist, such as states where both units turn ON and OFF multiple times during each active tone interval (not shown). Since the condition $TD+D\geq TR$ is met for high values of *PR* for which $TR \sim TD$, we explored this condition using computational tools (see Sect. 9).

### Model states and link with auditory streaming

We now show how states described in the previous section can explain the emergence of different percepts during auditory streaming. In the following framework, each possible percept is linked (↔) with the units’ activities in the corresponding state:

- Integration ↔ both units respond to all tones (*I*, *ID*, *IS*, $IDS$ and $AScI$).
- Segregation ↔ no unit respond to both tones (*AP*).
- Bistability ↔ one unit responds to both tones, and the other to only one tone (*AS*, $ASD$ and $APcAS$). This interpretation is motivated further in Remark 8.2.

Thus all model states presented in the previous section belong to one perceptual class. The cartoon in Fig. 10B shows the experimentally detected regions of parameters *df* and *PR* where participants are more likely to perceive integration, segregation or bistability (van Noorden diagram; see Introduction). We now validate our proposed framework of rhythm tracking by comparing model states consistent with different perceptual interpretations (percepts) in the $(df,PR)$-plane. In these tests the model parameter *d* is scaled by *df* as in Sect. 3. Figure 10C shows regions of the existence of model states when fixing all other parameters (as reported in the caption). States classified as integration, segregation and bistability are grouped by blue, red and purple background colours to facilitate the comparison with Fig. 10B. The existence regions of states corresponding to integration and segregation qualitatively match the perceptual organization in the van Noorden diagram.

*Computation of the fission and coherence boundaries.* Our analytical approach enables us to formulate the coherence and fission boundaries as functions of *PR* using the states’ existence conditions. More precisely, the coherence boundary is the curve $df_{\mathrm{coh}}(PR)$ separating states $APcAS$ and *AP*, whereas the fission boundary is the curve $df_{\mathrm{fiss}}(PR)$ separating states $AScI$ and $IDS$:

20 $$ \begin{gathered} df_{\mathrm{coh}}(PR) = \bigl[ \bigl(a-bN^{+}+c-\theta \bigr)/c\bigr]^{m} , \\ df_{\mathrm{fiss}}(PR) = \bigl[\bigl(a-bM^{+}+c-\theta \bigr)/c \bigr]^{m}, \end{gathered} $$

where $N^{+}=e^{-(TR-D)/\tau _{i}}$ and $M^{+}=e^{-(2TR-TD)/\tau _{i}}$. The existence boundaries in Fig. 10C (including these curves) naturally emerge from the model properties and are robust to parameter perturbations. For example, the parameters *a* and *b* can respectively shift and stretch the two curves $df_{\mathrm{coh}}(PR)$ and $df_{\mathrm{fiss}}(PR)$. For all parameter combinations, these curves have an exponential decay in *TR* that generates regions of existence similar to the van Noorden diagram.

#### Remark 8.2

The model predicts the emergence of integration, segregation and bistability in plausible regions of the parameter space. Yet, it currently cannot explain (1) how perception can switch between these two interpretations for fixed *df* and *PR* values (i.e. perceptual bistability) and (2) which of the two tone streams is followed during segregation (i.e. A-A- or -B-B). This could be resolved in a competition network model, such as that proposed by [17]. The selection of which rhythm is being followed by listeners at a specific moment in time would be resolved by a mutually exclusive selection of either unit: the perception is either integration if a unit responding to both tones is selected or segregation if a unit responding to every other tone is selected (see Discussion).

#### Remark 8.3

(A note on the word bistability)

Bistability (as used in Fig. 10C) corresponds to states that encode both integrated and segregated rhythms simultaneously, where one unit responds to both tones, and the other to one tone (say, unit A responds ABAB…, and unit B responds -B-B…). This should not be confounded with the fact that this bistable state coexists with another, by our definition, bistable state (unit A responds A-A-…, and unit B responds ABAB…).

## Computational analysis with smooth gain and inputs

In this section, we extend the analytical results using numerical simulations with a continuous rather than the Heaviside gain function and inputs and reducing the timescale separation ratio $\tau _{i}/\tau $ by an order of magnitude. We restrict our study to $D< TD$ (the biologically realistic case), but without imposing the condition $TD+D< TR$. This allows us to make predictions at high $PR\text{ s}$, which go beyond the analytic predictions of the previous section (see Remark 8.1). In summary, we find that this smooth, non-slow-fast regime generates similar states occupying slightly perturbed regions of stability. We consider the sigmoidal gain function $S(x)=[1+\exp (-\lambda x)]^{-1}$ with fixed slope $\lambda =30$, and we consider continuous inputs from equations (3).

**Figure 11:**
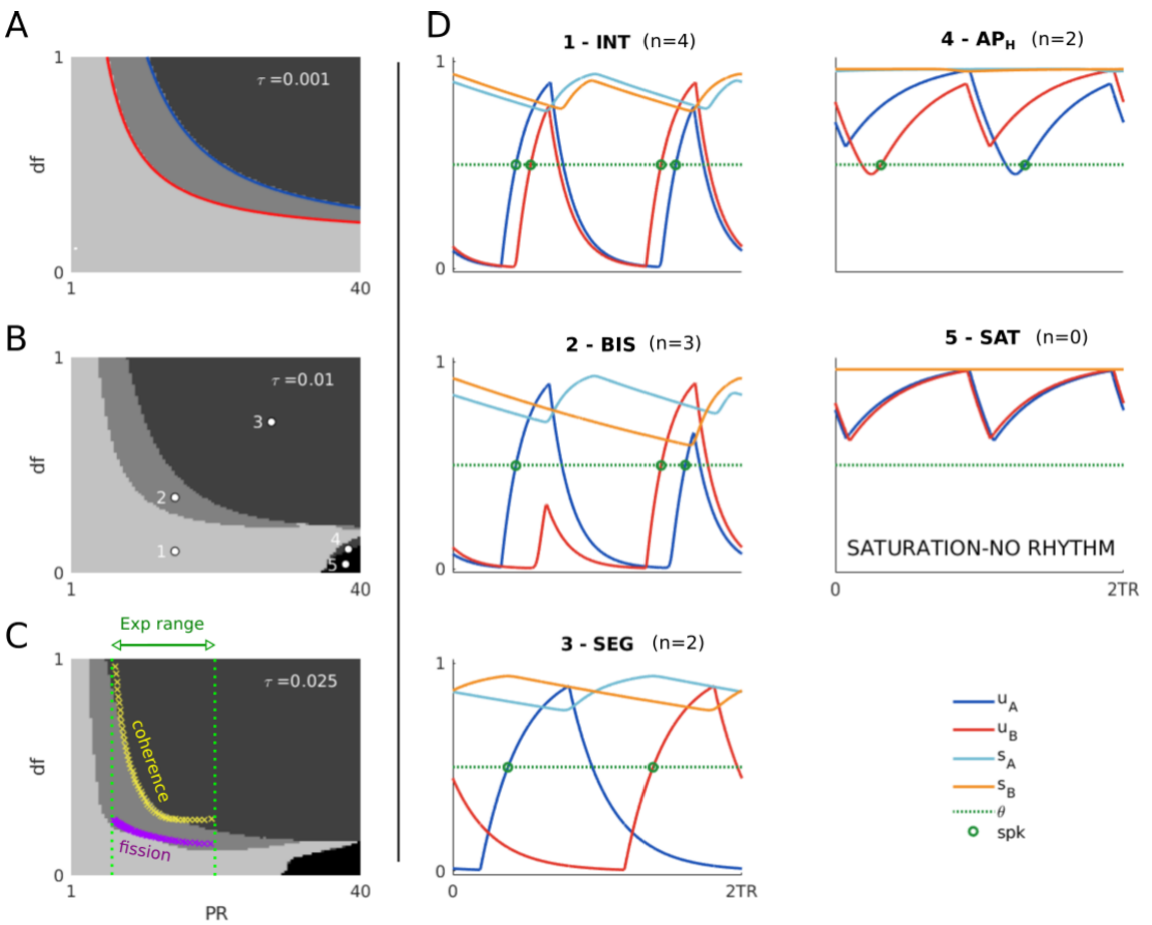
(**A**)–(**C**) show the numbers of threshold crossings for both units *n* in greyscale for simulated trajectories at varying *PR* and *df* (uniformly sampled in 96 points) for different values of *τ* shown in top-right corner of each panel. Black corresponds to $n=0$, and the lightest gray to $n=4$. In (**A**) the blue and red curves are the analytically predicted coherence and fission boundaries defined in equations (20). In (**C**), yellow and purple crosses represent respectively the experimentally detected coherence and fission boundaries, replotted from Fig. 2 in [47]. We plotted regions outside the experimental range *PR* in 5–20 Hz for predictions. (**D**) Time histories for the model states in each of the five regions of panel (**B**), with values of *PR* and *df* shown by white dots in panel (**B**). All parameters are as in Fig. 3

Integration (INT), segregation (SEG) and bistability (BIS) are classified based on the number of threshold crossings during one periodic interval $[0,2TR]$. Let $n_{A}$ ($n_{B}$) denote the number of threshold crossings of unit A (B), and let $n=n_{A}+n_{B}$. Based on the correspondence between states and perception described in the previous section, the states for which $n=4$ ($n=2$) correspond to integration (segregation), and the states for which $n=3$ correspond to bistability. We run large parallel simulations to systematically study the convergence to the $2TR$-periodic states under changes in *df* and *PR* and detect the boundaries of transitions between different perceptual interpretations. We consider a grid of $l \times l$ uniformly spaced parameters $PR \in [1,40]\text{ Hz}$ and $df\in [0,1]$ ($l=98$). For each node, we run long simulations from the same initial conditions and compute the number of threshold crossings after the convergence to a stable $2TR$-periodic state for different values of *τ* (Figs. 11A, B and C). There are five possible regions corresponding to one of four different values of $n \in \{0,2,3,4\}$. Three of these regions (as in panel A) correspond to the three coloured regions found analytically in Fig. 10C. Figure 11D shows example time histories of all the states in these five regions when $\tau =0.01$ (the values of *PR* and *df* are shown in white dots in panel B).

For low values of *τ* (panel A), the system is in the slow-fast regime. The blue and red curves show the analytically predicted coherence and fission boundaries for the Heaviside case under slow-fast regime defined in equations (20). These curves closely match the numerically predicted boundaries in the smooth system. For panels B and C, the parameter *τ* is increased. All the existing states found in panel A persist and occupy the largest region of the parameter space, but the fission and coherence boundaries perturb. Note that the selected values of *D* and *TD* in these figures lead to the condition $TD+D\geq TR$ for $PRs$ greater than approximately 27 Hz, where the following two new $2TR$-periodic states appear:

$AP_{H}$ ($n=2$). Both units oscillate at activity levels higher than the threshold ∼*θ*. Since $n=2$, this state may correspond to segregation, but its perceptual relevance is difficult to assess, because it occurs in a small region of the parameter space and at high $PRs$, which is outside the range tested in psychoacoustic experiments.

$SAT$ ($n=0$). Both units’ activities are higher than the threshold *θ* (saturation). This state exists at (a) low $dfs$ and (b) high $PRs$, greater than 30 Hz. Property (a) guarantees that inputs are strong enough to turn ON both units, whereas property (b) guarantees that that successive active tone intervals occur rapidly compared to the decay *τ* of the units’ activities. High values *τ* preclude the units from turning OFF and the crossing of the threshold *θ*. This state does not correspond to any auditory streaming percept (integration or segregation). However, *PR* typically ranges between 5 and 20 Hz in these experiments. This state may explain why perceivable isochronal rhythms above $\sim 30\text{ Hz}$ are heard as a pure tone in the first (lowest) octave of human hearing. Indeed, when $df=0$, the model inputs represent the repetition of a single tone (B = A) with frequency *PR*. Our proposed framework suggests that $SAT$ cannot track any rhythm simply because no unit crosses threshold.

The coherence and fission boundaries detected from the network simulations in panel Fig. 11C quantitatively match those from psychoacoustic experiments (yellow and purple crosses, the available data spans $PRs$ in $\sim [7,20]\text{ Hz}$). The model parameters chosen in the this figure (including *τ*) have been manually tuned to match the data. Overall, we conclude that the proposed modelling framework is a good candidate for explaining the perceptual organisation in the van Noorden diagram and for perceiving repeated tones (isochronal rhythms) at high frequencies as a single pure tone in the lowest octave of human hearing.

## Discussion

We proposed a minimal firing rate model encoding ambiguous rhythm perception consisting of two neural populations coupled by fast direct excitation and slow delayed inhibition and forced by square-wave periodic inputs. By acting on different timescales excitation and inhibition give rise to rich dynamics studied in this paper.

The model incorporates neural mechanisms commonly found in auditory cortex (ACx). We hypothesised that pitch and rhythm are respectively encoded in tonotopic primary and secondary ACx [11]. Model units represent populations in secondary ACx, that is, belt or parabelt regions of auditory cortex, with inputs that mimic primary ACx responses [49] to interleaved A and B tone sequences [14]. This division of roles in the ACx is supported by evidence for specific non-primary belt and parabelt regions encoding temporal features (i.e. rhythmicity) only present in sound envelope rather stimulus features (i.e. content like pitch) as in primary ACx [11]. The timescale separation between excitation and inhibition is consistent with AMPA and GABA synapses, respectively (widely found in cortex).

The inhibition, with delay assumed fixed to *D*, could be determined by factors including slower inhibitory activation times (vs excitatory), indirect connections via interneurons and propagation times between the spatially separated A and B populations. A recent computational study addressed the role of the two inhibitory populations of parvalbumin-(PV) and somatostatin-(SST) positive interneurons and an excitatory (EXC) population in the ACx [50]. In their model the responses of SST interneurons (but not PV interneurons) to pure tones show a delayed response after PV and EXC and motivates the inhibitory delays assumed in our model. The modelled units and timescale separation considered in our work would encode the action of the delayed inhibitory SST and fast EXC populations, but not PV. Another experimental result in the same paper shows that SST inactivation decreases forward masking at best frequency sites. This is consistent with our results, where forward masking decreases following a reduction in the inhibitory strengths, which are in turn modulated by the level of the units’ activity.

We used analytical tools to investigate periodic solutions 1:1 locked to the inputs and their dependence on key parameters influencing auditory perception: the presentation rate (*PR*), the tones’ pitch difference (*df*) and the tone duration (*TD*). For these analytical results, we assumed the condition $TD+D<1/PR$, which enabled us to classify all possible states and formulate existence conditions and rule out impossible states. This condition is relevant to auditory streaming. Indeed, the factors that may play a role in generating delayed inhibition discussed above would most likely lead to short or moderate delays, for which this condition is guaranteed for the values of $PR\text{ s}$ and *TD*s typically considered in experiments, *PR* in 5–20 Hz and *TD* in 10–30 ms (*TD*’s interpretation discussed below in Predictions). We used numerical simulations to study the case $TD+D\geq 1/PR$ and to extend the confirm the validity of the analytical approach with a smooth gain function, smooth inputs and different levels of timescale separation. The simulations closely matched the analytical predictions under the slow-fast regime. Reducing the timescale separation shifts the regions of existence of the perceptually relevant states and produces a qualitatively close match with the van Noorden diagram.

We proposed a link between states and the rhythms perceived during auditory streaming based on threshold crossing of the units’ responses: for ABAB integrated percepts, both units respond to every tone, and for segregated A-A- or -B-B percepts, each unit responds to only one tone. Bistability corresponds to one unit responding to every tone and the other unit responding to every other tone. This interpretation of bistability can explain how both integrated and segregated rhythms may be perceived simultaneously, as reported in some behavioural studies [51, 52], but not the dynamic alternation between these two percepts [17, 53] (see the section “Future work”). This classification enabled us to compare the states’ existence regions to those of the corresponding percepts when varying *df* and *PR* in experiments (van Noorden diagram). A qualitatively similar organization of these regions emerged naturally from the model and is robust to parameter perturbations.

### Models of neural competition

Our proposed model addresses the formation of percepts but not switching between them, the so-called auditory perceptual bistability [17, 53]. Future work will consider the present description acts as a front-end to a competition network, which could be the locus of attention [54] (we can think of the present study as a reformulation of the pre-competition stages in [17]). Perceptual bistability (e.g. binocular rivalry) is the focus of many theoretical studies [22–24] that feature mechanisms and dynamical states similar to those reported here with two key distinctions. Firstly, our model units are associated with tonotopic locations of the A and B tones, not with percepts as in many other models. Secondly, previous firing rate models typically considered a combination of fixed inputs, instantaneous mutual inhibition and a slow processes such as adaptation or synaptic depression that drives perceptual switches. Periodic inputs associated with slow switches in specific experimental paradigms have been considered in several models [28, 29, 42, 55, 56]. Mechanisms of adaptation and synaptic depression have not been considered in the present model because we aim to explain the formation of perceivable rhythms at the pre-competition stage, not the perceptual bistability. Indeed, slow adaptation might feature at higher stage of the model (see Conclusions).

### Models of auditory streaming

The auditory streaming paradigm has been the focus of a wealth of electrophysiological and imaging studies in recent decades. However, it has received far less attention from modelers when compared with visual paradigms. Many existing models of auditory streaming have used signal-processing frameworks without a link to neural computations (recent reviews: [13, 15, 16]). In contrast, our model is based on a plausible network architecture with biophysically constrained and meaningful parameters. Our model is a departure from (purely) feature-based models because it incorporates a combination of mechanisms acting at timescales close to the interval between tones. By contrast, [47] considers neural dynamics only on a fast time scale (less than TR). Further, [17] considers slow adaptation to drive perceptual alternations, assumes instantaneous inhibition and slow NMDA-excitation, a combination that precludes forward masking as reported in [14]. The entrainment of intrinsic oscillations to inputs was considered in [18], albeit using a highly redundant spatio-temporal array of oscillators. Recently, a parsimonious neural oscillator framework was considered in [19] but without addressing how the same percepts persist over a wide range of *PR* (5–20 Hz).

A central hypothesis for our model is that network states associated with different perceptual interpretations are generated before entering into competition that produces perceptual bistability (as put forward in [57] with a purely algorithmic implementation). Here network states are emergent from a combination of neural mechanisms: mutual fast, direct excitation and mutual slow acting, delayed inhibition. In contrast with [17], our model is sensitive to the temporal structure of the stimulus present in our stereotypical description of inputs to the model from primary auditory cortex and over the full range of stimulus presentation rates.

A popular conceptual model for explaining the perceptual dependence on *df* and *PR* is the population separation hypothesis (PSH) [45]. According to this hypothesis, A and B tones evoke spatially organised tonotopic responses spreading to neighbouring sites, with a peak at the A and B frequency locations (A and B populations) and overlapped activity in between (so-called X population). The reported primary ACx recordings [45] show that increasing *PR* suppresses overall response amplitudes, whereas increasing *df* reduces the overlap in the activity evoked by the tones, eventually leading to no overlap at large *df*. Therefore, at large *df*, two tone streams would activate either the A or the B population every other tone (segregation). At small *df*, there is a large response in populations A, B and X, reflecting a response to every tone as a model (integration). At intermediate *df* the dominant percept varies in *PR*. At low $PR\text{ s}$ the population X responds to both tones and leads to integration, whereas at large $PR\text{ s}$ the suppression of the responses leads to segregation.

Our modelling proposal follows the PSH hypothesis by considering tonotopically localized A and B units with lateral inputs to mimic the influences from overlapping responses, yet without modelling an intermediate X unit directly. States linked to integration and segregation produce activity at every tone and at every other tone, respectively, like the corresponding states in the PSH. States linked to bistability have overlapping A and B units’ activities at every other tone, resembling the activation of an intermediate X population in the PSH. Unlike the PSH, our model can explain the emergence of integration at low *PR* and high *df* (see below).

### Predictions

In van Noorden’s original work on auditory streaming, boundaries in the $(df,PR)$-plane were identified: the temporal coherence boundary, below which only integrated occurs, and the fission boundary, above which only segregated occurs. We derived exact expressions for these behavioural boundaries that match the van Noorden diagram. One of challenges in developing a model that reproduces the van Noorden diagram was explaining how a neural network can produce an integrated-like state at very large *df*-values and low *PR*s. Primary ACx shows no tonotopic overlap in this parameter range (A-location neurons exclusively respond to A tones) [14]. Our results show that fast excitation can make this possible. Disrupting AMPA excitation is predicted to preclude the integrated state at large *df*-values. Furthermore, our results show that segregation relies on slow acting, delayed inhibition, which performs forward masking. Whilst the locus for this GABA-like inhibition cannot yet be specified, we predict that its disruption would promote the integrated percept.

Some model parameters (i.e. *TD*, *TR*, input strengths) can readily be tested in experiments by changing sound inputs. The model can predict the effect of such changes on perception. However, the role of *TD* has yet to be investigated in experiments. In our model, *TD* better represents the duration of the primary ACx responses to tones, rather than the sound duration of each tone. This interpretation is supported by recordings of firing rates at tonotopic locations in Macaque primary ACx [14]. In these data, $\sim 80 \%$ of the response is localized shortly after the tone onset. This time window is approximately constant $\sim 30\text{ ms}$ across different tone intervals, tone durations, *PR* and *df* (unpublished results).

Numerics for the smooth model predict a region at large $PR\text{ s}$ for which responses are saturated (no threshold crossings). These responses are consistent with rapidly repeating discrete sound events at rates above 30 Hz sounding like a low-frequency tone (20 Hz is typically quoted as the lowest frequency for human hearing). At presentation rates above 30 Hz, we predict a transition from hearing a modulated low-frequency tone to hearing two fast segregated streams as *df* is increased.

### Conclusions

Our study proposed that sequences of tones are perceived as integrated or segregated through a combination of feature-based and temporal mechanisms. Here the tone frequency is incorporated via input-strengths, and timing mechanisms are introduced via excitatory and inhibitory interactions at different timescales including delays. We suspect that the proposed architecture is not unique in being able to produce similar dynamic states and the van Noorden diagram. The implementation of globally excitatory inputs ($i_{A}(t)$ and $i_{B}(t)$ driving both units) rather than mutual fast-excitation is expected to produce similar results.

The resolution of competition between these states is not considered at present. Imaging studies implicate a network of brain areas (e.g. frontal and parietal) extending beyond auditory cortex for streaming [58–61], some of which are generally implicated in perceptual bistability [62–64]. The model could be extended to consider perceptual competition and bistability by incorporating further downstream into a competition stage (in the same spirit as [17]). An extended framework would provide the ideal setting to explore perceptual entrainment through the periodic [65] or stochastic [66] modulation of a parameter like *df*.

## Supplementary Information

Below is the link to the electronic supplementary material.

Supplementary information (PDF 573 kB)

## Data Availability

Source code to reproduce the results presented are available on a public GitHub repository at https://github.com/ferrarioa5/ferrario_rankin2021.git.

## Abbreviations

ACx: auditory Cortex
*TD*: tone duration
*TR*: tone repetition time
*PR*: presentation rate
