# Supplementary material for: Auditory streaming emerges from fast excitation and slow delayed inhibition
Source: J Math Neurosci. 2021 May 3;11:8. doi: 10.1186/s13408-021-00106-2 (PMC8093365; doi:10.1186/s13408-021-00106-2)
Supplement: Supplementary file 1 — Supplementary information (PDF 573 kB) [file 13408_2021_106_MOESM1_ESM.pdf]

RESEARCH

Auditory streaming emerges from fast excitation and slow delayed inhibition

Andrea Ferrario* and James Rankin

*Correspondence:
A.A.Ferrario@exeter.co.uk
Department of Mathematics, College of Engineering, Mathematics & Physical Sciences, University of Exeter, Exeter, UK
Full list of author information is available at the end of the article

1 Supplementary Material

1.1 Basin of attraction of equilibria $(1,0)$ and $(0,1)$

In this section we analyze the fast subsystem:

$$\begin{aligned} u_A' &= -u_A + H(au_B - b\tilde{s}_B + c) \\ u_B' &= -u_B + H(au_A - b\tilde{s}_A + d) \end{aligned} \tag{1}$$

From the inequalities given in Table 1 in the main text we note that points $(1,0)$ and $(0,1)$ cannot coexist with any other equilibrium and thus have trivial basins of attraction. However, $(0,0)$ and $(1,1)$ may coexist under the following conditions:

$$\begin{aligned} b\tilde{s}_B + \theta - a &\leq c < b\tilde{s}_B + \theta \\ b\tilde{s}_A + \theta - a &\leq d < b\tilde{s}_A + \theta \end{aligned} \tag{2}$$

Thus we must have $a > 0$, i.e. when the excitation is not absent in the model. To study the basin of attraction for these two equilibria, we consider the vector field of system 1. For convenience we introduce the following quantities: $s_1 = (b\tilde{s}_A - c + \theta)/a$ and $s_2 = (b\tilde{s}_B - c + \theta)/a$. Conditions 2 hold if and only if $0 < s_k \leq 1$, for $k = 1, 2$. Thus we can rewrite system 1 as:

$$\begin{aligned} u_A' &= -u_A + H(a(u_B - s_2)) \\ u_B' &= -u_B + H(a(u_A - s_1)) \end{aligned} \tag{3}$$

Since H is the Heaviside function a can be removed. Figure 1 (left) shows an example basins of attraction for parameter values for which $(0,0)$ and $(1,1)$ coexist (black circles). The u_A- and u_B-nullclines are shown in blue and red, respectively. We simulated model 3 starting from several initial conditions, covering the phase space. Simulated trajectories converge either to $(0,0)$ (green) and $(1,1)$ (purple) and show the subdivision in the basin of attraction.

There is a degenerate fixed point (s_1, s_2) (red dot), where separatrices (yellow and orange lines) originate, dividing the phase plane into the regions of attraction. These curves are given by (proved below):

$$\begin{cases} (u_A - 1)s_2/(s_1 - 1) & \text{if } u_A \leq s_1 \\ u_A(s_2 - 1)/s_1 + 1. & \text{otherwise} \end{cases}$$

We prove that these curves define the separatrices by showing the convergence of orbits from initial conditions (u_A^0, u_B^0) in the top left corner in Figure 1 (left) to $(1, 1)$ (purple trajectories). A similar proof holds for initial conditions in other regions of the phase-space and for convergence to $(0, 0)$. Points (u_A^0, u_B^0) in the top left corner belong to the set:

$$\Omega_L = \{(u_A, u_B) : u_A < s_1 \text{ and } u_B > (u_A - 1)s_2/(s_1 - 1)\}$$

Since $\Omega_L \subset [0, u_A] \times [u_B, 1]$, system 3 becomes:

$$\begin{aligned} u_A' &= 1 - u_A \\ u_B' &= -u_B \end{aligned}$$

Consider an orbit starting from $(u_A^0, u_B^0) \in \Omega_L$. Since $u_A' > 0$ the orbit will move towards the right until it reaches the vertical line $u_A = s_1$. The trajectory follows the same equations at all times t, since:

$$u_B(t) = u_B^0 \frac{u_A - 1}{u_A^0 - 1} > s_2 \frac{u_A - 1}{s_1 - 1} > s_2$$

Where the last inequality holds because $s_1 > u_A$. Thus, any trajectory ends on the top-right corner defined by:

$$\Omega_R = \{(u_A, u_B) : u_A \geq s_1 \text{ and } u_B \geq s_2\}$$

After the orbit reaches the curve $u_A = s_1$, $(u_A, u_B) \in \Omega_R$ it follows the system:

$$\begin{aligned} u_A' &= 1 - u_A \\ u_B' &= 1 - u_B \end{aligned}$$

Since $u_A' > 0$ and $u_B' > 0$ the trajectory continues to satisfy these equations and will converge to $(1, 1)$ (both turn ON simultaneously).

Similar equilibria, separatrices and basin of attractions occur with continuous (steep) sigmoidal gains. This analysis was carried out using numerical continuation techniques. We therefore replace the Heaviside function H with a Sigmoid gain function with threshold 0 and slope λ for parameter values for which points $(0, 0)$ and $(1, 1)$ coexist and compare with the results presented in Remark 3.1 for the Heaviside gain. We consider the following system:

$$\begin{aligned} u_A' &= -u_A + S(a(u_B - s_2)) \\ u_B' &= -u_B + S(a(u_A - s_1)) \end{aligned} \tag{4}$$

Parameter a acts as a multiplicative factor on the slope λ. Figure 1 shows qualitatively similar phase portrait and the basins of attraction between the case with the Heaviside and Sigmoid gains (slope $\lambda = 20$ and $a = 1$). The stable equilibrium points $(0, 0)$ and $(1, 1)$ (black circles), the u_A- and u_B-nullclines (blue and red) and the saddle-separatrices (yellow and orange curves) discussed in Remark 3.1 for the

Heaviside case persist and are slightly shift in the Sigmoid case. Furthermore, the degenerate (s_1, s_2) saddle for the Heaviside case becomes a standard saddle point and slightly deviates from (s_1, s_2) (red circles). The equilibia for the Sigmoidal case were detected numerically with Newton's method. Saddle separatrices (yellow and orange curves) were also found numerically via backward integration from an initial point near the saddle, in the unstable direction of the eigenvector.

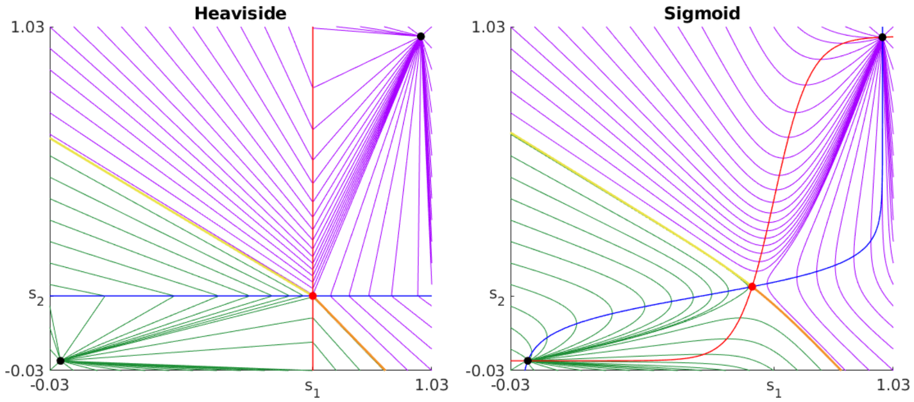

Figure 1 Phase portrait and basin of attraction for system 3 with $s_1 = 0.7$ and $s_2 = 0.4$ with gain function given by a Heaviside (left) or a Sigmoid with slope $\lambda = 20$ and $a = 1$ (right). The left panel is redrawn from Figure 1 in the main text. Purple and green lines show orbits converge to $(1, 1)$ and $(0, 0)$, respectively in the Heaviside case, or to equilibria $\sim (1, 1)$ and $\sim (0, 0)$ in the Sidmoid case (black circles). The u_A- and u_B-nullclines are shown in blue and red, respectively. Yellow and orange lines show the saddle-separatrices of the point (s_1, s_2) (red circle). Point (s_1, s_2) is a degenerate saddle for the Heaviside case and a standard saddle for the Sidmoid case.

1.2 Fast dynamics in the absence of inputs

Theorem 1 (dynamics in $\mathbb{R}-I$) *For any $t \in \mathbb{R}-I$:*

1 *If A or B is OFF at time t, both units are OFF in $(t, t^*]$, where*

$$t^* = \min_{s \in I}\{s > t\}$$

2 *If A or B is ON at time t, both units are ON in $[t_*, t)$, where*

$$t_* = \max_{s \in I}\{s < t\}$$

Proof We begin by proving 1. Due to Section 4.3 in the main text the fast subsystem 1 with no inputs $(c = d = 0)$ has only two possible equilibria at any time in $[t_*, t^*]$: $P = (0, 0)$ and $Q = (1, 1)$. At time t_*, if Q is not an equilibium or (u_A, u_B) is in the basin of attraction of P the system instantaneously converges to P (i.e. both units are/turn OFF). Since P is an equilibium at any time in $\mathbb{R}-I$ the units remain OFF throughout $[t_*, t^*] \subset \mathbb{R}-I$, which proves the theorem. Next, assume that Q is also an equilibium and that (u_A, u_B) instantaneously converges to Q at time t_* (i.e. both units are/turn ON at time t^*). By hypothesis of point 1. one unit is OFF at time t. By continuity there must be a turning OFF time in $\tilde{t} \in [t_*, t)$. This can occur only if Q is not an equilibrium at time $\tilde{t}$, due to the dynamics of the slow

variables. Thus since P is an equilibrium at any time in $\mathbb{R}-I$ both units turn OFF at time $\tilde{t}$ and remain OFF in $(t, t^*] \subset [\tilde{t}, t^*]$. This concludes the proof of 1.

We prove 2. by contradiction. Suppose there $\exists \bar{t} \in [t_*, t)$ when one unit is OFF. From 1. we have both units OFF in $(\bar{t}, t^*]$. This is absurd given that one unit is ON at time $t \in (\bar{t}, t^*]$. □

1.3 Synaptic decay lemma

Lemma 2 (synaptic decay) *If TD+$D < TR$ the delayed synaptic variables $s_A(t$–$D)$ and $s_B(t-D)$ are monotonically decreasing in $[\alpha_k^A, \alpha_k^A + D]$ or $[\alpha_k^B, \alpha_k^B + D]$, $\forall k \in \mathbb{N}$*

Proof This lemma is illustrated in Figure 6A . From Remark 4.1 in the main text the synaptic variable s_A (s_B) is monotonically decreasing except for when A (B) turns ON. Due to Theorem 1 in the main text such an event cannot occur at any time $t \in \mathbb{R}-I$. Thus, it is sufficient to prove that $t-D \in \mathbb{R}-I$. Without loss of generality (WLOG) consider $L = [2kTR, 2kTR+D]$ and $t \in L$, which implies: $2kTR-D \leq t-D \leq 2kTR$. To complete the proof, the condition $TD+D < TR$ implies:

$$2kTR-D \geq (2(k-1)+1)TR+TD = \beta_{k-1}^B \implies \beta_{k-1}^B \leq t-D \leq \alpha_k^A$$

The last inequalities imply $t-D \in \mathbb{R}-I$ and conclude the proof. □

1.4 No saturation lemma

Lemma 3 (no saturated states) *If TD+D<TR both units are OFF in the intervals $(\alpha_k^A + TD + D, \alpha_k^B]$ and $(\alpha_k^B + TD + D, \alpha_{k+1}^A]$, $\forall k \in \mathbb{N}$.*

Proof We prove the theorem for the interval $(\alpha_k^A + TD + D, \alpha_k^B]$ (extension to other intervals is analogous). By contradiction suppose $\exists \bar{t}$ in this interval when either unit, say A, is ON. Since $TD+D < TR$ we have $\bar{t} \in \mathbb{R}-I$. Theorem 1 in the main text implies both units are ON in $[t_*, \bar{t})$, where $t_* = \alpha_k^A + TD$. Thus, at time $p_* = t_*$+$D \in [t_*, \bar{t})$ the delayed synaptic variables tends to 1 following the fast system (equations (4) in the main text) . From this and condition U_1 in Remark 2.1 in the main text we have that $a-bs_A(p_*-D) \sim a-b < \theta$ and $a-bs_B(p_*-D) \sim a-b < \theta$. Hence $(0, 0)$ must be the only stable equilibrium at time p^*, which is absurd since BOTH units are ON at this time. □

1.5 Single OFF to ON transition Lemma

Here we prove the following Lemma, that derives from Lemma 2 and Lemma 3.

Lemma 4 (single OFF to ON transition) *Let $D > TD$ and $TD+D < TR$ and consider an active tone interval $R = [\alpha, \beta] \in \Phi$. Let A (B) be ON at a time $\bar{t} \in R$, then*

(1) A (B) is ON $\forall t \geq \bar{t}$, $t \in R$

(2) $\exists!\, t_A^*$ (t_B^*) $\in R$ when A (B) turns ON

(3) $s_A(t-D)$ ($s_B(t-D)$) is decreasing for $t \in [\alpha, t_A^* + D]$ ($t \in [\alpha, t_B^* + D]$)

Proof We prove this Lemma for the A unit and for the interval $R = I_k^A$, i.e. we assume that $\alpha = \alpha_k^A$ and $\beta = \beta_k^A$, where $R = [\alpha, \beta]$. The extension to the other intervals and for the B unit is analogous. Let us call $\gamma = \gamma_k^A$. Since $TD + D < TR$ we can apply Lemma 2, which implies $s_A(t-D)$ and $s_B(t-D)$ to be monotonically decreasing in $[\alpha, \gamma]$. Moreover, since $D > TD$ we have that $R \subseteq [\alpha, \gamma]$. Thus the delayed synaptic variables are monotonically decreasing in R.
We now prove (1). On the fast time scale (u_A, u_B) follow the fast subsystem 1 at time $\bar{t}$ and may converge to one of the four equilibria described in Table 1 in the main text. However, since A is ON at time $\bar{t}$ trajectories converge to either $(1, 0)$ or $(1, 1)$.

In the first case (convergence to $(1, 0)$) we have

$$c \geq bs_B(\bar{t}-D) + \theta.$$

Due to the decay of the synaptic variables, the same inequality holds $\forall t \geq \bar{t} \in R$. This condition is guaranteed only for the two equilibrium points $(1, 0)$ and $(1, 1)$. Therefore any orbit either remains fixed at $(1, 0)$ or undergo a transition to $(1, 1)$.

In the second case (convergence to $(1, 1)$) we have

$$a+c \geq bs_B(\bar{t}-D)+\theta$$

$$a+d \geq bs_A(\bar{t}-D)+\theta.$$

Due to the decay of the synaptic variables these inequalities hold $\forall t \geq \bar{t} \in R$. Therefore $(1, 1)$ remains an equilibrium at such times. In both cases (convergence to $(1, 0)$ or $(1, 1)$) the A unit is ON $\forall t \geq \bar{t} \in R$, proving (1).

We now prove (2). Lemma 3 implies that A is OFF for some $t < \alpha$. Suppose that A is ON at time $\bar{t}$. For continuity, there $\exists t_A^* \in R$ when the A unit undergoes an OFF to ON transition, thus proving the first claim. The uniqueness of t_A^* follows by contradiction. Suppose the existence of two distinct OFF to ON transition times p^*, $q^* \in R$ for the A unit. We can assume that $p^* < q^*$. Since A turns ON at time q^*, there $\exists r^* \in R$ with $p^* < r^* < q^*$ such that A is OFF at time r^*. The fact that A turns ON at time p^* and is OFF at time $r^* > p^*$ contradicts (1).

Lastly we prove (3) for $s_A(t-D)$. Since $[\alpha, t_A^* + D]$ is the union of closed intervals R and $[\beta, t_A^* + D]$, proving that $s_A(t-D)$ is monotonically decreasing in each of these subintervals would suffice. We previously proved that $s_A(t-D)$ is monotonically decreasing in R. Thus, we are left to prove that the same property holds in $[\beta, t_A^* + D]$.

Due to Remark 4.1 in the main text we have to prove that A cannot turn ON at any time in the interval $[\beta-D, t_A^*]$. Due to point (2) of the current lemma the turning ON time t_A^* for A exists and is unique in the interval R. Therefore A does not turn ON in $[\alpha, t_A^*]$. Moreover since $D < TR$ we have $\beta - D = \beta_k^A - D > \alpha_{k-1}^B$, which leads to $[\beta-D, \alpha] \subset \mathbb{R} - I$. From Theorem 1 in the main text we have that A cannot turn ON in $[\beta-D, \alpha]$. Thus we have that A cannot turn ON in $[\beta-D, \alpha] \cup [\alpha, t_A^*] = [\beta-D, t_A^*]$, which yields the desired result. $\square$

1.6 Classification of CONNECT states

To define a classification and matrix form for CONNECT states we consider the following cases:

- **A(B) turns ON at time α and B(A) turns ON at time t^*,** $\exists t^* \in (\alpha, \beta]$. These two conditions are equivalent to $(1,0)$ $((0,1))$ and $(1,1)$ being equilibria for the the fast subsystem at time α and β, respectively. We note that the validity of the previous statement is due to $(1,0)$ being in the basin of attraction of $(1,1)$ for any set of parameters (as shown in Figure 1). There are two conditions for which this occurs:
  (C_1) $f(\underline{s}_B) \geq \theta$, $a+g(\underline{s}_A) < \theta$ and $a+g(\bar{s}_A) \geq \theta$
  (C_2) $g(\underline{s}_A) \geq \theta$, $a+f(\underline{s}_B) < \theta$ and $a+f(\bar{s}_B) \geq \theta$
  C_1 (C_2) describes the case where the B (A) units turn ON within the interval R and the A (B) unit is ON at time α.
- **A(B) is OFF at time β and B(A) turns ON at time t^*,** $\exists t^* \in (\alpha, \beta]$. These two events correspond to $(0,0)$ and $(0,1)$ $((1,0))$ being equilibria for the the fast subsystem at time α and β, respectively. The following conditions lead to the following cases:
  (C_3) $g(\underline{s}_A) < \theta$, $g(\bar{s}_A) \geq \theta$ and $a+f(\bar{s}_B) < \theta$
  (C_4) $f(\underline{s}_B) < \theta$, $f(\bar{s}_B) \geq \theta$ and $a+g(\bar{s}_A) < \theta$
  C_3 (C_4) describes the case where the A (B) units is OFF at time β and the B (A) turns ON within R.
- $\exists t^*, s^* \in (\alpha, \beta]$ **times when the A and B unit turns ON**. The conditions leading to this case are different depending on if A turns ON before or after B, that is:
  1 A turns ON before B - if $t^* \leq s^*$, $f(\underline{s}_B) < \theta$, $f(\bar{s}_B) \geq \theta$ and $a+g(\bar{s}_B) \geq \theta$
  2 B turns ON before A - if $t^* > s^*$, $g(\underline{s}_A) < \theta$, $g(\bar{s}_A) \geq \theta$ and $a+f(\bar{s}_A) \geq \theta$
  In both cases, $(0,0)$ and $(1,1)$ are equilibria for the fast subsystem respectively for $t < \min\{t^*, s^*\}$ and $t \geq \max\{t^*, s^*\}$. In the first and second cases respectively $(1,0)$ and $(0,1)$ are equilibria for $t \in [t^*, s^*)$ ($t \in [s^*, t^*)$). For simplicity we decide not to distinguish between the cases 1. and 2. and define (C_5) as referring to either condition.

1.7 CONNECT matrix form

Theorem 5 *Set $R \in \Phi$. There is an injective map:*

$$\varphi^R \colon C_R \to B(2,3)$$
$$s \mapsto W = \begin{bmatrix} x_A & y_A & z_A \\ x_B & y_B & z_B \end{bmatrix}$$

With entries defined by:

$$\begin{aligned} &x_A = H(f(\underline{s}_B)),\ y_A = H(ax_B + f(\underline{s}_B)),\ z_A = H(a + f(\bar{s}_B)) \\ &x_B = H(g(\underline{s}_A)),\ y_B = H(ax_A + g(\underline{s}_A)),\ z_B = H(a + g(\bar{s}_A)) \end{aligned} \tag{5}$$

And we have:

$$Im(\varphi^R) = \Gamma \stackrel{\text{def}}{=} \{W : x_A \leq y_A \leq z_A, x_B \leq y_B \leq z_B, x_A = x_B = 0 \Rightarrow y_A = y_B = 0, y_A < z_A \text{ or } y_B < z_B\}$$

Proof We first prove that the entries of any matrix $W=\varphi^R(s)$ satisfy the three conditions in Γ. It is easy to show that, since $a \geq 0$, $f(\underline{s_A}) \leq f(\bar{s_A})$ and $f(\underline{s_B}) \leq f(\bar{s_B})$ the first conditions, i.e. $x_A \leq y_A \leq z_A$ and $x_B \leq y_B \leq z_B$, hold. The condition $x_A = x_B = 0 \Rightarrow y_A = y_B = 0$ simply follows from identities 5. One can check that any CONNECT state defined by conditions C_i, $\forall i = 1, .., 5$ satisfies $y_A < z_A$ or $y_B < z_B$.

Using $x_A \leq y_A \leq z_A$ and $x_B \leq y_B \leq z_B$ one can easily see that each CONNECT state satisfying one of conditions C_{1-4} has a corresponding image $\varphi^R(s)$ shown below. The case C_5 is treated separately, since both A and B turn ON at times t^* and s^*, respectively.

- If $t^* \leq s^*$ it is clear that $f(s_B(t^*)) = \theta$ and $g(s_A(t^*)) < \theta$. Thus, since s_A and g are respectively decreasing and increasing functions in R, we must have $g(\underline{s}_A) = g(s_A(0)) < g(s_A(t^*)) < \theta$. In addition $a + f(\bar{s}_B) \geq f(\bar{s}_B) \geq \theta$ and $a + g(\bar{s}_B) \geq \theta$.
- If $t^* > s^*$ similar considerations lead to $f(\underline{s}_B) < \theta$. In addition $a + g(\bar{s}_A) \geq g(\bar{s}_A) \geq \theta$ and $a + f(\bar{s}_A) \geq \theta$.

In both cases we thus have $x_A = x_B = 0$ (which leads to $y_A = y_B = 0$) and $z_A = z_B = 1$.

$$(C_1)\begin{bmatrix}1&1&1\\0&0&1\end{bmatrix}\quad(C_2)\begin{bmatrix}0&0&1\\1&1&1\end{bmatrix}\quad(C_3)\begin{bmatrix}0&0&0\\0&0&1\end{bmatrix}\quad(C_4)\begin{bmatrix}0&0&1\\0&0&0\end{bmatrix}\quad(C_5)\begin{bmatrix}0&0&1\\0&0&1\end{bmatrix}$$

Since any CONNECT state has a distinct image, φ^R is well defined and injective. It is trivial to prove that $Im(\varphi^R) \subseteq \Gamma$. However, since $|\Gamma| = 6$, we must have $Im(\varphi^R) = \Gamma$. □

1.8 Visualization of CONNECT states via matrix form

The dynamics of the A (B) unit in R of a CONNECT state is represented by the first (second) row of its matrix form. For example, for the state defined by condition C_2 unit A turns ON at time $t^* \in (\alpha, \beta]$ and unit B turns ON at time α. We may subdivide R into $R = [\alpha, \alpha+\delta] \cup [\alpha+\delta, t^*] \cup [t^*, \beta]$. From conditions C_2 we have $y_A = 0$ (which implies $x_A = 0$) and $z_A = 1$. Thus A is OFF during $[\alpha, \alpha+\delta]$ and $[\alpha+\delta, t^*]$, turns ON at time t^* and remains ON in $[t^*, \beta]$. Since $x_B = 1$ (which implies $y_B = z_B = 1$), the B unit turns ON at time α and remains ON in $[\alpha, \beta]$.

1.9 Proof of the LONG states theorem

The following lemma regards the conditions for which LONG states can occur outside these intervals. The idea of the proof is sketched in Figure 2. Both units of a LONG stats must be ON at time β due to Theorem 1 in the main text, which proves to 2. Furthermore, since both synaptic variables are monotonically decaying in the interval $[\alpha, t^* + D]$ this activity must persist until time $t^* + D$, when one of these variables (or both) jump to 1.

Lemma 6 (LONG conditions) *A state is LONG if and only if* $\exists R = [\alpha, \beta] \in \Phi$ *such that*

1 *A and B turn ON at times* t^*_A *and* $t^*_B \in R$, *respectively.*
2 $a - bs_A(\beta - D) \geq \theta$ *and* $a - bs_B(\beta - D) \geq \theta$.

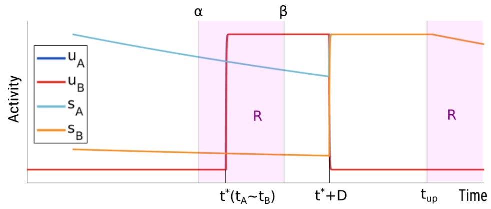

Figure 2 Example dynamics of a LONG state showing quantities used in Lemma 6.

Moreover, both units are ON in $[\beta, t^*+D]$*, turn OFF at time* t^*+D*, and are OFF in* $(t^*+D, t_{up}]$*, where*

$$t^* = \min\{t^*_A, t^*_B\} \quad \textit{and} \quad t_{up} = \min_{s\in I}\{s > t\}.$$

Proof (⇒) Consider a LONG state. By definition one unit is ON at time t, for some $t \geq t_0 \in \mathbb{R} - I$. Thus $t \in T \cup S = (\beta^A_k, \alpha^B_k) \cup (\beta^B_k, \alpha^A_{k+1})$, for some $k \in \mathbb{N}$ (where $T \cap S = \emptyset$). WLOG suppose $t \in T$. We will prove the claim for $R = [\alpha^A_k, \beta^A_k]$. Theorem 1 in the main text implies both units being ON in $[\beta, t)$, where $\beta = \beta^A_k$. The application of Lemma 4 at time $\bar{t} = \beta \in R$ implies the existence of (unique) OFF to ON transition times $t^*_A, t^*_B \in R$ for the A and B units, respectively, which proves point 1. Since both units are ON in $[\beta, t)$ for $t > \beta$, they are ON at time $\beta + h$, for $h > 0$ arbitrarily small. At this time the inputs are OFF ($\beta + h \in \mathbb{R} - I$) and the delayed synaptic variables act on the slow time scale (due to point 3. in Lemma 4). Therefore $(1, 1)$ must be an equilibrium point for (u_A, u_B) in the fast subsystem with no inputs at time $\beta + h$, and must satisfy the condition given in Section 4.3 in the main text : $a - bs_A(\beta - D + h) \geq \theta$ and $a - bs_B(\beta - D + h) \geq \theta$. Taking the limit as $h \to 0$ concludes the first part of the proof.

(⇐) Point 1 of Lemma 4 guarantees both unit being ON at time $t = \beta$. Since $a - bs_B(\beta) \geq \theta$ and $a - bs_A(\beta) \geq \theta$ we have that $(1, 1)$ is a stable fixed point for the fast subsystem 1. Moreover, from point 3 of Lemma 4 $s_A(t - D)$ and $s_B(t - D)$ are monotonically decreasing for $t \in [\beta, t^* + D]$, where $t^* = \min\{t^*_A, t^*_B\}$. Thus, on the fast time scale, $a - bs_B(t - D) \geq \theta$ and $a - bs_A(t - D) \geq \theta$, which implies that $(1, 1)$ is a stable equilibrium for the system in $[\beta, t^* + D]$. Since $TD < D$, $t^* + D > \beta$. Therefore, there $\exists t \in [\beta, t^* + D] \in \mathbb{R} - I$ where both units are ON, ending this part of the proof.

Lastly we prove the remaining claims of the Lemma. We already proved that both units are ON in $[\beta, t^* + D]$ in (⇐) above. To prove the remaining claims we assume $t^* = t^*_A$ (a similar proof holds if $t^* = t^*_B$). At time $t = t^* + D$, $s_A(t - D)$ jumps up to 1. Since $a - bs_A(t - D) = a - b < \theta$ due to condition U_1 in Remark 2.1 in the main text, $(0, 0)$ is the only equilibrium at time t. Therefore the B units instantaneously turns OFF at time t. For Theorem 1 in the main text , also the A unit turns OFF instantantaneously after a small delay $\delta \sim \tau$. Both units are OFF in $[t^* + D + \delta, t_{up}]$. By taking the limit $\tau \to 0$ we thus have that A and B are OFF in $(t^* + D, t_{up}]$ □

1.10 Proof of the remaining claims of Theorem 6

We restate Theorem 6 in the main text for clarity.

Theorem 7 *There is an injective map:*

$$\rho\colon SM \to B(2,4)$$
$$\psi \mapsto V = \left[V_1 \;\middle|\; V_2\right] = \left[\begin{array}{cc|cc} x_A^1 & y_A^1 & x_A^2 & y_A^2 \\ x_B^1 & y_B^1 & x_B^2 & y_B^2 \end{array}\right]$$

Where, for $i=1,2$, V_i are the matrix forms of ψ during the interval I_i defined in identities (9) in the main text , and:

$$s_B^{i\pm} = N^{\pm} y_B^j + M^{\pm}(1-y_B^j) y_B^i, \quad \textit{and} \quad s_A^{i\pm} = N^{\pm} y_A^j + M^{\pm}(1-y_A^j) y_A^i, \quad \forall i,j=1,2, i\neq j \tag{6}$$

In addition,

$$Im(\rho) = \Omega \stackrel{\text{def}}{=} \{V = \left[V_1 \;\middle|\; V_2\right] : V_1 \in Im(\rho^{I_1}), V_2 \in Im(\rho^{I_1}) \textit{ satisfying 1-4 below}\}$$

1 $y_A^1 = y_B^2 = 1 \Rightarrow x_A^1 = x_B^2$ *and* $y_A^2 = y_B^1 = 1 \Rightarrow x_A^2 = x_B^1$
2 $y_B^1 = y_B^2 \Rightarrow x_A^1 \geq x_A^2$ *and* $y_A^1 = y_A^2 \Rightarrow x_B^2 \geq x_B^1$
3 $y_A^2 = 1 \Rightarrow x_B^1 \leq r$ *and* $y_B^1 = 1 \Rightarrow x_A^2 \leq r$*, for any entry* r *in* V
4 $y_A^2 = y_B^2$*,* $y_A^1 = y_B^1 \Rightarrow x_A^1 \geq x_B^1$ *and* $x_B^2 \geq x_A^2$

Proof Here we prove equations 6 and conditions 1-4. The remaining claims of the theorem are proven in the main text. From Theorem 6 in the main text it is clear that the map $\rho = \rho(\psi)$ is well defined and injective. We now prove 6 for $i=2$, $j=1$ and s_B, since all other cases are similar. That is:

$$s_B^{2\pm} = N^{\pm} y_B^1 + M^{\pm}(1-y_B^1) y_B^2$$

Since y_B^1 and y_B^2 are binary, we have three cases to consider:

- Case $y_B^1 = 1$. From Remark 6.2 in the main text , $y_B^1 = 1$ implies the B unit to be ON at time TD. Since ϕ is SHORT the B unit turns OFF at time TD, and due to Theorem 1 in the main text it remains OFF $\forall t \in (TD, TR]$. Thus the delayed synaptic variable $s_B(t-D)$ is equal ~ 1 at time $TD+D$ and decays (slowly) in the interval I_2, evolving according to:

  $$s_B(t-D) = e^{-(t-TD-D)/\tau_i}, \quad \forall t \in I_2$$

  Thus evaluating this function at times $TR \in I_2$ and $TR+TD \in I_2$ yields:

  $$s_B^{2-} = s_B(TR-D) = N^- \quad \text{and} \quad s_B^{2+} = s_B(TR+TD-D) = N^+.$$

- Case $y_B^1 = 0$ and $y_B^2 = 1$. With a proof similar to the case above, the second condition ($y_B^2 = 1$) implies the B unit being ON at time $TR+TD$, and being OFF $\forall t \in (TR+TD, 2TR]$. The first condition ($y_B^1 = 0$) implies B being OFF at time TD, and therefore $\forall t \in [0, TD]$, due to Lemma Lemma 5 in the main text

. Thus, since ψ is $2TR$-periodic, B must be OFF in $[2TR, 2TR{+}TD]$. Moreover, since ϕ is SHORT, B is OFF in $(TD, TR]\cup(TR{+}TD, 2TR] \subset \mathbb{R}{-}I$. In particular, since ψ is $2TR$-periodic, B must be OFF also in $(2TR{+}TD, 3TR] \subset \mathbb{R}{-}I$. Overall we have that B is ON at time $TR{+}TD$ and OFF during $(TR{+}TD, 3TR]$. Thus the delayed synaptic variable $s_B(t{-}D)$ is equal ~ 1 at time $TD{+}D$ and decays (slowly) in the interval $T=(TR{+}TD{+}D, 3TR{+}D]$, evolving according to:

$$s_B(t{-}D) = \; e^{-(t-TR-TD-D)/\tau_i}, \quad \forall t \in T$$

Since $TD{+}D<TR$ and $TD<D$ we have $3TR \in T$ and $3TR{+}TD \in T$. Evaluating $s_B(t{-}D)$ at these times leads to $s_B(3TR{-}D)=M^-$ and $s_B(3TR{+}TD{-}D)=M^+$. Therefore the $2TR$ periodicity of ψ implies:

$$s_B^{2-}=s_B(TR-D)=M^- \quad \text{and} \quad s_B^{2+}=s_B(TR+TD-D)=M^+.$$

- Case $y_B^1=0$ and $y_B^2=0$. These conditions imply B being OFF during both $[0, TD]$ and $[TR, TR{+}TD]$. Moreover it must be OFF also in $[TD, TR] \cup [TR{+}TD, 2TR] \subset \mathbb{R}-I$ since ϕ is SHORT. Overall, the B unit is thus OFF $\forall t \in [0, 3TR]$. This means that the delayed synaptic variables (s_A, s_B) follow the slow subsystem, which have only one possible periodic solution: the fixed point $(0, 0)$. This leads to $s_B=0$.

We now show that the entries of $V = \rho(\psi)$ satisfy conditions 1-4, which proves that $Im(\rho) \subseteq \Omega$. We only prove one of the two statements for points 1,2 and 3. The proof of second statements is analogous. We recall that, given the definition of function f and g given in equation 8 in the main text , the 1st and 3rd columns of V are defined by:

$$x_A^1 = H(c - bs_B^{1-}), \quad x_B^1 = H(d - bs_A^{1-}), \quad x_A^2 = H(d - bs_B^{2-}), \quad x_B^2 = H(c - bs_A^{2-})$$

1 Given the x_A^1 and x_B^2 equations above, we need to prove $s_B^{1-} = s_A^{2-}$. Assuming $y_A^1=y_B^2=1$, from equations 6 we have:

$$s_B^{1-} = N^- y_B^2 + M^-(1 - y_B^2)y_B^1 = N^- = N^- y_A^1 + M^-(1 - y_A^1)y_A^2 = s_A^{2-}$$

2 If $y_B^1=y_B^2$ simple substitutions in 6 lead to $s_B^{1-}=s_B^{2-}$. Since $c\geq d$ we have:

$$x_A^1 = H(c - bs_B^{1-}) = H(c - bs_B^{2-}) \geq H(d - bs_B^{2-}) = x_A^2$$

3 Substituting $y_A^2=1$ in the formula for s_A^{1-} in 6 implies $s_A^{1-}=N^-$ and $s_B^{i-}, s_A^{i-} \leq N^-=s_A^{1-}$, $\forall i=1,2$. The latter inequalities and $c\geq d$ imply

$$x_B^1 \leq x_B^i, \quad x_B^1 \leq x_A^i, \quad \forall i=1,2,$$

since V_1 and V_2 are matrix forms of ψ in I_1 and I_2, respectively, their entries satisfy the first two conditions of equations 12 in the main text , which imply $x_B^i \leq y_B^i$ and $x_A^i \leq y_A^i$, $\forall i = 1, 2$. This proves that $x_B^1 \leq y_B^i$ and $x_B^1 \leq y_A^i$, $\forall i=1,2$, and concludes the proof.

4 If $y_A^2 = y_B^2$ and $y_A^1 = y_B^1$, simple substitutions in 6 lead to $s_B^{1-} = s_A^{1-}$ and $s_B^{2-} = s_A^{2-}$. These equalities, together with $c \geq d$ imply:

$$x_A^1 = H(c{-}bs_B^{1-}) \geq H(d{-}bs_A^{1-}) = x_B^1 \quad \text{and} \quad x_B^2 = H(c{-}bs_A^{2-}) \geq H(d{-}bs_B^{2-}) = x_A^2$$

End □

1.11 Proof of conditions C_9 and C_{10}

The middle row of Table 4 in the main text shows the states' existence conditions in the intervals I_1 and I_2. However, they do not guarantee that units A and B are OFF outside these intervals (ie being SHORT). From Lemma 6 some states need an additional existence condition to guarantee them being SHORT. If during both intervals I_1 and I_2 unit A or B is OFF the first condition of Lemma 6 is not satisfied, thus the state is SHORT with no extra condition. These considerations hold for S, AP and I. If both units are ON in I_1 or I_2 the second condition of Lemma 6 must be false for each tone interval $I = [\alpha, \beta]$ ($I = I_1$ or I_2) in which both units are ON, that is:

$$\min\{a - bs_A(\beta - D), a - bs_B(\beta - D)\} < \theta \tag{7}$$

This condition is expressed differently for each MAIN state in Table 4 in the main text .

- For SB and SD both units turn ON during interval I_1 (I_2 for their conjugate state). Equations 6 lead to $s_A(TD - D) = s_B(TD - D) = M^+$. Thus condition $C_9 < 0$ guarantees that inequalities 7 are satisfied.
- For AS and ASD both units are ON during I_2 (I_1 for their conjugate state). To guarantee condition 7 at time $\beta = TR + TD$ one notices that equations 6 give $s_A(TR + TD - D) = N^+$ and $s_B(TR + TD - D) = M^+$. Thus, condition $C_{10} < 0$ guarantees that condition 7 is satisfied.
- For states ID and IB we notice that condition 7 is symmetrical on both intervals I_1 and I_2. Thus we may restrict the study on interval I_1. Similar to the two previous cases the application of equations 6 gives $s_A(TD - D) = s_B(TD - D) = N^+$. Thus we obtain $C_{10} < 0$.

1.12 Multistability

Theorem 8 (Multistability) *The state I may coexist with SB or SD. Any other pair of 2TR-periodic SHORT MAIN states cannot coexist.*

Proof The inequalities shown in black in Table 1 report all the existence conditions for MAIN SHORT states analyzed in the main manuscript and summarized in Table 4 in the main text . Using the properties $a \geq 0$, $N^+ \geq M^+$ and $c \geq d$ on the quantities $C_i^{\pm}$ defined in Identities 16 in the main text one can easily show that

$$1)\ C_2^- \geq C_8^- \quad 2)\ C_3^+ \geq C_6^+ \quad 3)\ C_3^- \geq C_7^- \quad 4)\ C_3^- \geq C_7^- \quad 5)\ C_5^- \geq C_7^-, \tag{8}$$

which imply the inequalities reported in blue in Table 1.

C	S	SB	SD	AP	AS	ASD	I	ID	IB
1	$C_1 < \theta$						$C_1 \geq \theta$		
2	$C_2^+ < \theta$	$C_2^- \geq \theta$	$C_2^- \geq \theta$	$C_2^+ < \theta$	$C_2^- \geq \theta$	$C_2^- \geq \theta$			
3	$C_3^+ < \theta$	$C_3^+ < \theta$	$C_3^+ < \theta$	$C_3^- \geq \theta$	$C_3^- \geq \theta$	$C_3^- \geq \theta$	$C_3^- < \theta$	$C_3^- \geq \theta$	$C_3^- \geq \theta$
4			$C_4^- \geq \theta$						
5				$C_5^+ < \theta$	$C_5^+ < \theta$	$C_5^+ < \theta$		$C_5^- \geq \theta$	$C_5^- \geq \theta$
6							$C_6^+ < \theta$		
7								$C_7^- < \theta$	$C_7^- \geq \theta$
8		$C_8^- \geq \theta$	$C_8^- < \theta$		$C_8^- \geq \theta$	$C_8^- < \theta$			

Table 1 Existence conditions for MAIN SHORT states (black) and of the conditions derived from 8 (blue).

Inspecting this tables demonstrates that for each pair of MAIN SHORT states (ψ_1, ψ_2) except (I, SB) and (I, SD) there exist at least one index i for which either (a) $C_i^- < \theta$ for ψ_1 (ψ_2) and $C_i^- \geq \theta$ for ψ_1 (ψ_2) or (b) $C_i^+ < \theta$ for ψ_1 (ψ_2) and $C_i^- \geq \theta$ for ψ_1 (ψ_2). Both (a) and (b) lead to conditions that cannot be satisfied simultaneously in the parameter space. This is obvious for case (a). For case (b) this holds because, since $N^- \geq N^+$ and $M^- \geq M^+$, we have $C_i^- \leq C_i^+$, $\forall i = 2, .., 8$. Figure 9C shows the stability regions for states I, SB and S at varying c and DF, demonstrating that bistability between the pairs (I, SB) and (I, SD) can occur (note I and SD have a conjugate, hence we talk of *multistability* for this Theorem). □

1.13 Analysis of $2TR$-periodic SHORT CONNECT states

The analysis for $2TR$-periodic SHORT CONNECT states is similar to that of SHORT MAIN states presented in the main text. The next theorem is similar to Theorem 7 and proves the existence conditions for all possible SHORT CONNECT states. Table 2 shows names (first row) and matrix forms (second row) of all possible $2TR$-periodic SHORT CONNECT states. We omit time histories for these states because they can be visualized from their matrix form (see Remark 6.2 in the main text). For simplicity we can extend the matrix for of MAIN states to CONNECT states, as stated in the next remark.

Remark 1.1 (Matrix form extension for MAIN states) MAIN states can also be equivalently represented using the same 2×3 matrix form W defined for CONNECT states in the previous theorem, by replacing the definition of z_A and z_B with $z_A = H(ay_B + f(\bar{s}_B))$ and $z_B = H(ay_A + g(\bar{s}_A))$. One can check that each existence condition M_{1-6} given in 5 defines one of the following 2×3 matrices:

$$(M_1) \begin{bmatrix} 1 & 1 & 1 \\ 1 & 1 & 1 \end{bmatrix} (M_2) \begin{bmatrix} 1 & 1 & 1 \\ 0 & 1 & 1 \end{bmatrix} (M_3) \begin{bmatrix} 0 & 1 & 1 \\ 1 & 1 & 1 \end{bmatrix} (M_4) \begin{bmatrix} 1 & 1 & 1 \\ 0 & 0 & 0 \end{bmatrix} (M_5) \begin{bmatrix} 0 & 0 & 0 \\ 1 & 1 & 1 \end{bmatrix} (M_6) \begin{bmatrix} 0 & 0 & 0 \\ 0 & 0 & 0 \end{bmatrix}$$

This result guarantees that we can represent all the states in the system using a general 2×3 matrix form (used in Section 7 of the main text).

Theorem 9 *There is an injective map:*

$$\varphi\colon SC \to B(2,6)$$
$$\psi \mapsto W = \begin{bmatrix} W_1 & \big| & W_1 \end{bmatrix} = \left[\begin{array}{ccc|ccc} x_A^1 & y_A^1 & z_A^1 & x_A^2 & y_A^2 & z_A^2 \\ x_B^1 & y_B^1 & z_B^1 & x_B^2 & y_B^2 & z_B^2 \end{array}\right]$$

Where, for $i=1,2$, W_i *is the matrix forms of* ψ *during the interval* I_i *defined in 5, and:*

$$s_B^{i\pm} = N^{\pm} z_B^j + M^{\pm}(1 - z_B^j) z_B^i, \quad \text{and} \quad s_A^{i\pm} = N^{\pm} z_A^j + M^{\pm}(1 - z_A^j) z_A^i, \quad \forall i,j = 1,2, i \neq j \tag{9}$$

In addition, let φ^{I_1} (φ^{I_2}) *be the map defined in Theorem 5 for* ψ *in* I_1 (I_2)*. Then:*

$$Im(\varphi) = \Gamma_{2TR} \stackrel{\text{def}}{=} \{W = \begin{bmatrix} W_1 & \big| & W_2 \end{bmatrix} : W_1 \in Im(\varphi^{I_1}), W_2 \in Im(\varphi^{I_1}) \text{ satisfy conditions 1-11}\}$$

1 *(a)* $z_A^i \geq y_A^i \geq x_A^i$ *and (b)* $z_B^i \geq y_B^i \geq x_B^i$, *for* $i = 1, 2$
2 *(a) If* $x_A^i = x_B^i = 0 \Rightarrow y_A^i = y_B^i = 0$, *for* $i = 1, 2$
3 *(a) If* $z_A^1 = z_B^2 = 1 \Rightarrow x_A^1 = x_B^2$ *and (b) if* $z_A^2 = z_B^2 = 1 \Rightarrow x_A^2 = x_B^1$
4 *(a) If* $z_B^1 = z_B^2 \Rightarrow x_A^1 \geq x_A^2$ *and (b) if* $z_A^1 = z_A^2 \Rightarrow x_B^2 \geq x_A^1$
5 *(a) If* $z_A^2 = 1 \Rightarrow x_B^1 \leq r$ *and (b) if* $z_B^1 = 1 \Rightarrow x_A^2 \leq r$, *for any entry* r *in* V
6 *If* $z_A^2 = z_B^2$ *and* $z_A^1 = z_B^2 \Rightarrow x_A^1 \geq x_B^1$ *and* $x_B^2 \geq x_A^2$
7 *If* $z_A^1 > y_A^1$ *or* $z_A^2 > y_A^2$ *or* $z_B^1 > y_B^1$ *or* $z_B^2 > y_B^2$
8 *(a)* $z_A^1 \neq 0$ *or* $z_A^2 \neq 0$ *and (b)* $z_B^1 \neq 0$ *or* $z_B^2 \neq 0$
9 *(a)* $z_A^1 = z_B^1 = 1, y_A^2 = y_B^2 \Rightarrow z_B^2 \geq z_A^2$ *and (b)* $z_A^2 = z_B^2 = 1, y_A^1 = y_B^1 \Rightarrow z_A^1 \geq z_B^1$
10 $z_A^2 = 1, z_B^1 = 1 \Rightarrow z_B^2 = 1$
11 $z_A^1 = z_B^2, z_B^1 = z_A^2, x_A^1 = x_B^2 \Rightarrow y_A^2 = y_B^1$

Proof By definition, for each state $\psi \in SC$ at least one unit turns ON at some time $t^* \in (0, TD] \cup (TR, TR{+}TD]$. This means that ψ may be MAIN during interval I_1 (I_2) and CONNECT during interval I_2 (I_1), or CONNECT during both intervals I_1 and I_2. In the latter scenario Theorem 5 implies that ψ has a 2 by 3 matrix form W_1 (W_2) defined during interval I_1 (I_2). If ψ is MAIN during I_1 (I_2), Remark 1.1 guarantees that ψ can still be represented during interval I_1 by the same matrix form of CONNECT states given in Theorem 1.7. These considerations guarantee that the transformation given in Theorem 1.7 can be applied to both intervals I_1 and I_2, thus proving that the map φ is well-defined and injective.

We skip the proof of the identities 9, since it is analogous the one given in the proof of identies 6 of Theorem 7. We now prove that each matrix $W \in Im(\varphi)$ satisfies conditions 9. For conditions 1-5 and 8-9 we prove only conditions (a) since the (b) ones are analogous. The proof of the first two conditions follows trivially from the definition of the entries of W. We thus prove the other conditions below

3 $z_A^1 = z_B^2 = 1 \Rightarrow s_A^{2-} = s_B^{1-} = N^- \Rightarrow x_B^1 = x_A^1 = H(c - bN^-)$
4 $z_B^1 = z_B^2 \Rightarrow s_B^{1-} = s_B^{2-} \Rightarrow x_A^1 = H(c - bs_B^{1-}) \geq H(d - bs_B^{2-}) = x_A^2$

5 Since $z_A^1 = 1 \Rightarrow s_A^{1-} = N^-$. Therefore, $x_B^1 = H(d - N^-)$. Any entry r of V is either $H(c - s_A^{i\pm})$, $H(d - s_A^{i\pm})$, $H(c - s_B^{i\pm})$ or $H(d - s_B^{i\pm})$, for some $i = 1, 2$. Since $s_A^{i\pm}, s_B^{i\pm} \leq N^-$ and $d \leq c$, we must have that $r \geq H(d - N^-) = z_A^1$

6 Given $z_A^2 = z_B^2$ and $z_A^1 = z_B^1$ and $c \geq d$ we have that

$$x_A = H(c - bN^- z_B^2 - bM^-(1 - z_B^2) z_B^1) \geq H(d - bN^- z_A^2 - bM^-(1 - z_A^2) z_A^1) = x_B$$

7 By definition, for any CONNECT state s at least one must turn ON within the interval $I = I_1$ or $I = I_2$ (or both). If $I = I_1$, from Theorem 5 we have that the matrix form $W_1 \in \Gamma$. In particular, it must satisfy $y_A^1 < z_A^1$ or $y_B^1 < z_B^1$. Similarly, if $I = I_2$, then $W_2 \in \Gamma$ and $y_A^2 < z_A^2$ or $y_B^2 < z_B^2$

8 By contradiction suppose there exist a CONNECT state s such that $z_A^i = 0$, for $i = 1, 2$. This leads to $s_A^{i\pm} = 0$ and to $x_A^i = y_A^i = 0$ (from 1). Thus, since we hypothesise $c \geq \theta$ then $x_B^2 = H(c) = 1$, which guarantees $z_B^2 = y_B^2 = 1$ (again from 1). Since $z_A = 0$ we also have that $x_A^1 = 0$. This leads to $x_B^2 = y_B^2 = z_B^2 = H(d)$. This leads to the matrix form

$$\left[\begin{array}{ccc|ccc} 0 & 0 & 0 & 0 & 0 & 0 \\ H(d) & H(d) & H(d) & 1 & 1 & 1 \end{array}\right]$$

Since $y_A^i = z_A^i$ or $y_B^i = z_B^i$ for $i = 1, 2$, we have $W_1 \notin Im(\varphi^{I_1})$ and $W_2 \notin Im(\varphi^{I_2})$, which is absurd.

9 Given $z_A^1 = z_B^1 = 1$ we have $s_A^{1+} = s_A^{1+} = N^+$. Since $y_A^2 = y_B^2$ and $d \leq c$ we have that $z_B^2 = H(a y_A^2 - bN^+ + c) \geq H(a y_B^2 - bN^+ + d) = z_A^2$

10 If $y_B^2 = 1$ from (1) we have $z_B^2 = 1$, which proves the claim. Thus we can assume that $y_B^2 = 0$. Condition $z_B^1 = 1$ implies $s_B^{2+} = N^+$. This identity and $y_B^2 = 0$ implies that $z_A^2 = H(d - bN^+)$. Thus from the hypothesis $z_A^2 = 1$ we have $d - bN^+ \geq \theta$. Moreover, since $d \leq c$, $a y_B^2 \geq 0$, and $s_A^{2+} \leq N^+$ we must have $z_B^2 = H(a y_B^2 + c - b s_A^{2+}) \geq H(d - bN^+) = 1$

11 Given $z_A^1 = z_B^2, z_B^1 = z_A^2, x_A^1 = x_B^2$ it obviously follows that

$$y_A^2 = H(a x_B^2 - bN^- z_B^1 - bM^-(1 - z_B^1) z_B^2 + d) = H(a x_A^1 - bN^- z_A^2 - bM^-(1 - z_A^2) z_A^1 + d) = y_B^1$$

Next, we algorithmically find all matrices in Γ_{2TR}. We proceed by generating all 2 by 6 binary matrices matrices $W = \left[W_1 \,\middle|\, W_2\right]$ with entries satysfying conditions 1-11. In total, we find that $|\Gamma_{2TR}| = 15$, thus implying $|Im(\varphi)| \leq 15$.

Due to the model's symmetry, for any matrix $W = \varphi(\psi)$ of an asymmetrical state ψ there exist a matrix $W' \in \Gamma_{2TR}$ image of the state ψ' conjugate to ψ, and this matrix is defined by swapping the first row of W_1 with the second row of W_2 and the second row of W_1 with the first row of W_2. Notably, both ψ and ψ', and thus also W and W', exist under the same parameter conditions. The top rows of Table 4 in the main text shows all matrices $V \in \Omega$ that are an image of either of a symmetrical state or one of two conjugate states and their corresponding names (1st row).

The analysis of existence conditions for SHORT CONNECT states is slightly more involved than the one done in Theorem 7 for SHORT MAIN states. The reason is that for the well-definedness conditions for the entries of each SHORT MAIN state's

ZcS^*	$ZcAP$	$ZcAS^*$	ZcI	$ScAS^*$	$SDcAS^*$	$ScSD^*$	$APcAS^*$	$APcI$
001000<br>001000	001000<br>000001	001001<br>000001	001001<br>001001	001111<br>000001	001111<br>000011	111000<br>001000	111001<br>000111	111001<br>001111
$C_4^- < \theta$<br>$C_4^+ \geq \theta$<br>$C_2^+ \geq \theta$	$C_2^+ < \theta$<br>$C_3^- < \theta$<br>$C_3^+ \geq \theta$	see 10	$C_3^- < \theta$<br>$C_3^+ \geq \theta$<br>$C_5^+ \geq \theta$	$C_3^+ \geq \theta$<br>$C_5^+ < \theta$<br>$C_8^- \geq \theta$<br>$C_6^- < \theta$	$C_3^- < \theta$<br>$C_3^+ \geq \theta$<br>$C_5^+ < \theta$<br>$C_8^- \geq \theta$<br>$C_6^- \geq \theta$	$C_4^- \geq \theta$<br>$C_2^- < \theta$<br>$C_2^+ \geq \theta$<br>$C_3^+ < \theta$	$C_3^- \geq \theta$<br>$C_5^+ < \theta$<br>$C_2^- < \theta$<br>$C_2^+ \geq \theta$	$C_3^- \geq \theta$<br>$C_5^- < \theta$<br>$C_5^+ \geq \theta$
$C_9 < \theta$	–	$C_{10} < \theta$	$C_{10} < \theta$	$C_{10} < \theta$	$C_{10} < \theta$	$C_9 < \theta$	$C_{10} < \theta$	$C_{10} < \theta$

Table 2 Matrix form of $2TR$-periodic SHORT CONNECT states (* asymmetrical states). These states' dynamics is connecting branches of pairs of MAIN states or the inactive state (Z). For example ZcS connects the inactive state with one of the segregated states S, SB or SD. The names chosen for CONNECT states (first row) contains the names of the two MAIN states separated by the letter c.

matrix form are necessary and sufficient for determining the dynamics of each state in I_1 and I_2. In the case of CONNECT states, this property is not valid. Therefore, we analyse each of the remaining 15 matrices given in Table 2 separately using conditions $C_{1\text{–}5}$ and $M_{1\text{–}6}$. Similar to the proof of formulas 6 of Theorem 7, one may show that that variables $s_A(t-D)$ and $s_B(t-D)$ of any SHORT CONNECT states are monotonically decreasing and depend on functions

$$N(t) = e^{(-TR\text{–}D\text{–}t)/\tau_i} \quad \text{and} \quad M(t) = e^{(-2TR\text{–}D\text{–}t)/\tau_i}.$$

More precisely, these variables satisfy the following $\forall t \in I_1 \cup I_2$:

$$s_B(t\text{–}D) = N(t)z_B^j + M(t)(1-z_B^j)z_B^i, \text{ and } s_A(t\text{–}D) = N(t)z_A^j + M(t)(1-z_A^j)z_A^i, \ \forall i,j = 1,2, i \neq j.$$

Obviously, this is an extension of the proof of 9, since these quantities can be obtained by evaluating the equations above at time $t = 0, TD, TR$ and $TR + TD$. Using these identities we now prove that the existence conditions for each state shown in the third row of Table 2.

1 **ZcS** - This state is CONNECT during interval I_1 (satisfying condition C_5) and MAIN during interval I_2 (satisfying condition M_6). Since $z_A^1 = z_B^1 = 1$ and $z_A^2 = z_B^2 = 0$ we have

$$s_A(t\text{–}D) = s_B(t\text{–}D) = M(t), \ \forall t \in I_1 \quad \text{and} \quad s_A(t\text{–}D) = s_B(t\text{–}D) = N(t), \forall t \in I_2.$$

In particular, evaluating these equations at time $t = 0, TD, TR$ and $TR + TD$ we obtain

$$s_A^{1\pm} = s_B^{1\pm} = M^{\pm} \quad \text{and} \quad s_A^{2\pm} = s_B^{2\pm} = N^{\pm}.$$

Condition C_5 on the interval I_1 requires that A(B) turns ON at the (unique) times $t^*(s^*)$ in $(0, TD]$. It must be that $t^* \leq s^*$. Indeed, on the contrary suppose that B turns ON at time $s^* < t^*$. Thus we must have $d\text{–}bs_A(s^*\text{–}D) = \theta$ (i.e. point $(0,1)$ is an equilibrium for the fast subsystem at time s^*) and $c\text{–}bs_B(s^*\text{–}D) < \theta$ (i.e. point $(1,0)$ is not an equilibrium for the fast subsystem at time s^*). This is absurd because $c \geq d$ and $s_A(s^* - D) = s_B(s^* - D) = M(s^*)$. Thus necessary and sufficient existence conditions for ZcS are given by conditions C_5 under the case $t^* \leq s^*$, which are

$$C_4^- = c - bM^- < \theta, \quad C_4^+ = c - bM^+ \geq \theta \quad \text{and} \quad C_2^+ = a - bM^+ d \geq \theta.$$

Lastly we need to ensure that ZcS satisfies condition M_6 on the interval I_1. More precisely, these conditions are $c-bN^+<\theta$ and $d-bN^+<\theta$. We notice that, since $N^+ \geq M^-$, both of these conditions automatically hold due to $C_4^- = c-bM^- < \theta$.

2 **ZcAP** - This state is CONNECT for both intervals I_1 (satisfying condition C_4) and I_2 (satisfying condition C_3). Since $z_A^1 = z_B^2 = 1$ and $z_A^2 = z_B^1 = 0$ we have $s_B^{1\pm} = s_A^{2\pm} = N^\pm$ and $s_A^{1+} = s_B^{2+} = M^+$. Thus from the conditions given in C_3 we have that

$$C_3^- = c-bN^- < \theta, \quad C_3^+ = c-bN^+ \geq \theta, \quad C_2^+ = a-bM^+ + d < \theta.$$

3 **ZcI** - This state is CONNECT for both intervals I_1 (satisfying condition C_5) and I_2 (satisfying condition C_5). Conditions $z_A^1 = z_B^1 = z_A^2 = z_B^2 = 1$ lead to

$$s_A(t-D) = s_B(t-D) = N(t) \quad \forall t \in I_1 \cup I_2.$$

In particular, evaluating these equations at time $t=0, TD, TR$ and $TR+TD$ we obtain $s_A^{1\pm} = s_B^{1\pm} = s_A^{2\pm} = s_B^{2\pm} = N^\pm$. Since the synaptic variables evolve equally on both intervals and due to the model's symmetry it must be that A and B turn ON at the same time t^* during intervals I_1 and I_2 respectively, and B and A turn ON at the same time s^* during intervals I_1 and I_2 respectively (applying condition C_5 on both intervals). Similar considerations made for the case ZcS lead to $t^* \leq s^*$. Thus the existence conditions for ZcAP are given by conditions C_5 under the case $t^* \leq s^*$, and they are

$$C_3^- = c-bN^- < \theta, \quad C_3^+ = c-bN^+ \geq \theta, \quad C_5^+ = a-bN^+ + d \geq \theta.$$

4 **ScAS** - This state is CONNECT for both intervals I_1 (satisfying condition C_4) and I_2 (satisfying condition C_1). Since $z_A^1 = z_A^2 = z_B^2 = 1$ and $z_B^1 = 0$ we have $s_A^{1\pm} = s_A^{2\pm} = s_B^{1\pm} = N^\pm$ and $s_B^{2\pm} = M^\pm$. Condition C_4 on interval I_1 leads to (1) $c-bN^- < \theta$, (2) $c-bN^+ \geq \theta$ and (3) $a-bN^+ + d < \theta$. Condition C_1 on interval I_2 lead to (4) $d-bM^- \geq \theta$, (5) $a-bN^- + c < \theta$ and (6) $a-bN^+ + c \geq \theta$. Conditions (1) and (6) can be discarded because they derive respectively from conditions (5) and (2) (using the properties $N^- \geq N^+$ and $a \geq 0$). Thus, the remaining conditions are

$$C_3^+ = c-bN^+ \geq \theta, \quad C_5^+ = a-bN^+ + d < \theta, \quad C_8^- = d-bM^- \geq \theta \quad \text{and} \quad C_6^- = a-bN^- + c < \theta.$$

5 **SDcAS** - This state is CONNECT for interval I_1 (satisfying condition C_4) and MAIN for interval I_2 (satisfying condition M_2). Like in the case of ScAS, since $z_A^1 = z_A^2 = z_B^2 = 1$ and $z_B^1 = 0$ we have $s_A^{1\pm} = s_A^{2\pm} = s_B^{1\pm} = N^\pm$ and $s_B^{2\pm} = M^\pm$. Condition C_4 on the interval I_1 implies conditions (1-3) in ScAS. Condition M_2 on interval I_2 implies (4) $d-bM^- \geq \theta$, (5) $c-bN^- < \theta$ and (6) $a-bN^- + c \geq \theta$. Obviously, condition (1) can be discarded because it is the same as (5), and the remaining conditions thus are

$$C_3^- = c-bN^- < \theta, C_3^+ = c-bN^+ \geq \theta, C_5^+ = a-bN^+ + d < \theta, C_8^- = d-bM^- \geq \theta, C_6^- = a-bN^- + c \geq \theta.$$

6 **ScSD** - This state is CONNECT for interval I_1 (satisfying condition C_1) and MAIN for interval I_2 (satisfying condition M_6). As in case ZcS we have $s_A^{1\pm} = s_B^{1\pm} = M^{\pm}$ and $s_A^{2\pm} = s_B^{2\pm} = N^{\pm}$. Condition C_1 on interval I_1 leads to $c - bM^- \geq \theta$, $a - bM^- + d < \theta$ and $a - bM^+ + d \geq \theta$. Condition M_6 on interval I_2 implies (1) $d - bN^+ < \theta$ and (2) $c - bN^+ < \theta$. Obviously, since $d \leq c$, (2) implies (1), and thus (1) can be discarded. The remaing conditions are

$$C_4^- = c - bM^- \geq \theta, \quad C_2^- = a - bM^- + d < \theta, \quad C_2^+ = a - bM^+ + d \geq \theta, \quad C_3^+ = c - bN^+ < \theta.$$

7 **APcAS** - This state is CONNECT for interval I_1 (satisfying condition M_5) and MAIN for interval I_2 (satisfying condition C_2). Similarly to the case ScAS we have that $s_A^{1\pm} = s_A^{2\pm} = s_B^{1\pm} = N^{\pm}$ and $s_B^{2\pm} = M^{\pm}$. Condition M_5 on interval I_1 leads to $c - bN^- \geq \theta$ and $a - bN^+ + d < \theta$. Condition C_2 on interval I_2 leads to $c - bN^- \geq \theta$ (again), $a - bM^- + d < \theta$ and $a - bM^+ + d \geq \theta$. In summary these conditions are

$$C_3^- = c - bN^- \geq \theta, \quad C_5^+ = a - bN^+ + d < \theta, \quad C_2^- = a - bM^- + d < \theta, \quad C_2^+ = a - bM^+ + d \geq \theta.$$

8 **APcINT** - This state is CONNECT for both intervals I_1 and I_2, satisfying condition C_1 and C_2 respectively. As for ZcI, conditions $z_A^1 = z_B^1 = z_A^2 = z_B^2 = 1$ lead to

$$s_A(t - D) = s_B(t - D) = N(t) \quad \forall t \in I_1 \cup I_2.$$

Thus we obtain $s_A^{1\pm} = s_B^{1\pm} = s_A^{2\pm} = s_B^{2\pm} = N^{\pm}$. Moreover, since the synaptic variables evolve equally on both intervals and due to the model's symmetry it must be that A and B turn ON at the same time t^* during intervals I_1 and I_2 respectively (applying conditions C_{1-2} on I_{1-2}). Moreover conditions C_1 and C_2 are equal and lead to $C_3^- = c - bN^- \geq \theta$, $C_5^- = a - bN^- + d < \theta$ and $C_5^+ = a - bN^+ + d \geq \theta$.

9 **ZcAS** - Showing the existence conditions for this state is the most involved case. This state is CONNECT for both intervals I_1 (satisfying condition C_4) and I_2 (satisfying condition C_5). Since $z_A^1 = z_A^2 = z_B^2 = 1$ and $z_B^1 = 0$ we have

$$s_A(t - D) = N(t) \quad \text{and} \quad s_B(t - D) = M(t), \forall t \in I_2.$$

In particular, evaluating these equations at time $t = 0, TD, TR$ and $TR + TD$ we obtain $s_A^{1\pm} = s_A^{2\pm} = s_B^{1\pm} = N^{\pm}$ and $s_B^{2\pm} = M^{\pm}$. For condition C_5 on I_2 we have that B and A turn ON at times t^* and s^* in $(TR, TR + TD]$, respectively. We have two cases to consider:

- Case $t^* < s^*$. From the evolution of the synaptic variables and since they are monotonically decaying we may express existence conditions as follows:
  (P1) $\exists t^* \in (TR, TR + TD] : c - bN(t^*) = \theta \Leftrightarrow C_3^- = c - bN^- < \theta$ and $C_3^+ = c - bN^+ \geq \theta$.
  (P2) $\forall s \in (0, t^*) : d - bM(s) < \theta \Leftrightarrow d - bM(t^*) < \theta$
  (P3) $\exists s^* \in (t^*, TR + TD] : a - bM(s^*) + d \geq \theta \Leftrightarrow C_2^+ = a - bM^+ + d \geq \theta$

Where (P1) guarantees that B turns ON at t^*, (P2) that A is OFF $\forall s \le t^*, s \in I_2$ and (P3) that A turns ON at time s^*. Thus (P2) guarantees $s^* > t^*$. From (P1) we have that

$$t^* = N^{-1}((c-\theta)/b) = \tau_i \log((c-\theta)/b) + (TR-D)$$

By substituting this identity in (P2) and we obtain that $d - bM(t^*) < \theta$ if and only if $K = c-(d-\theta)e^{TR/\tau_i} > \theta$. Lastly we need to guarantee conditions C_4 on I_1. Two conditions are $C_3^- = c - bN^- < \theta$ and $C_3^+ = c - bN^+ \ge \theta$, which are equivalent to case (P1). The second condition is that $C_5^+ = a - bN^+ + d < \theta$.

- Case $t^* \ge s^*$. Similar to the previous case we can formulate the following conditions:
  (Q1) $\exists s^* \in (TR, TR+TD] : d - bM(s^*) = \theta \Leftrightarrow C_8^- = d - bM^- < \theta$ and $C_8^+ = d - bM^+ \ge \theta$
  (Q2) $\exists t^* \in (s^* - TR, TD] : c - bN(t^*) = \theta \Leftrightarrow c - bN(s^*) < \theta$ and $C_3^+ = c - bN^+ \ge \theta$
  Where (Q1) guarantees that A turns ON at $s^* \in (TR, TR+TD]$ and (Q2) that it turns ON at time t^*, wher $t^* - TR \ge s^*$ (ie one of conditions C_4 on I_1). From (Q1) we have that

$$s^* = N^{-1}((d-\theta)/b) = \tau_i \log((d-\theta)/b) + (2TR - D),$$

  Thus the first condition in (Q2) is equivalent to $K \le \theta$. Condition $C_3^+ \ge \theta$ and $a \ge 0$ imply $a + c - bN^+ \ge \theta$, thus completing conditions C_2 on I_2. Analogously to the previous case, the last condition to be ensures is $C_5^+ = a - bN^+ + d < \theta$.

Thus, in summary, the conditions for both cases are:

$$\begin{cases} C_3^- < \theta, C_3^+ \ge \theta, C_2^+ \ge \theta, C_5^+ < \theta, & \text{if } K > \theta \\ C_8^- < \theta, C_8^+ \ge \theta, C_3^+ \ge \theta, C_5^+ < \theta, & \text{if } K \le \theta. \end{cases} \tag{10}$$

This completes the proof of the existence conditions for ZcAS.
Notably, we numerically simulated each state that correspong to a matrix in Γ_{2TR}, thus proving that this its conditions can be satisfied in a non-empty region of parameters. This proves that $Im(\rho) = \Gamma_{2TR}$. □

1.14 Analysis of $2TR$-periodic MAIN LONG states

In this section we analyze the existence conditions for $2TR$-periodic LONG MAIN states. To do so we use a similar analysis to the one described in the section 7.1 of the main text. The first step is to extend the matrix form definition to LONG states. Due to Lemma 6, LONG states can exist only if there exist one active tone interval $R = I_1$ or $R = I_2$ for which two conditions are satisfied. Let us name $R = [\alpha, \beta]$. The conditions are:

1. Both units must be ON at time β
2. $a - bs_A(\beta - D) \ge \theta$ and $a - bs_B(\beta - D) \ge \theta$

We can then extend the definition of the matrix form of MAIN LONG states by including a last column in the matrix form of SHORT MAIN states. More precisely, the matrix form for a state $\psi \in LM$ is the 2×6 binary matrix V defined as

$$V = \left[V_1 \;\middle|\; \vec{w}^1 \;\middle\|\; V_2 \;\middle|\; \vec{w}^2 \right] = \left[\begin{array}{cc|c||cc|c} x_A^1 & y_A^1 & w^1 & x_A^2 & y_A^2 & w^2 \\ x_B^1 & y_B^1 & w^1 & x_B^2 & y_B^2 & w^2 \end{array} \right]$$

Where V_1 and V_2 are the same matrix forms defined for MAIN SHORT states, respectively, with entries defined in identities (9) in the main text . Entries of the binary vectors $\vec{w}^1$ and $\vec{w}^2$ guarantee that condition 2. is met for LONG states and they are defined by

$$w^1 = H(ay_A^1 - bs_A^{1+})H(ay_B^1 - bs_B^{1+}) \quad \text{and} \quad w^2 = H(ay_A^2 - bs_A^{2+})H(ay_B^2 - bs_B^{2+}). \tag{11}$$

We remind the reader that $s_A^{1+} = s_A(TD–D)$, $s_B^{1+} = s_B(TD–D)$, $s_A^{2+} = s_A(TR+TD–D)$ and $s_B^{2+} = s_B(TR+TD-D)$. These quantities appear also in the definition of the V_1 and V_2 entries. In the case of LONG MAIN states they depend on both $N^\pm$ and $M^\pm$ defined in equations 14 in the main text and on the following quantities:

$$N_L^- = e^{-(TR-2D)/\tau_i},\; N_L^+ = e^{-(TR+TD-2D)/\tau_i},\; M_L^- = e^{-(2TR-2D)/\tau_i},\; M_L^+ = e^{-(2TR+TD-2D)/\tau_i}. \tag{12}$$

We note that $N_L^+ \geq N^+$, $N_L^- \geq N^-$, $M_L^+ \geq M^+$ and $M_L^- \geq M^-$. Using a similar analysis carried to prove equations 6 in Theorem 7 one can easily show that:

$$\begin{aligned} s_B^{i\pm} &= w^j N_L^\pm + (1-w^j)y_B^j N^\pm + (1-w^j)(1-y_B^j)w_B^i M_L^\pm + (1-w^j)(1-y_B^j)(1-w^i)y_B^i M^\pm \\ s_A^{i\pm} &= w^j N_L^\pm + (1-w^j)y_A^j N^\pm + (1-w^j)(1-y_A^j)w^i M_L^\pm + (1-w^j)(1-y_A^j)(1-w^i)y_A^i M^\pm \end{aligned} \tag{13}$$

To analyse LONG MAIN states $\psi \in LM$ we may restrict to the case where the interval R for which properties (1-2) given above are satisfy is $R = I_1$ (the case $R = I_2$ will be analysed using symmetry principles). Properties (1-2) may then be rewritten as (a) both units are ON at time $\beta = TD$, and (b) $a - bs_A^{1+} \geq \theta$ and $a - bs_B^{1+} \geq \theta$. From (a) we have that $(1,1)$ is an equilibrium for the fast subsystem at time TD, which implies that V_1 satisfies one of M_{1-3} during the interval I_1. From (b) we obtain $w^1 = 1$. Before we consider separately each of cases M_{1-3}, we note that the entries of the matrix form of any MAIN LONG state ψ satisfy the properties stated in the next theorem.

Theorem 10 *The matrix form V of any LONG MAIN state $\psi \in LM$ satisfies:*

1 $x_A^2 \leq x_B^2$
2 *If* $w^2 = 1 \Rightarrow x_A^2 = x_B^1, x_B^2 = x_A^1, y_A^2 = y_B^1$ *and* $y_B^2 = y_A^1$
3 $x_A^2 \leq x_B^1$ *and* $x_B^2 \leq x_A^1$
4 *If* $w^2 = y_A^2 = y_B^2 = 0 \Rightarrow x_A^1 \geq x_B^1$
5 $x_A^2 \leq y_A^2$ *and* $x_B^2 \leq y_B^2$

6 $x_A^2 = x_B^2 = 0 \Rightarrow y_A^2 = y_B^2 = 0$

Proof Since $w^1 = 1$, from the identities 13 we have $s_B^{2-} = s_A^{2-} = N_L^-$, which leads to $x_A^2 = H(d\text{–}bN_L^-)$ and $x_B^2 = H(c\text{–}bN_L^-)$. Since $d \le c$, we have (1). Similarly, if $w^2 = 1$, we have $s_B^{1\pm} = s_A^{1\pm} = N_L^\pm$. This implies $x_A^2 = H(d\text{–}bN_L^-) = x_B^1$ and $x_B^2 = H(c\text{–}bN_L^-) = x_A^1$. Analogously, one can easily show that $y_A^2 = y_B^1$ and $y_B^2 = y_A^1$ using the definition of these entries given in identities (9) in the main text . Since $w^1 = 1$ we have that $s_A^{2-} = s_B^{2-} = N_L^- \ge s_A^{1-}$, which proves (3). Under the hypothesis of (4) we have that $s_B^{1\pm} = s_A^{1\pm} = M_L^\pm$. This and $c \ge d$ implies $x_A^1 = H(c - bM_L^-) \ge H(d - bM_L^-) = x_B^1$, proving (4). Since ψ is MAIN, conditions (5-6) derive from Theorem 6 in the main text . □

The previous theorem allow us to restrict the number of possible LONG MAIN states. Indeed the possible matrix forms for states satisfying one of condition M_{1-3} on the interval I_1 and satisfying conditions (1-7) are only the ones shown in the top rows of Table 3. These can be divided into:

- The first 5 matrices in Table 3 correspond to the states satisfying M_1 in I_1
- The last 4 matrices in Table 3 correspond to the states satisfying M_2 in I_1
- ψ cannot satisfy M_3 in I_1 since conditions (1-7) lead to no possible matrix forms

Symmetry arguments lead to the obvious symmetrical conjugates for these states, and they complete the case where both units are ON at time $\beta = TR + TD$, and $a - bs_A^{2+} \ge \theta$ and $a - bs_B^{2+} \ge \theta$.

IL_1	IL_2^*	$ASDL_1^*$	ASL^*	SL^*	IDL_1	IDL_2^*	$ASDL_2^*$	SDL^*
111111<br>111111	111110<br>111110	111010<br>111110	111000<br>111110	111000<br>111000	111011<br>011111	111010<br>011110	111000<br>011110	111000<br>011000
$D_7^- \ge \theta$	$D_7^- \ge \theta$	$D_7^- < \theta$<br>$D_5^- \ge \theta$<br>$D_3^- \ge \theta$	$D_3^- \ge 0$<br>$D_5^+ < \theta$<br>$D_8^- \ge \theta$	$D_3^+ < \theta$<br>$D_8^- \ge \theta$	$D_3^- \ge \theta$<br>$D_7^- < \theta$<br>$D_5^- \ge \theta$	$D_3^- \ge \theta$<br>$C_7^- < \theta$<br>$D_5^- \ge \theta$	$D_3^- \ge \theta$<br>$D_5^- \ge \theta$<br>$D_8^- < \theta$<br>$D_2^- \ge \theta$	$D_4^- \ge \theta$<br>$D_8^- < \theta$<br>$D_2^- \ge \theta$<br>$D_3^+ < \theta$
$D_{10} \ge \theta$	$D_{10} < \theta$<br>$C_{10} \ge \theta$	$D_{10} < \theta$<br>$C_{10} \ge \theta$	$C_{10} \ge \theta$	$D_9 \ge \theta$	$D_{10} \ge \theta$	$D_{10} < \theta$<br>$C_{10} \ge \theta$	$C_{10} \ge \theta$	$D_9 \ge \theta$

Table 3 Matrix form and existence conditions of $2TR$-periodic LONG MAIN states (asymmetrical states in *).

Next we prove the conditions for the MAIN LONG states shown in the middle row of Table 3 using equations 13. For simplicity we write the following conditions using the analogous version of Identities 16 in the main text in the case of LONG states.

$$\begin{gathered} D_2^\pm = a - bM_L^\pm + d, \quad D_3^\pm = c - bN_L^\pm, \quad D_4^\pm = c - bM_L^\pm, \quad D_5^\pm = a - bN_L^\pm + d, \\ D_6^\pm = a - bN_L^\pm + c, \quad D_7^\pm = d - bN_L^\pm, \quad D_8^\pm = d - bM_L^\pm, \quad D_9 = a - bM_L^+ \quad D_{10} = a - bN_L^+ \end{gathered} \tag{14}$$

Next, we prove the existence conditions for each state separately.

- IL_1 - This state satisfies conditions M_1 during both intervals I_1 and I_2. Due to the symmetry of the matrix form conditions M_1 are equal to conditions

M_2. Since $w^2=1$ we have that $s_A^{1\pm}=s_B^{1\pm}=N_L^{\pm}$. From this, conditions M_1 on interval I_1 are $c-bN_L^-\geq\theta$ and $D_7^-=d-bN_L^-\geq\theta$. Since $c\geq d$, the condition $D_7^-\geq\theta$ is sufficient to imply $c-bN_L^-\geq\theta$. Since $y_A^1=y_B^1=1$, the identity $w^1=H(a-bN_L^+)=1$ implies $D_{10}\geq\theta$.

- IL_2 - Analogously to the previous case, this state satisfies conditions M_1 during both intervals I_1 and I_2. Since $w^1=1$, $w^2=0$ and $y_A^2=y_B^2=1$ we have $s_A^{1\pm}=s_B^{1\pm}=N^{\pm}$ and $s_A^{2\pm}=s_B^{2\pm}=N_L^{\pm}$. Since $c\geq d$ and $N_L^-\geq N^-$, conditions M_1 during both intervals I_1 and I_2 are simplified to obtain $D_7^-=d-bN_L^-\geq\theta$. In addition, $w^1=1$ and $w^2=0$ are equvalent to $D_{10}<\theta$ and $C_{10}\geq\theta$.
- $ASDL_1$ - We notice that the same arguments used for IL_2 lead to $D_{10}<\theta$ and $C_{10}\geq\theta$, and to $s_A^{1\pm}=s_B^{1\pm}=N^{\pm}$ and $s_A^{2\pm}=s_B^{2\pm}=N_L^{\pm}$. This state satisfies conditions M_1 during interval I_1 and M_3 during interval I_2. The first set of conditions (M_1) lead to $C_7^-=d-bN^-\geq\theta$ (which implies also the second condition in M_1, ie $c-bN^+\geq\theta$). The second set of conditions (M_3) lead to $D_7^-=d-N_L^-<\theta$, $D_5^-=a+d-N_L^-\geq\theta$ and $D_3^-=c-N_L^-\geq\theta$.
- ASL - This state satisfies conditions M_1 during interval I_1 and M_5 during interval I_2. Since $w^1=1$ we have that $s_A^{2\pm}=s_B^{2\pm}=N_L^{\pm}$. Since $w^2=1$, $y_A^2=0$ and $y_B^2=1$ we have that $s_A^{1\pm}=N^{\pm}$ and $s_B^{1+}=M_L^+$. Conditions leading to M_5 during interval I_2 are $D_3^-=c-bN_L^-\geq 0$ and $D_5^+=a-bN_L^++d<\theta$. Conditions leading to M_1 during I_1 are $c-bN^-\geq\theta$, which is implied by $D_3^-\geq\theta$ (due to $N_L^-\geq N^-$) and $D_8^-=d-M_L^-\geq\theta$. Finally, as in case IL_1, $w^1=1$ implies $D_{10}=a-bN_L^-\geq\theta$ and $a-bM_L^-\geq\theta$. Since $N_L^-\geq M_L^-$ this second condition derives from $D_{10}\geq\theta$ and it can therefore be excluded. Moreover we note that, since $y_A^2=y_A^2=0$, we must have $w^2=0$. Thus no other conditions are required.
- SL - This state satisfies conditions M_1 during interval I_1 and M_6 during interval I_2. Given that $w^1=1$ we have $s_A^{2+}=s_B^{2+}=N_L^+$. Condition M_6 requires $D_3^+=c-bN_L^+<\theta$ (since it implies $d-bN_L^+<\theta$). Since $w^2=0$ and $y_A^2=y_B^2=0$ we have that $s_A^{1-}=s_B^{1-}=M_L^-$. Condition M_1 requires $D_8^-=d–bM_L^+\geq\theta$ (since it implies $c–bM_L^+\geq\theta$). Condition $D_9=d–bM_L^-\geq\theta$ guarantees that $w^1=1$. We note that, since $y_A^2=y_A^2=0$, we must have $w^2=0$ with no extra conditions.
- IDL_1 - This state satisfies conditions M_2 during the interval I_1 and conditions M_3 during the interval I_2. Since this state is symmetrical M_2 and M_3 are give equal conditions. Analogously to the case IL_1 we have that $s_A^{1-}=s_B^{1-}=N_L^-$. Thus conditions for M_2 are $D_3^-=c-bN_L^-\geq\theta$, $D_7^-=d-bN_L^-<\theta$ and $D_5^-=a-bN_L^-+d\geq\theta$. Condition $w^1=1$ leads to $D_{10}=a-bN_L^+\geq\theta$.
- IDL_2 - Analogously to case IL_2 we obtain $s_A^{1\pm}=s_B^{1\pm}=N^{\pm}$ and $s_A^{2\pm}=s_B^{2\pm}=N_L^{\pm}$. This state ($IDL_2$) satisfies conditions M_2 on interval I_1 and M_3 on interval I_2. This leads to $D_3^-=c-bN_L^-\geq\theta$ (which implies $c-bN^-\geq\theta$), $C_7^-=d–bN^-<\theta$ (which implies $d–bN_L^-<\theta$) and $D_5^-=a+d–bN_L^-\geq\theta$ (which implies $d-bN^-<\theta$, hence $y_B^2=1$). Similar arguments to the ones shown in case IL_2 lead to $D_{10}<\theta$ and $C_{10}\geq\theta$
- $ASDL_2$ - As in case ASL we have that $s_A^{2\pm}=s_B^{2\pm}=N_L^{\pm}$, $s_A^{1-}=N^-$ and $s_B^{1-}=M_L^-$. This state satisfies conditions M_2 on interval I_1 and M_5 on interval I_2. For the same arguments as case IDL_2 we must have $D_3^-=c-bN_L^-\geq\theta$. Completing the conditions on I_1 requires $D_8^-=d-bM_L^+<\theta$ and $D_2^-=a-bM_L^-+d\geq\theta$. Completing the conditions on I_2 requires $D_5^-=a+d-bM_L^-\geq\theta$. As in case ASL we also require $D_{10}\geq\theta$.

- SDL - Analogously to case SL we have $s_A^{2+} = s_B^{2+} = N_L^+$, $s_A^{1-} = s_B^{1-} = M_L^-$ and $D_9 \geq \theta$. This state satisfies conditions M_2 during interval I_1 and M_6 during interval I_2. As shown in SL, conditions M_6 on interval I_2 implies $D_3^+ < \theta$. Instead, conditions M_3 on interval I_1 are $D_4^- = c - bM_L^- \geq \theta$, $D_8^- = d - bM_L^- < \theta$ and $D_2^- = a - bM_L^- + d \geq \theta$.

This conlcudes the proof of the existence conditions for all the LONG MAIN states shown in Table 3.

1.15 Analysis of $2TR$-periodic $LM|SC$, $LC|SC$, $LC|LC$ and $LC|SM$ states

As shown in the Section 7 of the main text , $2TR$-periodic states can be SHORT MAIN (SM), SHORT CONNECT (SC), LONG MAIN (LM) or LONG CONNECT (LC) during each interval I_1 and I_2. We define $X|Y$ the set of states satisfying condition X during I_1 and Y during I_2, where $X, Y \in \{SM, SC, LM, LC\}$. In Section 7 of the main text we have the existence conditions of all possible states in some of these sets. More precisely:

- The analysis of $SM|SM$ is summarised in Table 4 in the main text
- The analysis of $SC|SM$, $SM|SC$ and $SC|SC$ is summarized in Table 2
- The analysis of $LM|LM$, $SM|LM$ and $LM|SM$ is summarized in Table 3

In this section we study the remaining combinations of $X|Y$ sets. For all such sets at least one between X and Y are of the LONG type (ie LC or LM). Due to the model's symmetry, we can limit our analysis to the sets where X is LONG, i.e. for LONG states during I_1 ($LC|Z$ and $LM|Z$, where $Z \in \{SM, SC, LM, LC\}$). Indeed, states $Z|LC$ and $Z|LM$ can be obtained respectively from states in $LC|Z$ and $LM|Z$ and by applying the symmetry principles.

The next theorem shows that the matrix form for these states allow us to determining all states that can exist in the parameter space. Indeed the entries of these matrices must satisfy properties (1-6) below.

Theorem 11 (Conditions for LONG states in I_1) *Any LONG state in I_1 satisfies:*

1. *If* $w^2 = 0 \Rightarrow x_A^2 \leq x_B^1, x_B^2 \leq x_A^1, y_A^2 \leq y_B^1$ *and* $y_B^2 \leq y_A^1$
2. *If* $w^2 = 0, y_A^2 = y_B^2 = 1 \Rightarrow x_A^1 \geq x_B^1$
3. *If* $w^2 = 1 \Rightarrow x_A^1 \geq x_B^1$
4. *If* $w^2 = 1$ *and* $x_A^2 = 1$ *or* $x_B^2 = 1 \Rightarrow x_A^1 \geq x_B^2, x_B^1 \geq x_A^2, y_A^1 \geq y_B^2$ *and* $y_B^1 \geq y_A^2$
5. *If* $w^2 = 1$ *and* $x_A^1 = 1$ *or* $x_B^1 = 1 \Rightarrow x_A^2 \geq x_B^1, x_B^2 \geq x_A^1, y_A^2 \geq y_B^1$ *and* $y_B^2 \geq y_A^1$
6. *If* V_2 *has all zero entries* $\Rightarrow x_A^1 \geq x_B^1$

Proof Due to Lemma 6 for any LONG state in I_1 both units turn are ON at time TD, and turn OFF at time $t^* + D$, for some $t^* \in [0, TD]$. Consequently both delayed synaptic variables exponentially decay during the interval I_2 starting from $t^* + 2D$. This leads to $s_A^{2-} = s_B^{2-} = e^{-(TR - t^* - 2D)/\tau_i}$. We notice that, since $t^* \geq 0$ we have

$$s_A^{2-} = s_B^{2-} \geq N_L^- \quad (15)$$

If $w^2 = 0$ (the hypothesis in 1.) the state is SHORT in I_2 (both units turn/are OFF at time TD). This means we can apply identies 13 on the interval I_1 and obtain

$$s_A^{1-} = s_B^{1-} \leq N^- \quad (16)$$

Inequalities 15 and 16 thus imply $s_A^{1-} \leq s_B^{2-}$ and $s_B^{1-} \leq s_A^{2-}$. By definition $x_A^1 = H(c-bs_B^{1-})$ and $x_B^2 = H(c-bs_A^{2-})$. Thus we have $x_B^2 \leq x_A^1$ (analogously we have $x_A^2 \leq x_B^1$). Moreover, $y_B^2 = H(ax_A^2+c-bs_A^{2-}) \leq H(ax_B^1+c-bs_B^{1-}) = y_A^1$ (and $y_A^2 \leq y_B^1$), proving 1.

One of the hypothesis of 2. is $w^2=0$. Thus we can apply identies 13 analogously to the previous case. Since $y_A^2 = y_B^2 = 1$, these identies lead to $s_A^{1-} = s_B^{1-} = N^-$. Condition $c \geq d$ guarantees that $x_A^1 = H(c-bN^-) \geq H(d-bN^-) = x_B^1$, thus proving 2.

We proceed by proving 3. Condition $w^2=1$ guarantees the corresponding states to be LONG in I_2. Due to the $2TR$ periodicity we have $s_A^{1-} = s_B^{1-} = e^{-(TR-s^*-2D)/\tau_i}$, for some $s^* \in [0, TD]$. This and $d \leq c$ imply $x_A^1 = H(c-bs_B^{1-}) \geq H(d-bs_A^{1-}) = x_B^1$, which proves 3.

Assuming the hypothesis of 4 (5) at least one unit turns ON at time TD (0). Lemma 6 thus implies $s^* = 0$ ($t^* = 0$). Therefore we have that $s_A^{1-} = s_B^{1-} = N_L^-$ ($s_A^{2-} = s_B^{2-} = N_L^-$), which implies $s_A^{1-} \leq s_B^{2-}$ and $s_B^{1-} \leq s_A^{2-}$ ($s_A^{2-} \leq s_B^{1-}$ and $s_B^{2-} \leq s_A^{1-}$). Using a proof similar to 1 we conclude 4 (5).

Assuming the hypothesis of 6. both units are OFF in I_2. Therefore, both delayed synaptic variables decay monotonically starting from time $t = t^*+2D$ until time $t=2TR$. For the $2TR$ periodicity we thus have $s_A^{1-} = s_B^{1-} = e^{-(2TR-t^*-2D)/\tau_i}$. This, $d \leq c$ and the definition of x_A^1 and x_B^1 yield 6. □

We applied Theorem 11 to investigate the possible combinations of states in all remaining sets $LC|Z$ and $LM|Z$, where $Z \in \{SM, SC, LM, LC\}$. We subdivide this analysis in the following cases.

Sets LM|SC and LM|LC - Any state ψ in either of these two sets is LONG and MAIN in I_1, and CONNECT in I_2. The LONG condition in I_1 implies that (a) both units are ON at time $\beta = TD$, and (b) $a-bs_A^{1+} \geq \theta$ and $a-bs_B^{1+} \geq \theta$. Condition (a) implies that V_1 must satisfy one of M_{1-3} during the interval I_1. From (b) we obtain $w^1=1$. As shown in the proof of property 5. above, we have that $s_B^{2\pm} = s_A^{2\pm} = N_L^{\pm}$. The CONNECT condition in I_2 implies that ψ must satisfy one of conditions C_{1-5}. However, since $d \leq c$, we must have $x_A^2 = H(d-bN_L^-) \leq H(c-bN_L^-) \leq x_B^2$ and $z_A^2 = H(a+d-bN_L^-) \leq H(a+c-bN_L^-) \leq z_B^2$. This excluded conditions the states satifying conditions C_1 and C_4 in I_2. Property 2. above guarantees that $LM|SC$ states satisfying condition M_3 in I_1 and C_2 or C_5 in I_2 cannot exist. The remaining set of $LM|SC$ states can exist in the parameter space and their name and matrix are given in in the first two rows of Table 4. We numerically verified their existence by finding a parameter set for which they are stable using linear programming on their sets of existing conditions and by simulating their dynamics. For states in $LM|LC$ we notice that, since they are LONG in I_2, they cannot satisfy condition C_3 in this interval (both units would otherwise be OFF at time $TR+TD$). Due to properties 3. and 5. above none of remaining states (the ones satisfying conditions C_2 and C_5) can exist. Therefore, no $LM|LC$ state can exist.

Sets LC|SC, LC|LC and LC|SM - Any state in either of these two sets is LONG and CONNECT in I_1. The LONG condition implies that both units are ON at time $\beta = TD$, thus excluding CONNECT conditions C_3 or C_4 in I_1. Furthermore, as shown in the case of $LM|LC$ (previous case), this LONG condition also excludes

$AScIL$	$ScASL^*$	$ScIL^*$	$AScIDL^*$	$ScASDL^*$	$ScIDL$	$ScASDL_2^*$
11110010<br>11111110	11110000<br>11110010	11110010<br>11110010	11110010<br>01111110	11110000<br>01110010	11110010<br>01110010	0110000<br>1110010
$D_3^- \geq \theta$<br>$C_7^- \geq \theta$<br>$D_5^- < \theta$<br>$D_2^+ \geq \theta$	$C_3^- \geq \theta$<br>$D_5^+ < \theta$<br>$D_3^- < \theta$<br>$D_3^+ \geq \theta$<br>$D_8^- \geq \theta$	$C_3^- \geq \theta$<br>$D_5^+ \geq \theta$<br>$D_3^- < \theta$<br>$D_3^+ \geq \theta$<br>$D_7^- \geq \theta$	$D_3^- \geq \theta$<br>$D_5^- < \theta$<br>$D_5^+ \geq \theta$<br>$C_5^- \geq \theta$<br>$D_7^- < \theta$	$C_3^- \geq \theta$<br>$D_5^+ < \theta$<br>$D_3^- < \theta$<br>$D_3^+ \geq \theta$<br>$D_8^- < \theta$<br>$D_2^- \geq \theta$	$C_3^- \geq \theta$<br>$D_5^+ \geq \theta$<br>$D_3^- < \theta$<br>$D_3^+ \geq \theta$<br>$D_7^- < \theta$<br>$C_5^- \geq \theta$	$C_3^- < \theta$<br>$C_6^- \geq \theta$<br>$D_3^+ \geq \theta$<br>$D_5^+ < \theta$<br>$D_8^- \geq \theta$
$D_{10} < \theta$<br>$C_{10} \geq \theta$	$C_{10} \geq \theta$	$D_{10} < \theta$<br>$C_{10} \geq \theta$	$D_{10} < \theta$<br>$C_{10} \geq \theta$	$C_{10} \geq \theta$	$D_{10} < \theta$<br>$C_{10} \geq \theta$	$C_{10} \geq \theta$

Table 4 Matrix form and existence conditions of $2TR$-periodic $LM|SC$ states (asymmetrical states in *).

CONNECT conditions C_1 and C_4 for $LC|SC$ and $LC|LC$ states in I_2, and conditions M_2 and M_4 for $LC|SM$ states in I_2. For states in $LC|LC$ we notice that, since they are LONG in I_2, they cannot satisfy condition C_3 in this interval (for an analogue reason of case $LM|LC$). Of the remaining states, the ones described by the following matrix forms cannot exist:

$$\begin{matrix} 1111 & 0000 \\ 0011 & 0010 \end{matrix} \quad \text{and} \quad \begin{matrix} 1111 & 0000 \\ 0011 & 1110 \end{matrix}$$

Indeed entries $w^1 = 1$ and $y_B^1 = 0$ imply respectively $a - bN^+ \geq \theta$ and $a - M_L^- + d < \theta$. These two conditions imply $d < be^{(D-TR)/\tau_i}(e^{(D-TR)/\tau_i} - 1) < 0$. This is absurd since by hypothesis we must have $TR > D$ and $d \geq 0$. Finally the application of properties 1-6 above reduces the number of possible states. The remaining set of $LM|SC$ states can exist in the parameter space and their name and matrix are given in the first two rows of Table 5.

$ScASDL_3^*$	$APcIDL^*$	$ScSDL^*$	$APcIL$	$ScASDL_4^*$	$ScASL_2^*$	$ZcIL^*$
11110010<br>00110010	11110010<br>00111110	11110000<br>00110000	11110011<br>00111111	00110000<br>11110010	00110000<br>00110010	00110010<br>00110010
$C_3^- \geq \theta$<br>$D_3^- < \theta$<br>$D_3^+ \geq \theta$<br>$D_5^- < \theta$<br>$D_5^+ \geq \theta$	$D_3^- \geq \theta$<br>$C_5^- < \theta$<br>$D_5^+ \geq \theta$	$D_3^+ < \theta$<br>$D_4^- \geq \theta$<br>$D_2^- < \theta$<br>$D_2^+ \geq \theta$	$D_3^- \geq \theta$<br>$D_5^- < \theta$<br>$D_5^+ \geq \theta$	$C_6^- < \theta$<br>$D_6^+ \geq \theta$<br>$D_5^+ < \theta$<br>$D_8^- \geq \theta$	See 17	See 18
$D_{10} < \theta$<br>$C_{10} \geq \theta$	$D_{10} < \theta$<br>$C_{10} \geq \theta$	$D_9 \geq \theta$	$D_{10} \geq \theta$	$C_{10} \geq \theta$		

Table 5 Matrix form and existence conditions of $2TR$-periodic $LC|SC$, $LC|LC$ and $LC|SM$ states (asymmetrical states in *).

The last two rows of Tables 4 and 5 show the conditions of existence of the corresponding $LM|SC$, $LC|SC$, $LC|LC$ and $LC|SM$ states. Determining these is straightforward in most cases. Indeed, it requires using formulas 9 and 13 on the definition of the entries of each matrix form, and application of simplifications, analogously to the previous considered cases, except for $ScASL_2$ and $ZcIL$ (see Table 5). These two need special attention, because they satisfy property C_5 in I_1, we cannot apply the formulas 9 and 13. For $ZcIL$ the A unit turns ON before the B units in I_1 ($t^* < s^*$), because both synaptic variables s_A and s_B evolve equally during in this interval (on the fast time scale) and the total input to the A unit

is greater than the one to the B unit at time t^* , i.e. $c-bs_B(t^*) \le d-bs_A(t^*)$. For $ScASL_2$ the two synaptic variables evolve differently on I_1, which may lead to $t^* < s^*$ or $t^* \ge s^*$. Later we will show that case $t^* \ge s^*$ cannot exist. Lastly there are three degenerate states that exist only under $\tau = 0$, which cannot be numerically simulated. These states conclude all set of existing $2TR$-periodic states in the system under the case $TR \le TD + D$ and $D \ge TD$.

We proceed by describing the existence conditions for $ScASL_2$ for $t^* < s^*$ and state $ZcIL$.

- Case $ScASL_2$ for $t^* < s^*$ - This state satisfies conditions C_5 on I_1 and C_3 on I_2. Since $y_B^2 = 1$ and $w^2 = 0$ the B unit turns OFF at time $TR{+}TD$. Due to Lemma 6 the synaptic variable $s_B(t)$ exponentially decays starting from time $TR{+}TD{+}D$ and due to the $2TR$-periodicity we must have $s_B(t) = e^{-(TR+t-TD-D)/\tau_i}$, for $t \in [0, TD]$. From this we obtain $s_B(0) = N^-$ and $s_B(TD) = N^+$. Condition C_5 on I_1 with $t^* < s^*$ requires $C_3^- = c - bN^- < \theta$ and $c - bN^+ \ge \theta$. The turning ON time for the A unit in I_1 is therefore given by

$$t^* = s_B^{-1}((c-\theta)/b)) = TR - TD - D - \tau_i \log((c-b)/\theta).$$

From Lemma 6 and from $t^* < s^*$ we obtain that both units instantaneously turn OFF at time $t^*{-}2D$. Thus the synaptic variable $s_A(t)$ and $s_B(t)$ exponentially decay following the same dynamics on the slow time scale starting from time $t^*{+}2D$. This leads to $s_A(t) = s_B(t) = e^{-(t-t^*-2D)/\tau_i}$, for $t \in [TR, TR{+}TD]$. Moreover, since the A unit is OFF in I_2 and due to the $2TR$-periodicity we have $s_A(t) = e^{-(2TR+t-t^*-2D)/\tau_i}$, for $t \in [0, TD]$. These properties yield $s_A^{2+} = s_A(TR{+}TD) = e^{(D-2TD)/\tau_i}(c-\theta)/b$ and $s_A^{1+} = s_A(TD) = e^{(D-2TD-TR)/\tau_i}(c-\theta)/b$. To complete the conditions C_5 on I_1 we need to guarantee that the B unit turns ON at some time $s^* \ge t^* \in [0, TD]$. These are equivalent to $d - bs_A(t^*) < \theta$ and $a - bs_A^{1+} + d \ge \theta$, which can respectively be rewritten as $d - be^{2(D-TR)/\tau_i} < \theta$ and $a - Lc + d \ge (1-L)\theta$, where $L = e^{(D-2TD-TR)/\tau_i}$. Condition C_3 on I_2 requires $c - bs_A^{2-} < \theta$ and $c - bs_A^{2+} \ge \theta$. This first of these conditions is not necessary, since it is implied by the already existing condition $C_3^- < \theta$ (since $s_A^{2+} \ge N^-$). The second is equivalent to $(c-\theta)(1-K) \ge 0$, with $K = e^{(D-2TD)/\tau_i}$, which occurs if and only if $D \le 2TD$ (since $c \ge \theta$). To complete condition C_3 we need to guarantee that the A unit stays OFF in I_2, ie that $a - bs_B^{2+} + d < \theta$, which is equivalent to $a - Kc + d < (1-K)\theta$. Finally, the last condition derives from $w^1 = 1$ ($w^2 = 0$ is authomatically guaranteed since the A is OFF at time $TR{+}TD$), ie $C_{10} = a - bN^+ \ge \theta$. This guarantees also $a - bs_A^{1+} = a - bM_L^+ \ge \theta$, since $M_L^+ \le N^+$. Thus in summary the list of conditions for this state is:

$$\begin{aligned} C_3^- &< \theta \\ C_{10} &\ge \theta \\ D &\le 2TD \\ d &< \theta - be^{2(D-TR)/\tau_i} \\ a - Lc + d &\ge (1-L)\theta \\ a - Kc + d &< (1-K)\theta \end{aligned} \tag{17}$$

- Case $ZcIL$. This state satisfies conditions C_5 during both intervals I_1 and I_2. Both units turn OFF at time $TR+TD$. Lemma 6 implies that both synaptic variables exponentially decay starting from time $TR+TD+D$ and due to the $2TR$-periodicity we must have $s_A(t)=s_B(t)=e^{-(TR+t-TD-D)/\tau_i}$, for $t \in [0, TD]$. From the A unit turns ON before the B unit in interval I_1, precisely at time t^*, and both units turn OFF at time t^*+D for lemma 6. Thus the delayed synaptic variables exponentially decay from time t^*+2D and we have $s_A(t)= s_B(t)=e^{-(t-t^*-2D)/\tau_i}$, for $t \in [TR, TR+TD]$. Thus both variables evolve equally (on the slow time scale) respectively on I_1 and on I_2. Although condition C_5 on both intervals could lead to potentially 4 cases, we only have one case to consider, the A (B) unit turns ON before the B (A) unit in interval I_1 (I_2). Analogously to the case $ScASL_2$, condition C_5 on I_1 requires $C_3^- = c - bN^- < \theta$ and $c - bN^+ \geq \theta$, and t^* is given by

$$t^* = s_B^{-1}((c-\theta)/b)) = TR - TD - D - \tau_i \log((c-b)/\theta).$$

As in case $ScASL_2$ condition C_3 on I_2 requires $D \leq 2TD$. To complete the conditions C_5 we require $a - bs_B^{2+} + d \geq \theta$, which is equivalent to $a - Kc + d \geq \theta(1-K)$. Lastly, we need to guarantee $w^1 = 1$ and $w^2 = 0$, which are equivalent respectively to $C_{10} \geq \theta$ and $a - Kc < \theta(1-K)$ (ie $a - bs_B^{2+} < \theta$). Thus in summary the list of conditions for this states are:

$$\begin{aligned} C_3^- &< \theta \\ C_{10} &\geq \theta \\ D &\leq 2TD \\ a - Kc + d &\geq (1-K)\theta \\ a - Kc &< (1-K)\theta \end{aligned} \tag{18}$$

Lastly, we show that the following three states may exist only if $\tau = 0$ (degenerate cases). These states complete all the existing states after application of conditions 11. This finally concludes the existence conditions for all $2TR$-periodic states in the system.

$$ScASL_2 = \begin{bmatrix} 0011 & 0000 \\ 0011 & 0010 \end{bmatrix} \text{ for } t^* \geq s^*, ZcIL_2 = \begin{bmatrix} 0011 & 0011 \\ 0011 & 0011 \end{bmatrix} \text{ and } ZcSL = \begin{bmatrix} 0011 & 0000 \\ 0011 & 0000 \end{bmatrix}.$$

We show that these three states cannot exist unless $\tau = 0$ (degenerate for the model). Firstly we note that each case satisfies condition C_5 on interval I_1, so that A turns ON at time t^* and B turns ON at time s^*, for some $t^*, s^* \in [0, TD]$. Next we divide the proof for the three cases above:

1 $ZcSL$ - This state satisfies condition C_5 on interval I_1. That A turns ON at time t^* and B turns ON at time s^*, for some $t^*, s^* \in I_1$. Since both units turn OFF instantaneously and at the same time in $\mathbb{R} - I$, both synaptic variables evolve equally (on the slow time scale) in I_1. Therefore we have that $t^* \geq s^*$.

Let us rename $t_1 = t^*$. On the fast time scale r the variable $s_A(t)$ converges to 1 at time t_1 following

$$\begin{aligned} s_A(r)' &= (1 - s_A(r)) \\ s_A(0) &= s_A(t_1) \end{aligned}$$

where $'$ is the derivative with respect to the fast time scale r. The analytic solution is given by $s_A(r) = 1 - (1 - s_A(t_1))e^{-r}$. Therefore, this equation describes the (fast) evolution of the delayed synaptic variable $s_A(t - D)$ at time $t = t_1 + D$. At this time the B unit instantaneously turn OFF, since $a - bs_A(t - D) \rightarrow a - b < \theta$ for hypothesis U_1 in Remark 2.1 in the main text. We can use the equation for $s_A(r)$ and derive the precise time when u_B turns OFF. Since $a - bs_A(t_1) \geq \theta$ and $a - b < \theta$ there $\exists s^* \in [s_A(t_1), 1]$ for which $a - bs^* = \theta$. Given the evolution of s_A, the time when B unit turns OFF is precisely $r^* = r^*(t_1) = \log((1 - s_A(t_1))/(1 - s^*))$. The latter equality highlights the dependence on t_1. By adding the delay and returning to the normal time scale the B unit turns OFF at time $t_1 + D + \delta(t_1)$, where $\delta(t_1) = \tau r^*$. Since the dynamics of delayed synaptic variable $s_B(t - D)$ is dictated by the B unit activity, it starts to exponentially decay at time $t_1 + 2D + \delta(t_1)$. Thus it evolves according to $s_B(t - D) = e^{-(t - t_1 - 2D - \delta(t_1))/\tau_i}$, for $t \in I_3 = [2TR, 2TR + TD]$. A necessary condition for this state to exist is that it satisfies C_5 is that A turns ON within I_3. This occurs if and only if $c - bs_B(TR - D) < \theta$ and $c - bs_B(TR + TD - D) \geq \theta$. This is equivalent to $\exists t_2 \in I_3^0$ (the open set) such that $c - bs_B(t_2 - D) = \theta$. From the analytic solution of $s_B(t_2 - D)$ we can solve this equation and obtain $t_2 = t_1 + \delta(t_1) + Q$, where $Q = 2D - \log((c - \theta)/b)$ is a constant. By repeating this process across subsequent the periodic intervals $I_k = [2(k-1)TR, 2(k-1)TR + TD]$ we obtain that the k turning ON time for the A unit is given by the map

$$t_{k+1} = t_k + \delta(t_k) + Q. \tag{19}$$

Since we are interested in the limit $\tau \rightarrow 0$ and on TR-periodic solution it must be that $2TR = Q$. However, assuming true this condition and $\tau > 0$ arbitrarily small, this map shows that the A unit turns ON with after a small delay δ across subsequent intervals I_k (ie the map has no fixed point). Therefore, $ZcIL$ cannot exist.

2 $ScASL_2$ for $s^* < t^*$ - The proof is analogous to the case above ($ZcSL$) after swapping the A and B units. Briefly, if the B unit turns ON at time $t_1 \in [0, TD]$ the A unit turns OFF at time $t_1 + D + \delta(t_1)$, where $\delta(t_1) \sim \tau$. This means that s_A evolves according to $s_A(t - D) = e^{-(t - t_1 - 2D - \delta(t_1))/\tau_i}$, for $t \in I_3 = [2TR, 2TR + TD]$. The k turning ON time for the B unit is given by the map 19, where $Q = 2D - \log((d - \theta)/b)$. As in the previous case, $ScASL_2$ cannot exist because this map has no fixed point unless $\tau = 0$.

3 $ScASL_2$ - This state satisfies condition C_5 in both intervals I_1 and I_2. Let us call t_1 and s_1 the turning ON times for A and B in I_1 respectively, and t_2 and s_2 the turning ON times for B and A in I_2 respectively. On the slow time scale both (delayed) synaptic variables s_A and s_B evolve equally in I_1

and I_2, because both units turn OFF instantaneously and at the same time in $\mathbb{R}-I$ for Lemma 6. Since $d \le c$ the total input to A is greater than the one to B for $t \in I_1$, ie $c-bs_B(t-D) \ge d-bs_A(t-D)$, which leads to $t_1 \le s_1$. Analogous considerations lead to $t_2 \le s_2$. Moreover, it turns out that $t_1 = t_2 - TR$. Indeed, WLOG suppose that $t_2-TR > t_1$. Since $c-bs_B(t_1-D)=\theta$ and due to the monotonic decay of the delayed synaptic variables in I_1 we must have $c-bs_B(t_2-TR-D) \ge \theta$. Moreover, since $c-bs_A(t_2-D)=\theta$ we have that $s_B(t_2-TR-D) < s_A(t_2-D)$. Since A turns ON at time t_1 the B unit turns OFF at time $t_1+D+\delta(t_1)$, where $\delta(t_1) \sim \tau$. Thus $s_A(t-D) = e^{-(t-t_1-2D-\delta(t_1))/\tau_i}$, for $t \in I_2$. Similarly, since B turns ON at time t_2 and for the $2TR$-periodicity we have that $s_B(t-D) = e^{-(2TR+t-t_2-2D-\delta(t_1))/\tau_i}$, for $t \in I_1$. On the slow time scale ($\tau \to 0$) these identities evaluated at time t_2 imply $s_B(t_2-TR-D) = e^{(-TR+2D)/\tau_i}$ and $s_A(t_2-D) = e^{(t_1-t_2+2D)/\tau_i}$. Due to the hypothesis $t_2 - TR > t_1$ the latter lead to $s_B(t_2-TR-D) \ge s_A(t_2-D)$, which is absurd. Therefore we have that $t_1 = t_2 - TR$. This in turn leads to $s_B(t-D) = e^{-(t-t_1-TR-2D-\delta(t_1))/\tau_i}$, for $t \in I_3 = [2TR, 2TR+TD]$. Due to the $2TR$ periodicity the second turning ON time for A (after t_1) must be at a time $t_3 = t_1 + 2TR \in I_3$ such that $c-bs_B(t_3-D)=\theta$. From the analytic solution of $s_B(t_2-D)$ we obtain $t_3 = t_1 + \delta(t_1) + Q$, where $Q = TR + 2D - \log((c-\theta)/b)$ is a constant. Thus the k turning ON time for the A unit is given by the map 19. Due to the dependence on τ, this map has no fixed point unless $\tau = 0$, thus proving that $ScASL_2$ cannot exist.

1.16 2D visualization of SHORT CONNECT and LONG MAIN states

The existence conditions all these states can be visualized as a 2D parameter projection, similar to Figure 9B in the main text for SHORT MAIN states. Figure 3A,C show respectively the regions of existence of SHORT CONNECT and LONG MAIN state when varying parameters (c, DF), and the remaining parameters have been fixed to satisfy $TD < D$ and $TD + D < TR$ (the name and existence conditions of these states are in Table 2 and 3 in Supplementary Material 1.13 and 1.14). In panel A. SHORT MAIN states are shown in dark blue to help the comparison with Figure 9B in the main text (same parameters). Figure 3B,D show time histories for the SHORT CONNECT state $APcAS$ and the LONG MAIN state SDL.

Remark 1.2 (CONNECT states) By comparing Figure 3A with Figure 9B in the main text (same parameters) we note that the union of the regions of existence of MAIN states is larger than the one of CONNECT states, hence why we call the first group MAIN. In addition SHORT CONNECT states connect branches of SHORT MAIN states, hence why we called them CONNECT.

1.17 $2TR$-periodic states for $D < TD$ and $TD + D < TR$ and $a + d - b < \theta$

Theorem 12 *Let us now consider $2TR$-periodic states for $D < TD$, $TD + D < TR$ and $a+d-b < \theta$, and define $L_1 = [0, D]$ and $L_2 = [TR, TR+D]$. The synaptic quantities defining the entries of the matrix form in L_1 and L_2 are given by*

$$s_A^{2\pm} = s_B^{1\pm} = N^{\pm}, \quad s_A^{1\pm} = \begin{cases} R^{\pm} & \text{if } z_A^2 = 1 \\ M^{\pm} & \text{otherwise} \end{cases} \quad \text{and} \quad s_B^{2\pm} = \begin{cases} R^{\pm} & \text{if } z_B^1 = 1 \\ M^{\pm} & \text{otherwise} \end{cases} \tag{20}$$

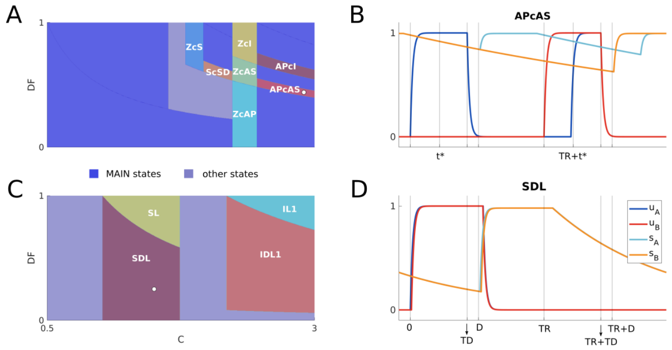

Figure 3 Visualization of SHORT CONNECT and LONG MAIN states. Panels A. and C. show regions of existence respectively for SHORT CONNECT and LONG MAIN states. SHORT MAIN states in panel A. are shown in dark blue. States that are neither SHORT MAIN, SHORT CONNECT or LONG MAIN are shown in light blue. For the same parameters as in A. and D., panels B. and D. respectively show example time histories for a SHORT CONNECT state ($APcAS$) and a LONG MAIN state (SDL) with fixed (c, DF) shown by white dots in A. and D. In panel A. the parameters are the same as in Figure 9B in the main text . In panel C. parameters are the same as in A. except for $\tau_i = 0.05$ and $a = 2$.

Where $R^- = e^{-(TR-2D)/\tau_i}$ *and* $R^+ = e^{-(TR-D)/\tau_i}$*. Quantities* $M^\pm$ *and* $N^\pm$ *were defined in equations 14 in the main text .*

Proof Since A (B) is ON in $[0, TD]$ ($[0, TR+TD]$) and turn OFF instantaneously at time TD ($TR+TD$) due to property in equation 18 in the main text . The synaptic variable s_A (s_B) thus exponentially decays on the slow time scale starting from time TD ($TR+TD$) and ending at time TR ($2TR$). Due to this and to the $2TR$-periodicity the delayed synaptic variable $s_A(t-D)$ ($s_B(t-D)$) evaluated at times TR and $TR+TD$ (0 and TD) are equal to $N^\pm$, which proves the first identity of the theorem. If $z_A^2 = 1$ the A unit is ON in L_2 and turns OFF instantaneously at time $TR+D$ for both MAIN or CONNECT states. Thus the synaptic variable s_A slowly decays starting from time $TR+D$ until the A unit turns ON at time $2TR$. This implies $s_A(t-D) = e^{-(t-TR-2D)}$, for $t \in [2TR, 2TR+D]$. The $2TR$-periodicity leads to $s_A^{1-} = s_A(2TR-D) = e^{-(TR-2D)/\tau_i}$ and $s_A^{1+} = s_A(2TR) = e^{-(TR-D)/\tau_i}$, proving the second identity of the theorem. The proof of the third identity is analogous to the previous one. □

1.18 Case $TD+D \geq TR$

The condition $TD+D < TR$ enabled us to obtain a complete classification of network states via the application of Lemma 2. Each of these states (except for $AScI$) has the same existence conditions given in the two tables also for $TD+D \geq TR$ with two adjustments. More precisely, if $TD+D \geq TR$ and $2D < TR$ we must replace the quantity N^- with unity in the existing condition C_7^- in Table 5 in the main text . If $2D \geq TR$ (which implies $TD+D \geq TR$) we must replace the quantity R^- with unity in the existing R_6^- and R_7^- in Table 6 in the main text This is valid for all states except for $AScI$, for which we additionally need to impose that the turning ON time t^* for the B unit in $[0, D]$, or equivalently the turning ON time $TR+t^*$ for the A unit in $[TR, TR+D]$, satisfies

$$t^* + D < TR.$$

Where t^* is given by the solution of $a - be^{-(TR-D-t^*)/\tau_i} + d = \theta$. We now proof that state $AScI$ cannot exist if $t^* + D \geq TR$ and $D < TD$, where $t^* \in [0, D]$ ($t^* + TR \in [TR, TR + D]$) is the turning ON time for the B (A) unit in the interval I_1 (I_2). We need to show that the B (A) unit cannot be OFF for $t < t^*$ ($t < t^* + TR$) and ON for $D \geq t > t^*$ ($D + TR \geq t > t^* + TR$). By absurd suppose the contrary. We now determine the dynamics of the the delayed synaptic variable $s_B(t-D)$ during the interval $[TR, TR+D]$. The B unit turns ON at time $t^* \geq TR - D$ and is ON in I_2 (due to property in equation 18 in the main text). These properties and the $2TR$-periodicity of $AScI$ imply that $s_B(t-D)$ evolves according to

$$s_B(t-D) = e^{-(TR+t-TD-D))/\tau_i}, \quad \forall t \in [TR, TR+t^*).$$

Evaluating this equation at time $t_1 = TR$ leads to $s_B(t_1 - D) = e^{-(2TR-TD-D))/\tau_i}$. Secondly we have that

$$s_B(t-D) = e^{-(t-2D))/\tau_i}, \quad \forall t \in (2D, TR+D].$$

Evaluating this equation at time $t_2 = TR + D$ leads to $s_B(t_2 - D) = e^{-(TR-D))/\tau_i}$. This implies:

$$s_B(t_1 - D) \leq s_B(t_2 - D).$$

However by hypothesis A is OFF at time $t_1 < t^* + TR$ and ON at time $t_2 > t^* + TR$, i.e. t$a + d - bs_B(t_1 - D) < \theta$ and $a + d - bs_B(t_2 - D) \geq \theta$, which is absurd.
